# Hilbert-curve assisted structure embedding method

**DOI:** 10.1186/s13321-024-00850-z

**Published:** 2024-07-29

**Authors:** Gergely Zahoránszky-Kőhalmi, Kanny K. Wan, Alexander G. Godfrey

**Affiliations:** National Center for Advancing Translational Sciences (NCATS/NIH), 9800 Medical Center Dr., Rockville, MD 20850 USA

**Keywords:** Chemical space embedding, Clustering, Hilbert-curve, Scaffold-Keys, HCASE, Dimension reduction

## Abstract

**Motivation:**

Chemical space embedding methods are widely utilized in various research settings for dimensional reduction, clustering and effective visualization. The maps generated by the embedding process can provide valuable insight to medicinal chemists in terms of the relationships between structural, physicochemical and biological properties of compounds. However, these maps are known to be difficult to interpret, and the ‘‘landscape’’ on the map is prone to ‘‘rearrangement’’ when embedding different sets of compounds.

**Results:**

In this study we present the Hilbert-Curve Assisted Space Embedding (HCASE) method which was designed to create maps by organizing structures according to a logic familiar to medicinal chemists. First, a chemical space is created with the help of a set of ‘‘reference scaffolds’’. These scaffolds are sorted according to the medicinal chemistry inspired Scaffold-Key algorithm found in prior art. Next, the ordered scaffolds are mapped to a line which is folded into a higher dimensional (here: 2D) space. The intricately folded line is referred to as a pseudo-Hilbert-Curve. The embedding of a compound happens by locating its most similar reference scaffold in the pseudo-Hilbert-Curve and assuming the respective position. Through a series of experiments, we demonstrate the properties of the maps generated by the HCASE method. Subjects of embeddings were compounds of the DrugBank and CANVASS libraries, and the chemical spaces were defined by scaffolds extracted from the ChEMBL database.

**Scientific contribution:**

The novelty of HCASE method lies in generating robust and intuitive chemical space embeddings that are reflective of a medicinal chemist’s reasoning, and the precedential use of space filling (Hilbert) curve in the process.

**Availability:**

https://github.com/ncats/hcase

**Graphical Abstract:**

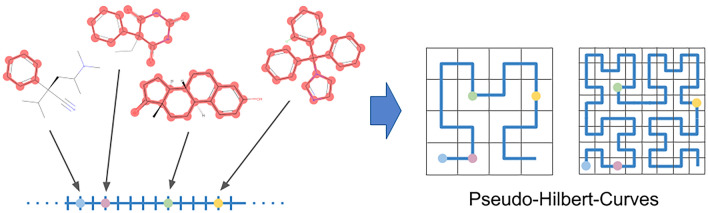

**Supplementary Information:**

The online version contains supplementary material available at 10.1186/s13321-024-00850-z.

## Introduction

Embedding molecular structures into a chemical space is a versatile technique that is central to a wide range of data analysis scenarios in cheminformatics. Methods, like principal component analysis (PCA) [1], multi-dimensional scaling (MDS) [2], *t*-Stochastic Neighbor Embedding (*t*-SNE) [3], Uniform Manifold Approximation and Projection (UMAP) [4] and the self-organizing maps (SOM) method [5], help reduce the dimensionality of data to facilitate subsequent cluster analyses or to provide insightful visualizations. While most of these methods can be performed in a relatively straightforward manner from an operational point of view, this somewhat deceiving simplicity comes at the cost of certain limitations to applicability and interpretability.

For instance, PCA can only analyze linear relations present in the data at hand. This limitation is overcome by non-linear approaches, such as the related multi-dimensional scaling (MDS) and manifold-supported methods [6], such as *t*-SNE and UMAP methods.

All these non-linear methods, except for MDS are challenged with the means of computing the distance between the embedded datapoints. Interpretation of the underlying organizing principle of the embedded structures is convoluted for all known space-embedding methods. Also, the chemical space created by both linear and non-linear methods is influenced by the dataset at hand. This affects the interpretation of results and makes the comparison of individually embedded datasets quite difficult. While this can be addressed to some extent by merging the datasets before the embedding process, this solution is not robust against the incorporation of additional data.

## Background

The aim of performing a chemical space embedding analysis is to create a “map” of compounds. A compound’s position in this map ideally should reflect structural and/or other properties of interest (e.g., physicochemical properties), and as a result, the relative position of compounds within the map should be reflective of their similarities in these properties. A chemical space map can help medicinal chemists make quick, intuitive analyses about the structure and properties of compounds in a project based on their location in the map. For example, one would expect that compounds of related chemotype in a structure–activity-relationship (SAR) series will be placed closely on the map, whereas dissimilar chemotypes farther apart.

**Fig. 1:**
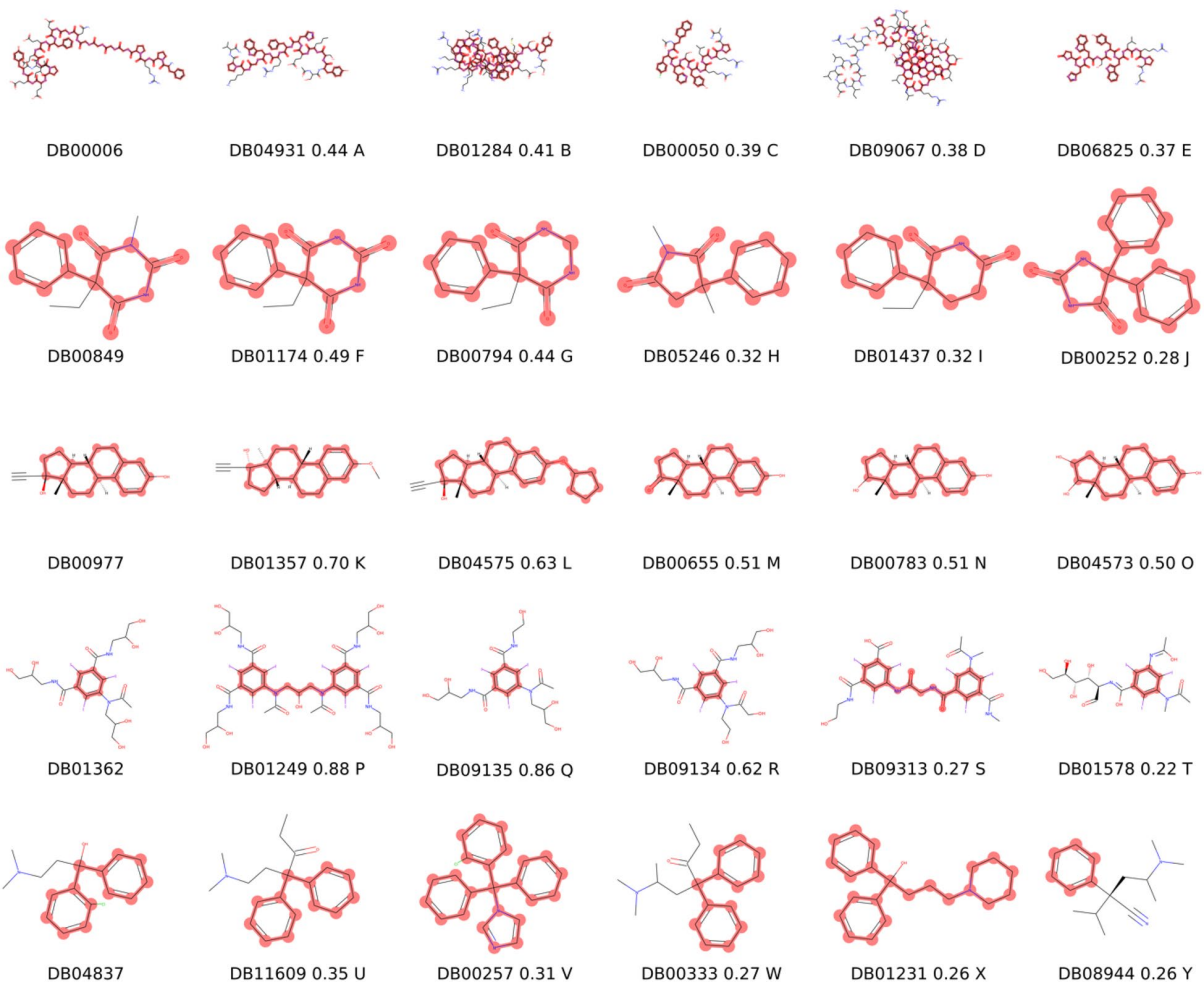
KNNs of randomly selected molecules. First column contains the query structures and subsequent columns contain the *k* = 5 NNs in decreasing order of similarity. Tanimoto-similarity was computed using Morgan-fingerprints, radius = 3, length = 2048. The value of Tanimoto-similarity coefficient and the label of compounds are shown after the compound IDs for NNs. The BMSs of compounds are highlighted by red

While creating such maps is entirely possible with existing methods, e.g., with *t*-SNE, medicinal chemists and data analysts are challenged with the interpretations of the results. For demonstration purposes, a map (embedding) of approved drugs has been generated using the *t*-SNE algorithm. In order to demonstrate the chemical space embedding process, five drug molecules were selected randomly, as well as the five nearest neighbors (NNs), i.e., structurally most similar five compounds of each (see: Fig. 1).

**Fig. 2:**
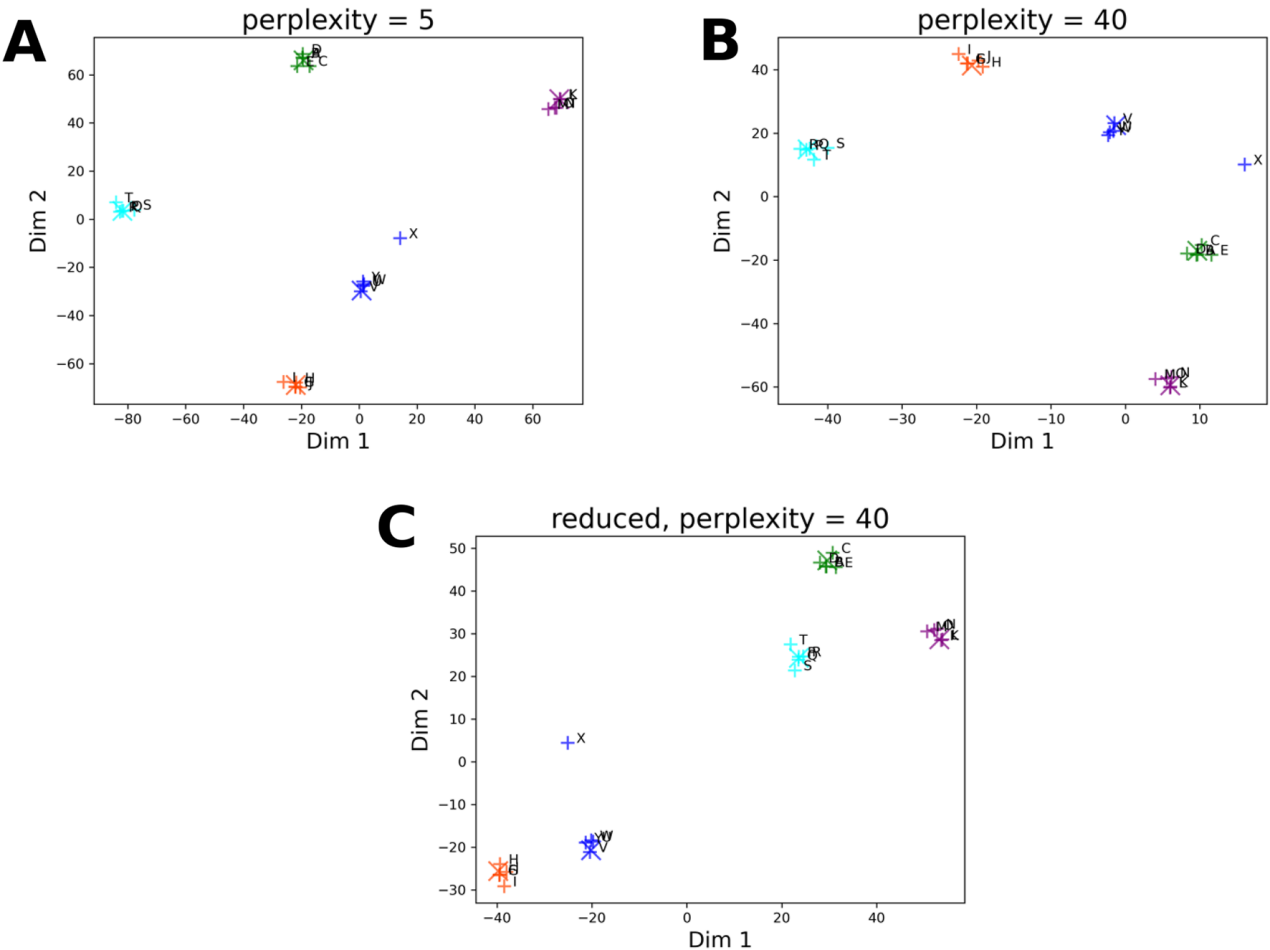
Maps generated by *t*-SNE Analysis of Drug Molecules. Embedding of DrugBank molecules performed by the original *t*-SNE algorithm at various perplexity values and repeating the embedding with a 90% sized subset of drug molecules. The randomly selected five molecules are marked by enlarged (X) symbol. Green: DB00006, orange: DB00849, purple: DB00977, aqua: DB01362, blue: DB04837. The NNs of each molecule are indicated by ( +) symbol with matching color. Molecules are labeled according to Fig. 1. **A** *t*-SNE embedding of drug compounds, perplexity = 5. **B** *t*-SNE embedding of drug compounds, perplexity = 40. **C** 90% sized subset of drug compounds, perplexity = 40

As shown on Fig. 2a, the resultant map shows a great clustering and separation of similar and dissimilar molecules, respectively, as one would expect. However, from a medicinal chemist’s standpoint some important aspects of the data analysis remain hidden.

For instance, a chemist might want to know if certain regions of this map encode a certain type of chemotype, e.g., based on size, complexity and so on. Unfortunately, maps generated with existing embedding methods provide little help to chemists in this regard. Furthermore, generating a map often requires setting certain non-intuitive parameters, like the *perplexity* in the case of *t*-SNE, which many chemists may not be familiar with. This parameter influences which compounds should be close or farther apart in the resultant chemical space map [7]. The choice of the parameter can affect the layout of the map, and often in an unpredictable manner, as it is demonstrated on Fig. 2b vs. Figure 2a.

Finally, the layout of the map generated by the same space embedding method can be greatly altered when one adds or removes molecules when repeating the embedding process, as demonstrated on Fig. 2b–c*.* This makes it challenging to compare the embedding of a library that is changing over time. The only difference between the two maps is that the Fig. 2c was generated using 90% of the molecules of the embedding used in Fig. 2b and the same highlighted molecules. The two maps show little resemblance despite the relatively small change in input. Further information regarding the embedding process of drug molecules with the t-SNE algorithm is provided in Section “*Embedding of Drug Molecules with t-SNE Algorithm*” and *Fig. S1-S2* in Supplementary Information (SI).

In this study we introduce a novel space embedding method that addresses the above detailed challenges of existing space embedding methods in creating an intuitive chemical space.

## Related methods

Besides the general space embedding methods, chemistry specific space embedding methods exist [8]. The PCA-based “ChemGPS” [9] and Molecular Quantum Number [10] methods address the issue of creating embedding via a mechanism that is not influenced by the dataset at hand [11]. The SOM-related “generative topographic mapping GTM” method by *Lin *et al*.* [12], and the “constellation plots” [13] take advantage of scaffold-compound relations to enhance the embedding. Furthermore, the GTM method defines a grid with the help of “landscape structures” that guides the subsequent embedding of compounds. While the GTM and constellation plot methods indeed address many challenges, the organizing principle of the compounds, or landscape structures of both methods is not based on a medicinal chemistry inspired ruleset. A recent method (TMAP) [14] uses a combination of nearest neighbor and minimal-spanning trees and force-based network layout to generate embedding, but the organizing principle of the method is still based on heuristics. Thus, it cannot guarantee that regions in the resultant map can be intuitively interpreted.

The above methods intended to solve known challenges related to chemical space embedding, but none of them have solved all the aforementioned challenges to a degree that would result in intuitive chemical space maps for medicinal chemists. Nevertheless, these methods gave rise to many important concepts and aspects that are utilized in this study.

In this proof-of-concept study, we set forth criteria for a chemical space embedding method that provides intuitive results and easy interpretation from a medicinal chemistry point of view and devised a new method that produces results reflective of such characteristics. In the following section, the new method is introduced in details and its applicability is demonstrated via a set of experiments.

## Computational methods and datasets

In this section, we detail the development of a novel chemical space embedding method and introduce an essential component of it from prior art, the Scaffold-Key (SK) method. The description of other analytical methods and datasets involved in this study is also provided below.

### Scaffold-Key (SK) algorithm from prior art

The general idea behind the SK algorithm was to provide an ordering of BMSs to mimic the thinking process of a medicinal chemist in analyzing BMSs based on their size, complexity, and chemical composition. Furthermore, the SK algorithm aimed to provide a distance measure that surpasses fingerprint-based distance measure between scaffolds, due to known limitations [19]. To this end, 32 so-called “Scaffold-Keys” were defined that each capture unique structural aspects of a given BMS. The definition behind these 32 keys defines the ruleset of the algorithm that is publicly disclosed in the original publication by Ertl [19]. The SK algorithm generates a 32-key SK for a given BMS which can be used to sort the BMSs or to define a distance measure between BMSs. Distance *d*_*SK*_(*i, j*) between a pair of SKs of respective BMSs *i* and *j* can be quantified with the help of their SK according to *Eq. *1 as defined by Ertl. *SK*_*i*_(*n*) and *SK*_*j*_(*n*) denote the value of the *n*^th^ key in the SK of BMS *i* and *j*, respectively.

1 $$d_{SK} (i,j) = \sum\limits_{n = 1}^{32} {\frac{{\sqrt {|SK_{i} (n) - SK_{j} (n)|^{3}} }}{n}}$$

Since the SK algorithm does not have a publicly available implementation it was necessary to create an in-house implementation based on the published ruleset. The implementation follows the ruleset as truthfully as possible, with the only exception that optionally, it is possible to generate the InChI-Key [22] of BMS as an extra (last) key on the top of the original 32 keys. Moreover, a few of the original rules were defined in a slightly vague manner, therefore we could only attempt to match those as closely as possible in light of insufficient information. Nevertheless, clarification of rules, where it was necessary, is provided in “*Appendix*” in SI. Implementation of the SK algorithm is publicly available as a source-code repository at: https://github.com/ncats/hcase [23].

SKs were generated with the in-house implementation of the SK algorithm, as well as the *d*_*SK*_ distances between BMSs.

### Development of the intuitive structure embedding methods

#### Rationale

Here, we define a set of criterions underpinning a method that is capable of providing a chemical space embedding so that the outcome of the analysis can be interpreted intuitively from a medicinal chemistry point of view:

1. Coordinates of structures generated by space embedding process is not influenced by the structural features of other compounds in the compound set to be embedded
2. Similar chemotypes should be placed closely on the generated map, closely placed coordinates should be similar chemotypes [46]
3. Mapping of structures to coordinates is deterministic, therefore reproducible
4. The organizing principle behind placing chemotypes on the map should rely on a well-defined function which is reflective of how medicinal chemists approach the similarity and complexity of chemotypes
5. Outcomes of space embeddings performed independently should be directly comparable both numerically and visually
6. Method must not be limited to capturing only linear relations
7. Ability to process reasonably large datasets (consisting of thousands of structures)
8. Ability to quantify distance between structures in the embedded space.

Existing chemical space embedding methods, to our knowledge, don’t meet all of the above criteria (see: Table S1, Supporting Information). However, most of these methods could be turned into one that meets almost all these criteria following a two-step procedure, as follows. First, a pre-embedding is generated with the help of a pre-defined set of “landscape” structures, e.g. Bemis-Murcko scaffolds (BMSs) [15]. Next, the most similar landscape structure (here: closest BMS based on SK-distance) is identified for each compound in the data set at hand. Then, each compound would assume the coordinates of the landscape structure identified as the most similar to a given compound. In section *“Pseudocode of the Scaffold t-SNE Method”* in SI we demonstrate how the original *t*-SNE method can be modified in accordance with these considerations. However, one of the most important criteria from the interpretation point of view is not met when using the above embedding strategy with existing methods. That is, the organizing principle of pre-embedding of landscape structures remains mostly hidden for the researcher. Moreover, the organizing principle is practically the result of certain optimization processes that largely depend on the input data at hand.

In this study, we aimed at constructing an embedding method that addresses this limitation so that it provides a simple, yet practical, embedding that can be interpreted intuitively by medicinal chemists and data analysts.

#### Method design

In the light of the above collected criteria, we devised a novel chemical space embedding method. The devised method was built on incorporating critical concepts introduced by prior art methods: use of landscape objects organized on a grid, use of embedding mechanism that is not influenced by the compound set to be embedded, and the ability to change resolution of the embedding [5, 9, 12].

The foundation of the novel method is provided by a family of so-called space filling curves, namely by Hilbert-Curves [16–18]. Provided that an ordering between data points, here BMSs, exists, with the help of Hilbert-Curve it is possible to embed the data points into a space of higher dimension, such as 2D, following an exact mathematical process. This embedding is a limit of embeddings resulted by utilizing so-called pseudo-Hilbert-Curves (PHCs) of increasing order. The order of the PHC can be thought of as the number of identical parts a unit of an area (or volume in higher dimensions) is divided into. The PHC of given order connects the middle points of these parts, and the number of identical parts can be derived from Eq. 2. The peculiar characteristics of PHCs is that increasing the order of the PHC the position of a given data point will converge to a limit in the higher dimension. In other words, the positions of data points are stabilized utilizing PHCs of increasing order in the embedded space. Considering that implementation exists for embedding PHCs, the question remained: How can one obtain a well-defined ordering of BMSs that is reflective of a medicinal chemist’s approach to this problem? Luckily, the Scaffold-Key (SK) algorithm addresses this exact question by providing a solution for the “intuitive” ordering of BMSs that was motivated by the analytical thinking of medicinal chemists [19]. For more information on the SK algorithm please refer to section “*Scaffold-Key Algorithm*”.

In the following section we provide the details of the structure embedding method that was designed with all the considerations detailed above.

#### Hilbert-Curve assisted structure embedding method

In order to define the chemical space of the Hilbert-Curve Assisted Structure Embedding (HCASE) method, a set of reference BMSs needs to be collected. The choice of reference BMS set depends on the context of scientific investigation. However, using a diverse set of BMSs or a collection of BMSs derived from compounds of a large bioactivity data set represent choices that can be adopted in a wide range of research settings. Note that compound structures that cannot be associated with a valid BMS structure are eliminated from the input set when generating the reference BMS set. Next, the SKs of reference BMSs are generated, and the BMSs are ordered according to their SK using alphanumeric ordering. In case of a tie, the InChI-Keys of BMSs are used to determine priority. In the arguably rare case when the InChI-Keys would be identical, then the “first” of such BMSs will gain priority. Of note, depending on the implementations of sorting algorithm, the choice of “first” BMS in a tie can be nondeterministic. Still, considering the low probability of such events, we consider the SK and InChI-Key based ordering practically deterministic.

Next, the reference BMS set is mapped on a line based on the rank of each BMS emerged from the SK-based ordering process. This line can be thought of a PHC which can be folded to a 2D space, or even higher dimensions following a well-known process [16]. The embedding of compounds with the help of such a line happens in a few steps.

First, the BMS of the compound at hand is extracted and the corresponding SK is generated. With the help of the SKs, the closest reference BMSs to the compound is identified. Next, the compound will assume the position of the closest reference BMS on the PHC. Finally, the PHC is mapped to a higher dimension space.

The process of mapping a PHC to higher dimension requires only two parameters as input: the order of the PHC and the number of dimensions. The latter was always set to 2D in this study, while the former was varied. Given the nature of PHCs, increasing the order of the PHC will lead to the stabilization of coordinates in the embedded space and to a more fine-grained embedding.

Reducing the algorithm to practice required us to consider two observations. First, the number of potential coordinates in the embedded space is a function of the order of PHC and the number of dimensions in the available implementation of PHC algorithm [20, 21].

In 2D, the PHC can be mapped on a *N* × *N* grid, where the value of *N* is given by *Eq. *2, whereas *z* denotes the order of the PHC. Accordingly, the *x* and *y* coordinates can take on values between *0* and *N − 1*, inclusive. Of note, we use the PHC-*z* notation in the text to distinguish PHCs of different order.

Second, the PHC emerged from the reference BMS set contains a finite set of data points, *i.e.,* BMSs. In the light of these limitations, it was necessary to introduce a binning-mechanism in order to mimic the behavior of PHCs.

2 $$N = 2^{z}$$

The binning-mechanism treats the number of potential coordinates (*|D|*) in the embedded space as the number of bins (see: Eq. 3, 4). Then, the bin-size *l* is determined based on the ratio of the size of the reference BMS set (*|S|*) and the number of bins minus one (see: Eq. 5). Note, that the correction term is necessary as the Hilbert-curve implementation uses zero-indexing, hence the minus one term.

Given a compound *i* and its closest reference BMS *S*_*i*_, the bin index *b*_*i*_ of the compound is computed by first dividing the SK-based rank of *S*_*i*_ by the bin-size, then rounding the resultant number to the nearest integer (see: Eq. 6). Of note, when setting the parameters of the algorithm, it should be considered that the limit of the resolution of the HCASE method is defined by the parameter combination where the number of potential coordinates exceeds the size of the reference BMS set.

3 $$D = \{ (x,y)\} \quad |\quad \forall x:x \in [0,N - 1],\quad \forall y:y \in [0,N - 1]$$

4 $$\left| D \right| = N^{2}$$

5 $$l = \frac{\left| S \right|}{{\left| D \right| - 1}}$$

6

**Fig. 3:**
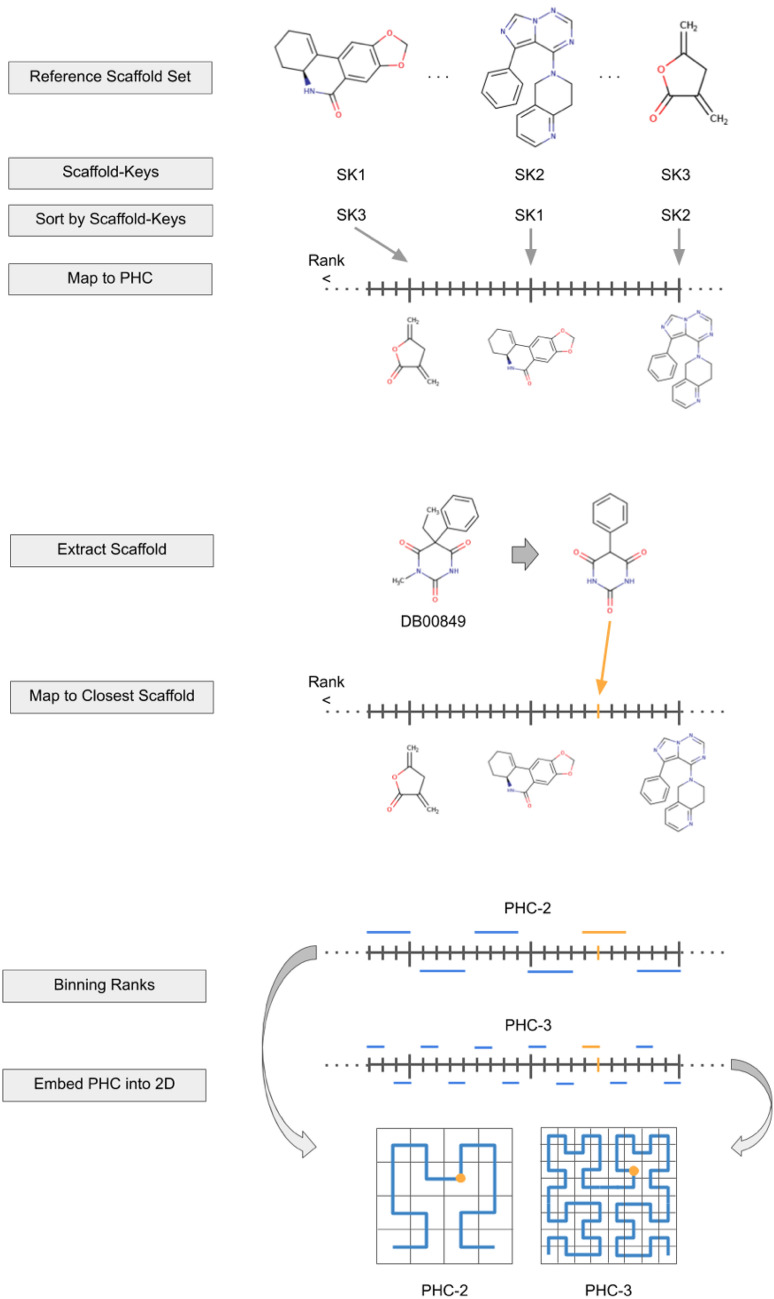
HCASE method. The process of embedding compounds into a chemical space with the HCASE method is demonstrated. The chemical space is defined by reference scaffolds which are ordered based on their Scaffold-Keys (SK). The HCASE method maps the reference scaffolds on a series of PHCs of increasing order. Then, a compound of the library to be embedded are mapped to its closest scaffold based on their Scaffold-Key distances (d_SK_). A binning step is also included in the process to make sure that each of the reference scaffolds, hence each compound, can be mapped to one of the possible coordinates in the higher dimension space. The number of possible coordinates is influenced by the order of the PHC the scaffolds are mapped to. A compound highlighted by yellow is tracked in this process. As it can be seen, the position of the compound in a 2D space is the function of the order of the PHC it was mapped to. Due to the nature of PHCs the position of compounds converges to a “stable” position when increasing the order of PHCs

Computing the bin indices of each compound gives rise to a mapping on a PHC which can be folded to 2*D* by defining parameter *z* [16, 20]. The main steps of the HCASE algorithm are visualized on Fig. 3.

### Pseudocode of the HCASE Method

The pseudocode of the HCASE method is provided below. Note that most of the functions highlighted with bold fonts represent well-known methods, therefore their pseudocode is not included. Such functions are: *generatePseudoHilbertCurve()*, *getHCCoordinates()*, *getScaffoldKey()* and *getBemisMurckoScaffold()*. The *binScaffolds()* and *getSKDistance()* functions are computed according to Eqs. 2, 3, 4, 5, 6 and Eq. 1, respectively.

Note that the lists in the pseudocode are zero-indexed. Furthermore, the elements of lists and tuples are also referenced according to array notation. Accordingly, the *D*[0][0] in the pseudocode reads: in the first item of list *D* (which is a tuple), the value of the first variable.

**Algorithm 1:**
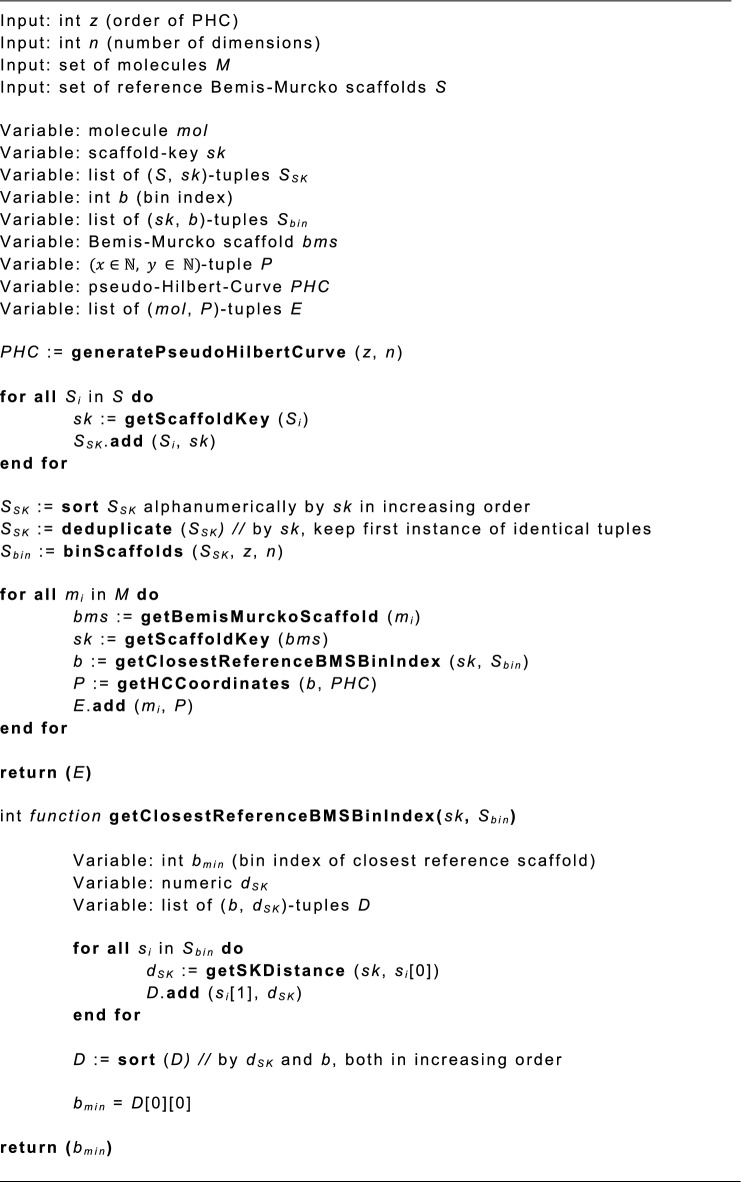
HCASE method

### General Cheminformatics Operations

Structures of substances were subject to the same standardization scheme unless otherwise stated. Standardization comprised of keeping only the largest compound of each substance and was performed in KNIME [24] with the help of CDK nodes [25–28]. Bemis-Murcko scaffolds (BMSs) [15] were generated for molecules using RDKit [29] cheminformatics suite and RDKit KNIME nodes [30]. Molecule structures were depicted with RDKit and ChemAxon’s Marvin Sketch [31]. Embeddings were only generated for compounds that could be associated with a BMS.

### k-nearest-neighbor analysis

Using the RDKit implementation of Morgan algorithm [29, 32], Morgan-fingerprint was generated for compounds with parameters of radius = 3 and fingerprint length = 2,048. The *k*-Nearest-Neighbors (KNNs) were identified for query compounds with the help of computing the Tanimoto-similarity coefficient [33, 34] of pairs of compounds. In this study the value of *k* was set to 5.

### Distance measure in embedded 2*D* space

The distance of compounds *i*, *j* mapped to a PHC can be quantified as the difference of the respective bin indices *b*_*i*_ and *b*_*j*_. This distance can be referred-to-as rank distance, i.e., d_r_ (see: Eq. 7).

7 $$d_{r} (i,j) = \left| {b_{i} - b_{j} } \right|$$

However, the idea of an intuitive embedding into *2D* suggests that structural proximity of compounds should be reflected in proximity of *2D* coordinates. Therefore, given the nature of the HCASE method, it is possible to define a perceived distance measure of the compounds in the embedded space as detailed below.

Compounds embedded in *2D* using the HCASE method are mapped to a latent grid. Each point of the grid represents a specific BMS or a group of BMSs, depending on the size of the reference BMS set and the parameter *z*. Therefore, the distance of two embedded compounds *i*, *j* “stretched” on this grid can be perceived as their Chebyshev-distance [35] (see: *Eq. *8). Of note, the Chebyshev-distance is a metric. However, since it is applied as a perceived distance measure, in this study we will refer to the Chebyshev-distance metric as Chebyshev-distance measure.

8 $$d_{C} (i,j) = \max_{n} \left| {i_{n} - j_{n} } \right|$$

### Quantifying space overlap similarity of different embeddings

Given an embedding generated by the HCASE method, one can compute the number of compounds associated with a reference BMS. More precisely, one need to count the number of compounds mapped to the bin the respective BMS was assigned to. In the function of *z* the number of bins is provided by $|D|$ (see: Eq. 4). This information can be condensed into an $|D|$ -dimensional *embedding-vector*. In such vector, the value of each dimension reflects the number of compounds associated with a specific bin, which bin is a point in the latent grid behind the embedding.

Quantifying the similarity of two embedding-vectors **A** and **B** can be performed in analogous manner to computing the similarity of two molecular count-fingerprints [36] with the help of a modified Tanimoto-similarity coefficient (see: *Eq. *9) [33, 34, 37, 39].

9 $$\theta _{{A,B}} = \frac{{\sum\nolimits_{{i = 1}}^{{\left| D \right|}} {{\mathbf{A}}_{i} {\mathbf{B}}_{i} } }}{{\sum\nolimits_{{i = 1}}^{{\left| D \right|}} {{\mathbf{A}}_{i}^{2} } + \sum\nolimits_{{i = 1}}^{{\left| D \right|}} {{\mathbf{B}}_{i}^{2} - \sum\nolimits_{{i = 1}}^{{\left| D \right|}} {{\mathbf{A}}_{i} {\mathbf{B}}_{i} } } }}$$

### Input data

#### Compound libraries

Compound libraries were collected from two sources: approved drugs of DrugBank database (version: 2.0.9) [40], and the CANVASS library [41]. These libraries are comprised of 2,073 and 344 compounds, respectively.

#### ChEMBL scaffolds

A set of unique BMSs of size 63,783 has been extracted from ChEMBL database (version: 24.1) [42] using the same procedure and KNIME workflow [43] that was used to derive the knowledge base of SmartGraph platform [44]. This set was derived from the set of all unique BMSs included in ChEMBL database based on the number of compounds they are associated with. That is, only BMSs were selected if they are connected to less than 100 and at least 5 unique compounds. Out of 63,783 scaffolds, after processing by RDKit and deduplication by SKs, we identified 55,961 unique BMSs.

#### Natural products scaffolds

A set of natural products were extracted from the ChEMBL database (version: 23) consisting of 1,921 compounds [41]. BMSs of these compounds were identified and their SKs were generated. Subsequently, the BMSs were deduplicate based on the SKs, which resulted in a set of 546 scaffolds (NatProd scaffolds).

For the sake of reproducibility of the experiments, all source code and data used to perform the experiments are publicly available in the source-code repository: https://github.com/ncats/hcase [23].

#### Cherry-Picked scaffold set

In some of the experiments we sought to monitor the position of certain scaffolds as a result of the embedding process. To this end, a subset of ChEMBL scaffolds (see above) was manually cherry-picked in a way so that their ranks are separated by larger and smaller intervals. The 9 cherry-picked BMSs are shown in Table 1 and Fig. S3 in Supporting Information (SI). Additionally, the immediate 50 SK-ordering based nearest neighbors (in both directions) were also included into this set. The resultant set, therefore, consists of 9 manually selected BMSs and 100 SK-ordering based nearest neighbors of each. This set is referred to as "cherry-picked scaffold set" throughout the text, and it consist of 909 scaffolds in total.

**Table 1:** Cherry-picked BMSs of the ChEMBL Reference Scaffold Set

Cherry-picked reference scaffold rank	Color
5000	Blue
15,000	Orange
16,000	Green
25,000	Red
26,000	Purple
35,000	Brown
44,000	Pink
45,000	Gray
55,000	Yellow-green

Due to the separation of the 9 manually selected BMSs based on their SK-ordering rank, there is no overlapping scaffold between the neighbors of the 9 BMSs. The 100 SK-ordering based nearest neighbors are marked with the corresponding color of one of the 9 manually selected BMSs throughout the text and SI.

#### Reduced scaffold set

In some of the experiments we utilized a subset of the ChEMBL scaffolds (see above). The subset was generated in two steps. First, we selected scaffolds randomly from the ChEMBL scaffolds so that the size of this set was 90% of that of the original set. Second, the union of this set and the cherry-picked scaffold set (see above) was created. The purpose of this step was to assure consistency across experiments aimed to investigate the relationship between the utilized space embedding method, the underlying reference scaffold set, and the positions of the embedded cherry-picked scaffolds in the map.

The resultant set is referred to as the "reduced scaffold set" throughout the text and SI.

## Results and discussion

### Clustering of scaffolds mapped on a Hilbert-curve

We sought to monitor the position of certain scaffolds as a result of the embedding process. Our expectation was that scaffolds that exhibit similarity in terms of chemical structure and complexity should be placed closely in the embedded space with the help of PHCs. Unlike real numbers, scaffolds cannot be mapped to a line in a linear fashion, as their “absolute value” cannot be readily determined. Instead, we used SKs to derive a relative ordering of BMSs. Employing the established relative ordering we were able to map BMSs onto a line, in this case onto a PHC. This mapping provides the basis of embedding the BMSs into a *2D* space by “folding” the PHC into *2D*. The folding of the PHC is determined by the order of the PHC, i.e., parameter *z.* This parameter was varied in an interval, determined by the number of BMSs at hand. Therefore, for a given set of BMS we generated a series of embeddings resulted by utilizing PHCs of increasing order (parameter *z*). The effect of increasing *z* is that the BMSs are embedded into *2D* space according to an increasingly complex folding pattern. This can be interpreted as increasing the resolution of the embedding.

First, the ChEMBL reference BMSs were ordered according to their SKs.

The maximal order of PHC to be used was determined by the size of the ChEMBL reference scaffold set. A PHC of *z* = 8 gives rise to a space that is defined by a latent grid of 65,536 points (see: Eqs. 2, 3, 4). The size of ChEMBL reference scaffold set (55,961) is less than this value but is larger than the number of potential coordinate pairs in a space defined by a PHC of *z* = 7. Taken these in consideration, the order of PHCs employed in this investigation was varied in the range of *z* = [2, 8].

**Fig. 4:**
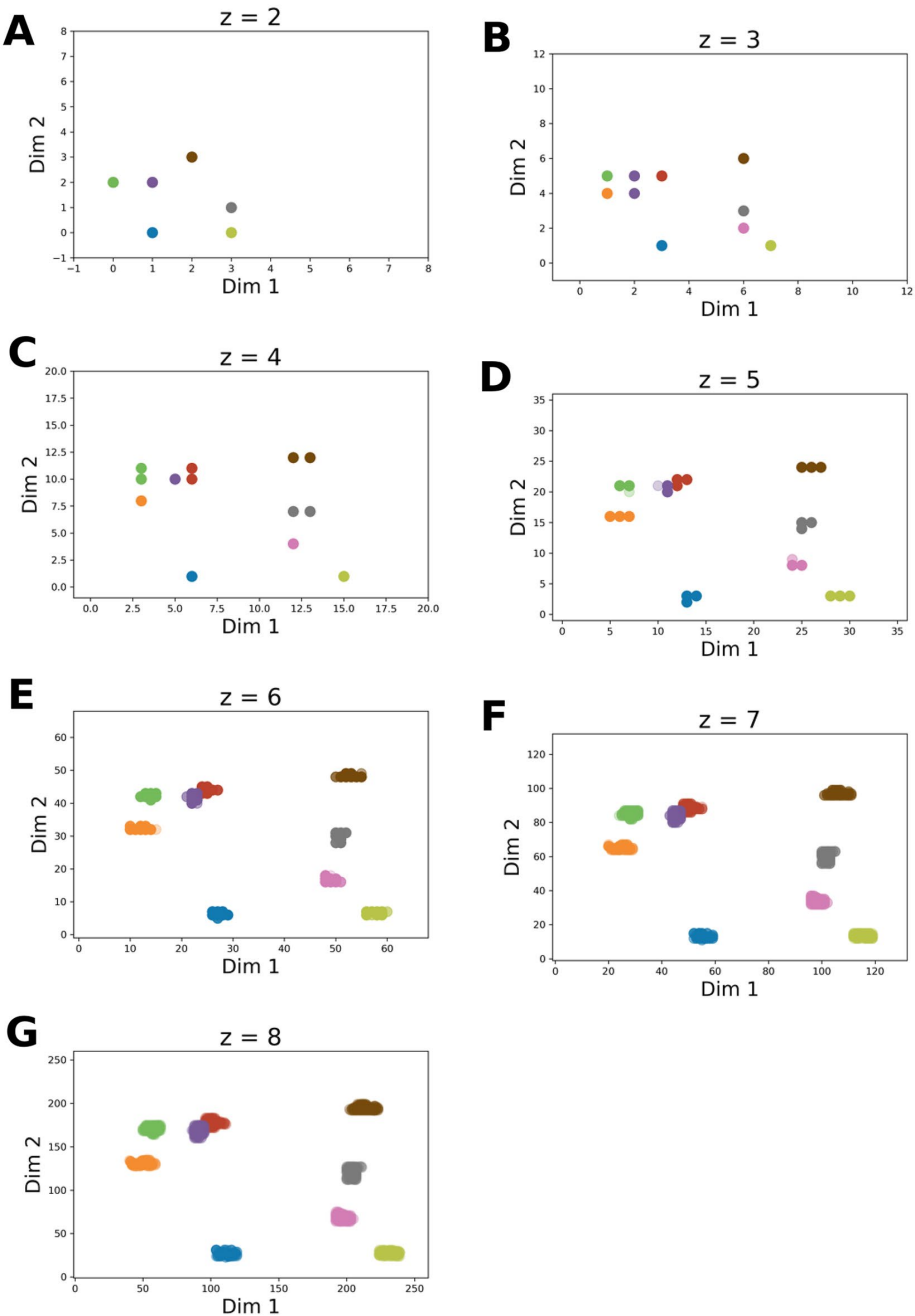
Tracking the position of the cherry-picked scaffold set on the PHCs in the ChEMBL reference scaffold space. ChEMBL scaffolds were mapped onto PHCs of varying order (value of z was incremented in the range of [2, 8] for subfigures a-g, respectively). The order of the PHC is indicated by the suffix in the title of the subfigures. On each PHC we tracked the positions of the BMSs in the cherry-picked scaffold set. The cherry-picked scaffolds and their and respective colors are provided in Table 1. The color of the SK-ordering based nearest neighbors is the same as that of the corresponding cherry-picked scaffold

As it was described in section “*Hilbert-Curve Assisted Structure Embedding Method*”, the reference scaffolds are assigned to bins in the function of *z*. Consequently, low values of *z* give rise to a low-resolution latent grid, where many of the marked scaffolds are assigned only to a few grid points, as expected (see: Fig. 4a–c). Increasing the value of *z*, i.e., the resolution of embedding, it can be seen that the marked BMSs start to separate, giving rise to clusters, i.e. groups of closely-binned BMSs (see: Fig. 4d–g).

Based on the results of the embedding, it can be seen that the HCASE method is able to produce clusters of varying granularities in the function of parameter *z*. This feature therefore provide opportunity to control the resolution of the embedding depending on the use case at hand. Furthermore, the positions of clusters are the function of the bin indices of their underlying BMSs. The stabilization property of PHCs is demonstrated by the results, as the position of individual scaffolds converges to a point in 2D space as the resolution of the embedding (parameter *z*) is increased.

These findings support, that using the HCASE method, it is possible to develop an intuition for associating type of scaffolds, or group of scaffolds with various segments of the embedded *2D* space. Therefore, we concluded that the properties of latent grid generated by HCASE method are adequate to serve as the basis for compound embedding.

### Embedding of KNNs

Building on the promising results described in the previous section, we sought to analyze the embedding of a compound library with the help of ChEMBL reference scaffold set and the HCASE method. To this end, the embedding of the DrugBank data set was performed. The range of *z* values were identical to the range utilized in the previous section, considering that we used the same reference scaffold set, i.e., ChEMBL. To demonstrate the embedding process, we selected 5 molecules randomly from the DrugBank dataset and the *k* = 5 nearest neighbors of each was determined as described in section “*K-Nearest-Neighbor Analysis*”. This gave rise to a unique set of 30 compounds. The list of query compounds, their NNs and the values of Tanimoto-similarity coefficients are provided in Table 2 and Fig. 1 in decreasing order of similarity.

**Table 2:** *K* = 5 nearest neighbors of 5 randomly selected drug molecules

*C*_*query*_	*C*_*NN*_	Rank	*T*_*sim*_
DB00006	DB04931	1	0.44444
DB00006	DB01284	2	0.40520
DB00006	DB00050	3	0.39316
DB00006	DB09067	4	0.38214
DB00006	DB06825	5	0.36975
DB00849	DB01174	1	0.48980
DB00849	DB00794	2	0.44231
DB00849	DB05246	3	0.32143
DB00849	DB01437	4	0.31667
DB00849	DB00252	5	0.27778
DB00977	DB01357	1	0.69863
DB00977	DB04575	2	0.62963
DB00977	DB00655	3	0.50649
DB00977	DB00783	4	0.50649
DB00977	DB04573	5	0.50000
DB01362	DB01249	1	0.88235
DB01362	DB09135	2	0.85714
DB01362	DB09134	3	0.61667
DB01362	DB09313	4	0.27174
DB01362	DB01578	5	0.21978
DB04837	DB11609	1	0.35000
DB04837	DB00257	2	0.30882
DB04837	DB00333	3	0.27273
DB04837	DB01231	4	0.26471
DB04837	DB08944	5	0.26154

Fingerprint: Morgan (radius = 3, length = 2048). *C*_*query*_: query compounds, *C*_*NN*_: nearest neighbors of query compounds based on their Morgan fingerprint and Tanimoto-similarity (*T*_*sim*_)

**Fig. 5:**
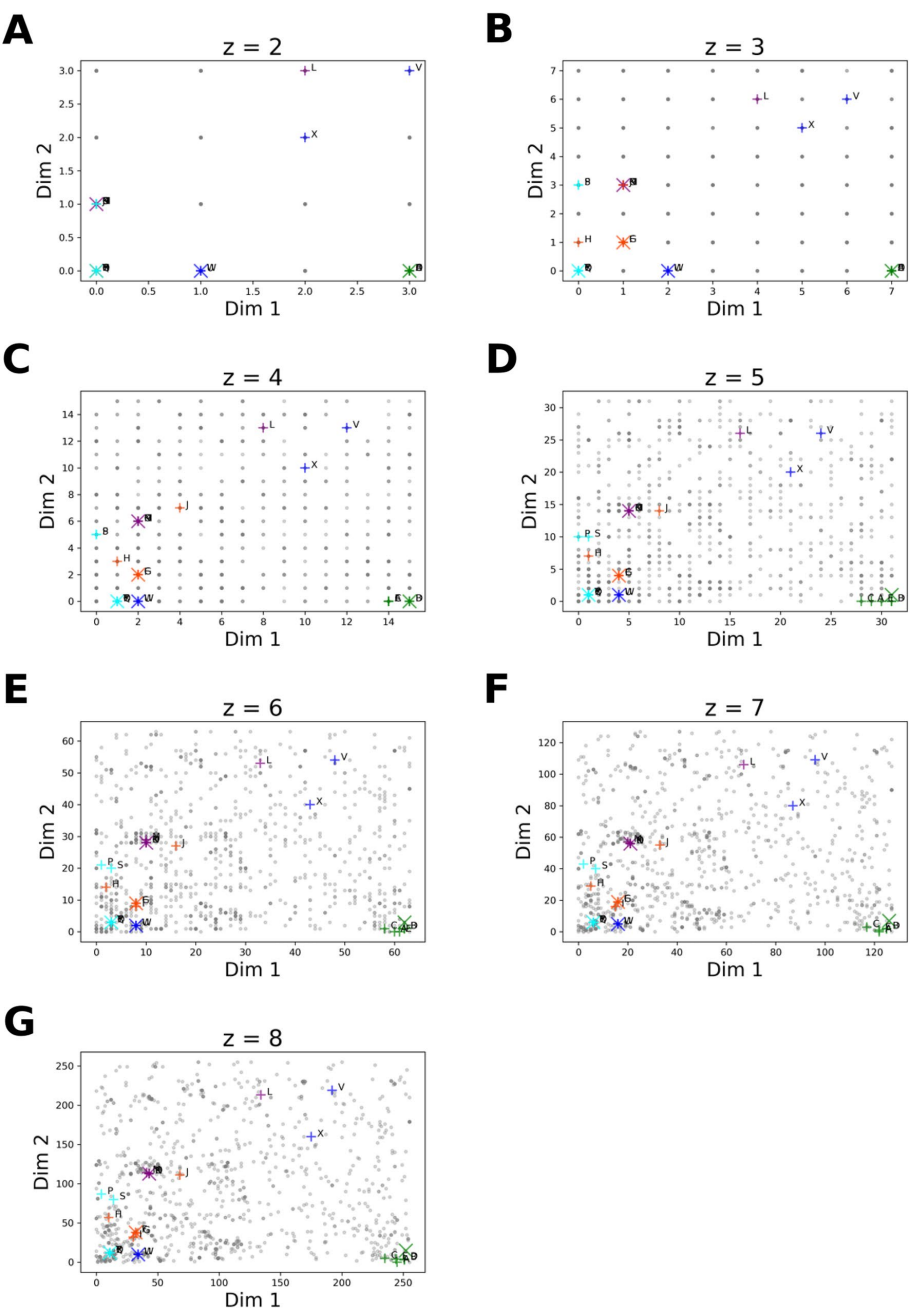
HCASE embedding of drug compounds into ChEMBL scaffold space. Shown is the HCASE embedding of *k* = 5 nearest neighbors of 5 randomly selected compounds from the DrugBank dataset. The order of PHC utilized for structure embedding is indicated by suffix in the titles of the subfigures. Enlarged (X) signs indicate the query compound of KNN analysis; green: DB00006, orange: DB00849, purple: DB00977, aqua: DB01362, blue: DB04837. ( +) signs indicate the NNs of a query compound with identical color. Gray circles indicate other DrugBank compounds. Compounds are labeled according to Fig. 1

Considering all data points, it can be seen in Fig. 5 that the positions of individual datapoints are stabilized with increasing order of the underlying PHC. Also, increasing values of *z* give rise to a finer-grained clustering of data points.

Regarding the KNNs, most of them are clustered closely to the query molecules, as expected, but some of them are placed further away. For instance, at *z* = 8 we can make the following observations. In the case of query molecule DB04837, i.e., “blue” series, two of the NNs (“X”, “V”) are positioned farther from DB04837, which is explained by the more complex BMS present in those two NNs as compared to the rest of the series. Interestingly, the fifth NN (“Y”) in the same series is co-positioned with the query compound DB01362 (color: aqua), but it can’t be seen due to overlap of markers. The reason for this is that “Y” and DB01362 share the same BMS, i.e., the benzene ring. Consequently, they were mapped to the same reference scaffold hence positioned to the same coordinate in the embedded space.

Similar trends can be observed in the other NN series as well. Typically, when the BMSs of NNs differ in exocyclic groups, then they are embedded still relatively closely. However, when the BMSs differ by extra rings, then they will be placed further away. This phenomenon can be explained by the ordering of scaffolds based on their SKs. These observations argue that the embedding results in clustering that matches closely the mindset of a medicinal chemists when analyzing chemotypes. For example, in the case of the “purple” series (query molecule: DB00977) most of the NNs in the series share the same or very similar BMS, except compound “L”, whose BMS is more complex than that of other NNs, hence it is positioned further away from other members of the series. The peculiarity of this fact is more obvious when one considers the Tanimoto-similarity of the NNs to the query molecule in the “purple” series; compound “L” is the second NN of the query compound, still it is positioned the furthest from other compounds of the series. Separation of compound “L” from the rest of the series members would be considered correct from a medicinal chemist’s view, as compound “L” has the most dissimilar BMS in that series compared to the other BMSs.

### Embedding of randomly selected compounds

In order to contrast the above findings, we selected 25 random molecules from the DrugBank dataset (see: *Fig. S4* in SI) and compared their embedding with that of the NN series. In *Fig. S5* in SI, the embedding of these 25 compounds is shown besides the embedding of the 5 query molecules of the previous experiment. As it can be seen, the embedding of the random set exhibits a reduced level of clustering as compared to the case of the NN series. While some clustering is present in this set, mainly contributed to the presence of benzene ring as the BMS in several compounds, the overall picture resembles a random distribution of the embedded coordinates.

In summary, the above findings demonstrate that it is possible with the HCASE method to embed compounds in a chemical space that is able to differentiate molecules based on chemotypes, and to provide a logical and intuitive arrangement of these chemotypes. Therefore, it can be argued that clustering emerging in the embedded space will be reflective of a medicinal chemist’s analytical thinking.

### Comparison of the results of different embedding outcomes

After concluding the HCASE method is able to generate intuitive embedding of a chemical library we intended to analyze how we can compare the outcome of different embeddings. This first required to investigate the effect of utilizing different scaffold reference sets, then to quantify how well different embedding results are aligned with each other.

To this end, we performed separately the embedding of the DrugBank and CANVASS libraries utilizing two different reference scaffold sets: ChEMBL and NatProd. As explained in section “*Clustering of Scaffolds Mapped on a Hilbert-Curve*” the upper limit of *z* depends on the size of the reference scaffold set at hand. We determined that this upper limit is *z* = 8 in case of the ChEMBL set. The NatProd scaffold reference set is comprised of 546 BMSs, hence the upper limit of *z* is 5.

#### Qualitative comparison

**Fig. 6:**
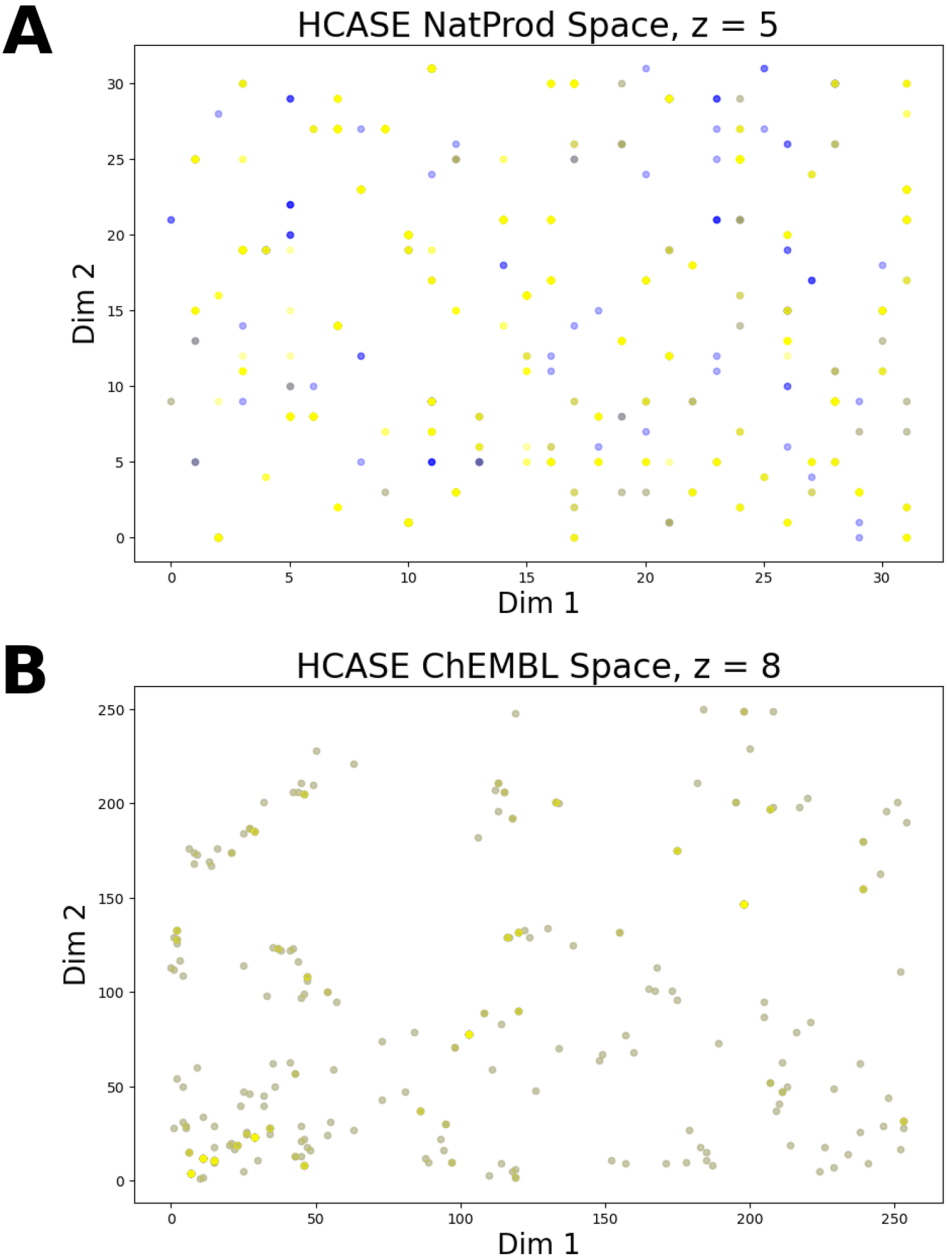
Comparison of the HCASE embeddings of compounds in Natural Product and ChEMBL scaffold space. Blue: CANVASS compounds, yellow: drugs. Overlapping datapoints are colored by green–brown color due to the transparency of the datapoints. **A)** NatProd Scaffold Space, PHC-5 ($$z=5$$). **B** ChEMBL Scaffold Space, PHC-8 ($$z=8)$$

First, let us consider the embeddings in the NatProd chemical space as shown in *Fig. S6* in SI. The positions of compounds of both libraries are also distributed across all possible 16 coordinates at *z* = 2. At *z* = 3 the CANVASS compounds are assigned to only 59 coordinate pairs, whereas in the case of DrugBank library to 61 (see: Fig. 6a).

In the case of the ChEMBL chemical space (see: *Fig. S7* in SI) at *z* = 2, the coordinates associated with the embedded compounds of both libraries are distributed across all potential 16 coordinates. At *z* = 3, in the case of the CANVASS library, the compounds are only assigned to 43 different coordinates. However, the compounds of DrugBank dataset are assigned to all potential coordinates. At higher values of *z*, the overlap of the respective pairs of embeddings becomes less and less pronounced, i.e., the two dataset start to separate, as in the previous case (see: Fig. 6b).

Based on the qualitative comparison, it can be observed that the DrugBank dataset occupies larger portion of the embedded space. This is not surprising considering that CANVASS is a smaller library, and a less diverse one. Nevertheless, the overlap of the two libraries seems to be larger in the NatProd space. As seen at *z* = 4 the CANVASS library is more spread-out in this space. Since this space is defined by scaffolds extracted from natural products, the CANVASS library indeed seems like a good representative of the natural product space. However, the drug molecules represent structures with BMSs that even better represent the underlying NatProd reference scaffold set. Considering that many drug molecules are natural product derivatives, and the presence of larger diversity in the DrugBank vs. the CANVASS library, the fair amount overlap in this space of the two libraries can be considered reasonable.

In the ChEMBL chemical space both libraries show clustering which becomes prominent at *z* > 5 values, although the clustering is more obvious in the case of CANVASS library. Drug molecules represent this chemical space also to a reasonable degree, whereas the CANVASS molecules form “islands”. These islands are mostly overlapping with members of the DrugBank library. Further, in this chemical space the unoccupied area is visible to a larger extent as compared to the NatProd space.

Based on the above findings, we concluded that the choice of the reference scaffold set influences the embedding in two major manners. First, the reference scaffold set serves as a perspective which the structural similarities are analyzed from. Accordingly, the embedding of CANVASS and DrugBank libraries paint a more similar picture in the NatProd space than in ChEMBL space. Second, the separation of structures can be promoted by the choice of the reference scaffold set.

#### Quantitative comparison

In the previous section we investigated how the embeddings of two chemical libraries can be compared qualitatively. However, there can be cases when one might want to quantify the overlap (similarity) of two embeddings.

**Fig. 7:**
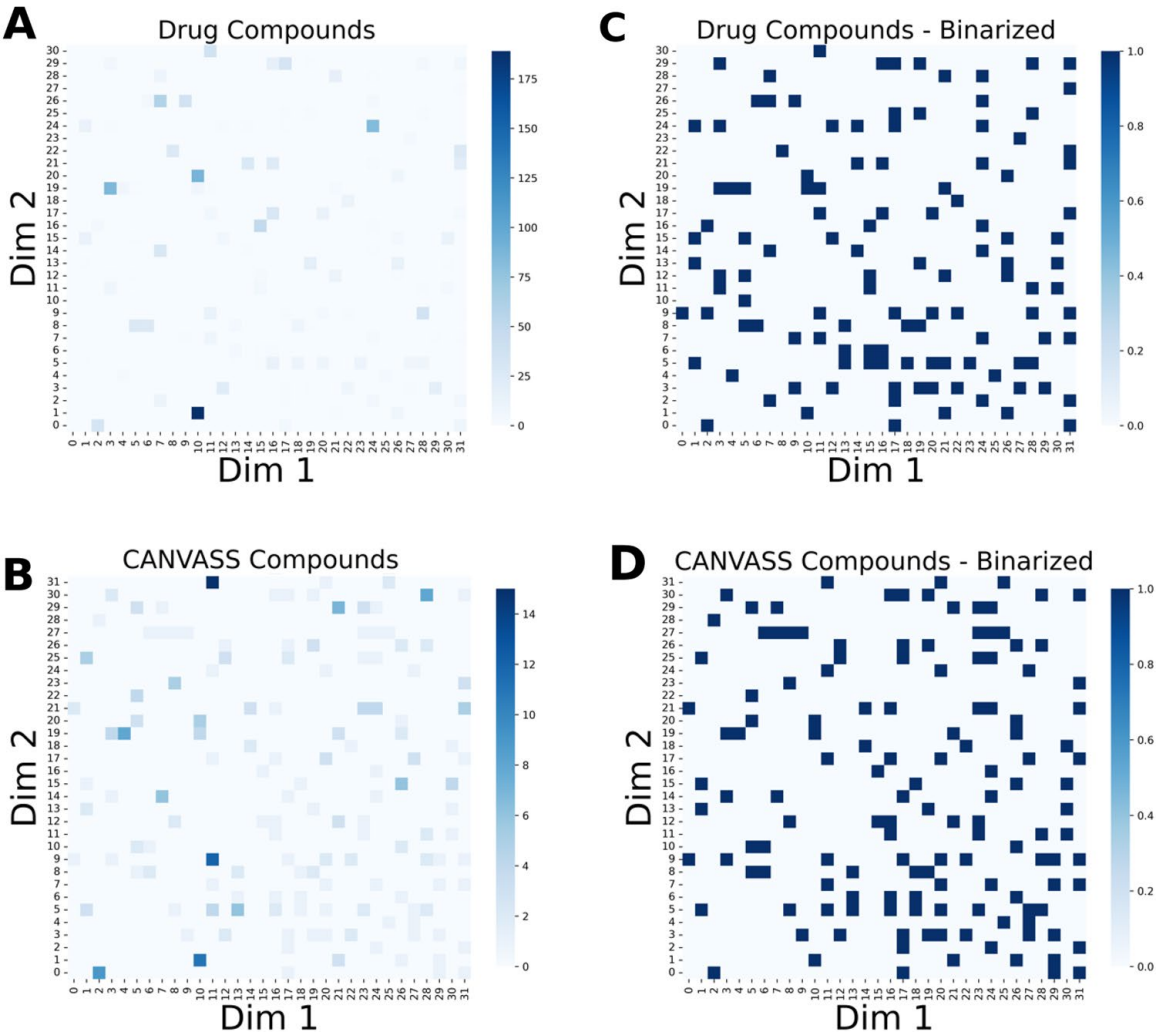
Distribution of compounds in the map obtained by HCASE embedding. Compounds were embedded into the NatProd scaffold space with the help of HCASE method. The intensity of each cell of the heatmaps is proportional to the number of compounds assigned to each cell, i.e., position in the embedded space. **A** Aggregated number of drug compounds embedded into HCASE NatProd space. **B** Aggregated number of CANVASS compounds embedded into HCASE NatProd space. **C** Aggregated number of drug compounds embedded into HCASE NatProd space, binarized. **D** Aggregated number of CANVASS compounds embedded into HCASE NatProd space, binarized

To this end, one of the natural solutions is provided by aggregating the number of compounds associated with each given point in a 2D coordinate system. In the case of the HCASE method we can rely on only integers as coordinate values. The aggregated values can be condensed to a heatmap, in which cells correspond to specific coordinates in the embedded space. The color of each cell is the function of the number of molecules assigned to the respective coordinate. This solution is shown in Fig. 7a and 7b, which reflect the aggregated results of embedding the DrugBank and CANVASS libraries in the NatProd chemical space with the HCASE method at *z* = 5, respectively. The heatmap provide an intuitive way to quickly see which regions of the same chemical space are covered by either of the libraries.

In a more qualitative approach, it is also possible to present the coverage of the chemical space in a binary way. That is, the value of a point in the 2D HCASE space is 1, if at least one compound is mapped to that point. Otherwise, the value of the point is 0. This information can also be represented as a heatmap as shown in Fig. 7c and 7d.

Beyond the graphical solution, it also possible to quantify the overlap of the embedding of two libraries by using a measure (*θ*) analogous to the Tanimoto- similarity coefficient of count-fingerprints, as described in section “*Distance Measure in Embedded 2D Space*”. The results of quantifying the overlap of two libraries based on *θ* is provided in Table 3. The results confirm the qualitative observations that the overlap of the two datasets decreases with increasing values of *z*, *i.e.*, by increasing the resolution of the embedding. At the highest resolution, the overlap is greater in the NatProd space than in the ChEMBL space, just as it was observed in the qualitative analysis. While the values of *θ* are quite small in most cases, still, it can be used to quantify the extent of overlap.

**Table 3:** Space overlap between DrugBank and CANVASS libraries in different chemical spaces

*Reference Scaffold Set*	*z*	*θ*
ChEMBL	2	0.2042
ChEMBL	3	0.1609
ChEMBL	4	0.1138
ChEMBL	5	0.0864
ChEMBL	6	0.0593
ChEMBL	7	0.0525
ChEMBL	8	0.0519
NatProd	2	0.1836
NatProd	3	0.1041
NatProd	4	0.0785
NatProd	5	0.0758

z: order of the PHC, θ: overlap

### Perceived distance in the embedded 2*D* space

The promise of utilizing a PHC for chemical space embedding is that the objects mapped to close proximity on the curve will also be embedded in the higher dimensional space in close proximity. Therefore, we sought to explore whether those distance values translate in the embedded *2D* space in a way that can be perceived as distance measure.

Considering that the reference scaffolds create a latent grid behind the embedded space, it seemed natural to investigate the relation between the rank-distances (*d*_*r*_) of compounds and the Chebyshev-distances (*d*_*C*_) of embedded coordinates (see: section “*Distance Measure in Embedded 2D Space*”).

To this end, we first investigated the Pearson-correlation [47] of the two different types of distance measures with the help of the DrugBank and CANVASS compound libraries embedded both in ChEMBL and NatProd chemical spaces. First, the correlation was determined by taking into account all compounds per dataset. Results are shown in Table 4. It can be seen that there is a reasonable level of correlation between *d*_*r*_ and *d*_*C*_. The highest correlation was found to be 0.73 and 0.72 for the DrugBank and CANVASS datasets, respectively, when using the ChEMBL reference scaffold set.

**Table 4:** Pearson-correlation of Chebyshev–distances and SK–rank distances

Dataset	Reference Scaffold Set	*z*	Correlation
DrugBank	ChEMBL	2	0.6974
DrugBank	ChEMBL	3	0.7195
DrugBank	ChEMBL	4	0.7186
DrugBank	ChEMBL	5	0.7278
DrugBank	ChEMBL	6	0.7299
DrugBank	ChEMBL	7	0.7297
DrugBank	ChEMBL	8	0.7292
DrugBank	NatProd	2	0.5797
DrugBank	NatProd	3	0.5569
DrugBank	NatProd	4	0.5772
DrugBank	NatProd	5	0.5740
CANVASS	ChEMBL	2	0.6458
CANVASS	ChEMBL	3	0.7157
CANVASS	ChEMBL	4	0.7162
CANVASS	ChEMBL	5	0.7175
CANVASS	ChEMBL	6	0.7217
CANVASS	ChEMBL	7	0.7210
CANVASS	ChEMBL	8	0.7201
CANVASS	NatProd	2	0.5586
CANVASS	NatProd	3	0.5044
CANVASS	NatProd	4	0.5343
CANVASS	NatProd	5	0.5337

*z*: Order of the PHC

Interestingly, in the case of NatProd reference scaffold set the correlation values were lower as compared to other data series, observed in the range of [0.50, 0.58]. This might be an indication that the underlying latent grid has limited capacity to distinguish between chemotypes.

Furthermore, the highest correlation values were not observed at the highest value of *z*. This might indicate that the resolution associated with the highest *z* value might not be the “ideal” one in the light of the reference scaffolds and the compound set at hand. A more in-depth analysis of this phenomenon is beyond the scope of this study.

To further support these finding, we generated non-overlapping sets of randomly selected compounds from the DrugBunk dataset. Each set was comprised of 100 compounds. The mean and standard deviation of the correlation between the two distance measures is provided in Table 5. It can be seen that the observed correlations are well aligned with those obtained from considering the positions of all compounds in a given embedding.

**Table 5:** Pearson-correlation of chebyshev-distances and SK-rank distances in embedded subsets of DrugBank dataset

Reference Scaffold Set	*z*	Correlation—Mean	Correlation—Std
ChEMBL	2	0.7015	0.0278
ChEMBL	3	0.7233	0.0310
ChEMBL	4	0.7224	0.0323
ChEMBL	5	0.7315	0.0295
ChEMBL	6	0.7334	0.0293
ChEMBL	7	0.7331	0.0294
ChEMBL	8	0.7325	0.0293
NatProd	2	0.5832	0.0541
NatProd	3	0.5599	0.0540
NatProd	4	0.5799	0.0495
NatProd	5	0.5771	0.0503

In addition, we performed the identical analyses on the entire set and subsets of the same embeddings but using the Kendall-correlation [48] instead of the Pearson-correlation. The results paint a similar picture (see: Tables. S2–S3). That is, we observe acceptable correlation between the Chebyshev-distance and rank distance values, albeit typically of modestly lower values than their Pearson-correlation counterparts. Notably, the Kendall-correlation values tend to favor lower *z*-values, but the differences observed at the lowest and other z-values do not reflect qualitative differences. Overall, we concluded that Kendall-correlation values, similarly to Pearson-correlation values are indicative of acceptable correlation between the rank and Chebyshev-distances.

In order to investigate the relationship between the position of compounds on the unfolded pseudo Hilbert-curve and their 2D coordinates, we performed further analyses as described in details in sections “Distance Rank Correlation Analysis” and “Detection of Canyons on the 2D Maps”, in SI [46]. Based on the results of these analyses we concluded that the distances between compounds on 2D maps generated by HCASE method are reasonably reflective of their close or distant placement on the unfolded pseudo Hilbert-curve (see: Fig. S23, in SI). Furthermore, we investigated the emergence of anomalies (“canyons”) on 2D maps, where points in close proximity in 2D space are relatively distantly placed in the underlying pseudo Hilbert-curve. We concluded that the extent of these canyons introduced by the HCASE method in terms of coverage on the 2D maps is reasonably low, hence acceptable (see: Fig. S24, in SI).

In summary, there is an acceptable correlation between the two distance measures *d*_*r*_ and *d*_*C*_. Therefore, we propose that Chebyshev-distance measure can be considered as a perceived distance measure to quantify distances in the embedded 2D space generated by the HCASE method.

### Comparison of HCASE method with prior art

In the final set of experiments, we set forth to compare the HCASE method with prior art. Considering the broad use of *t*-SNE algorithm and its premise to preserve neighborhood information of objects in the embedded space, we decided to use this method for comparison. The comparison of the two methods involved three scenarios.

In the first scenario, we investigated the clustering property of the two embedding methods. Clustering is important, because medicinal chemist would expect similar structures to be positioned closely in a map, whereas dissimilar ones further away.

**Fig. 8:**
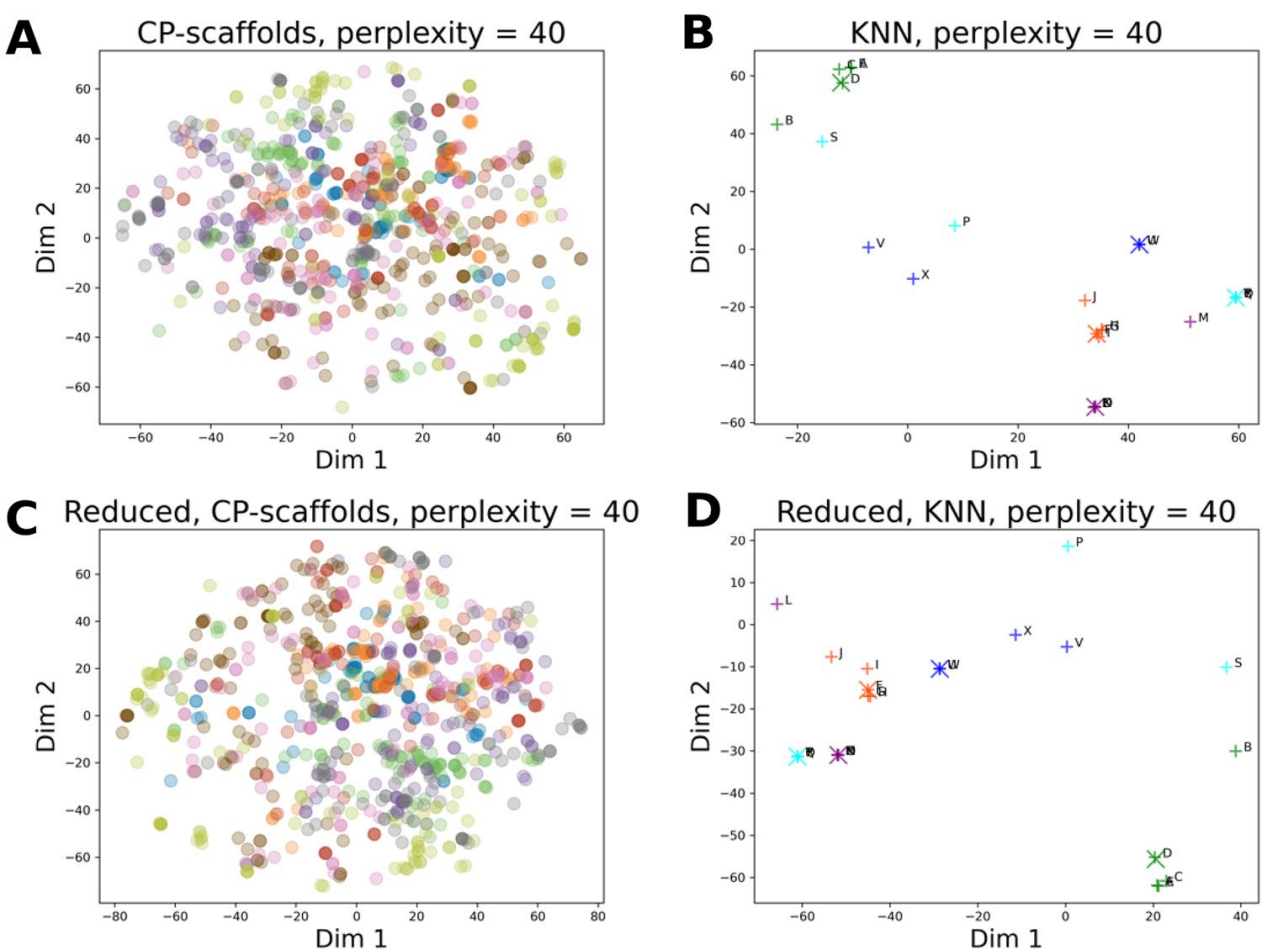
Cherry-picked scaffold set and drug molecules in *t*-SNE chemical spaces. The parameters of *t*-SNE embedding were set to default values, except for perplexity, i.e., learning rate = 200, iteration number 1000. **A** ChEMBL *t*-SNE space defined by the *t*-SNE embedding of ChEMBL scaffolds at perplexity = 40. Highlighted are the BMSs in the cherry-picked scaffold set. **B** Scaffold *t*-SNE embedding of *k* = 5 nearest neighbors of selected DrugBank molecules into ChEMBL *t*-SNE space. **C** Reduced scaffold *t*-SNE space defined by the *t*-SNE embedding of the reduced scaffold set. Highlighted are the BMSs in the cherry-picked scaffold set. **D** Scaffold *t*-SNE embedding of *k* = 5 nearest neighbors of selected DrugBank molecules into reduced scaffold *t*-SNE space. The cherry-picked scaffold set is colored according to colors provided in Table 1. The colors of the cherry-picked scaffolds were used to indicate their respective 100 SK-ordering based nearest neighbors. Enlarged (X) signs in *Fig. 8B and 8D* indicate the query compound of KNN analysis; green: DB00006, orange: DB00849, purple: DB00977, aqua: DB01362, blue: DB04837. ( +) signs indicate the NNs of a query compound with identical color. Compounds are labeled according to Fig. 1

**Fig. 9:**
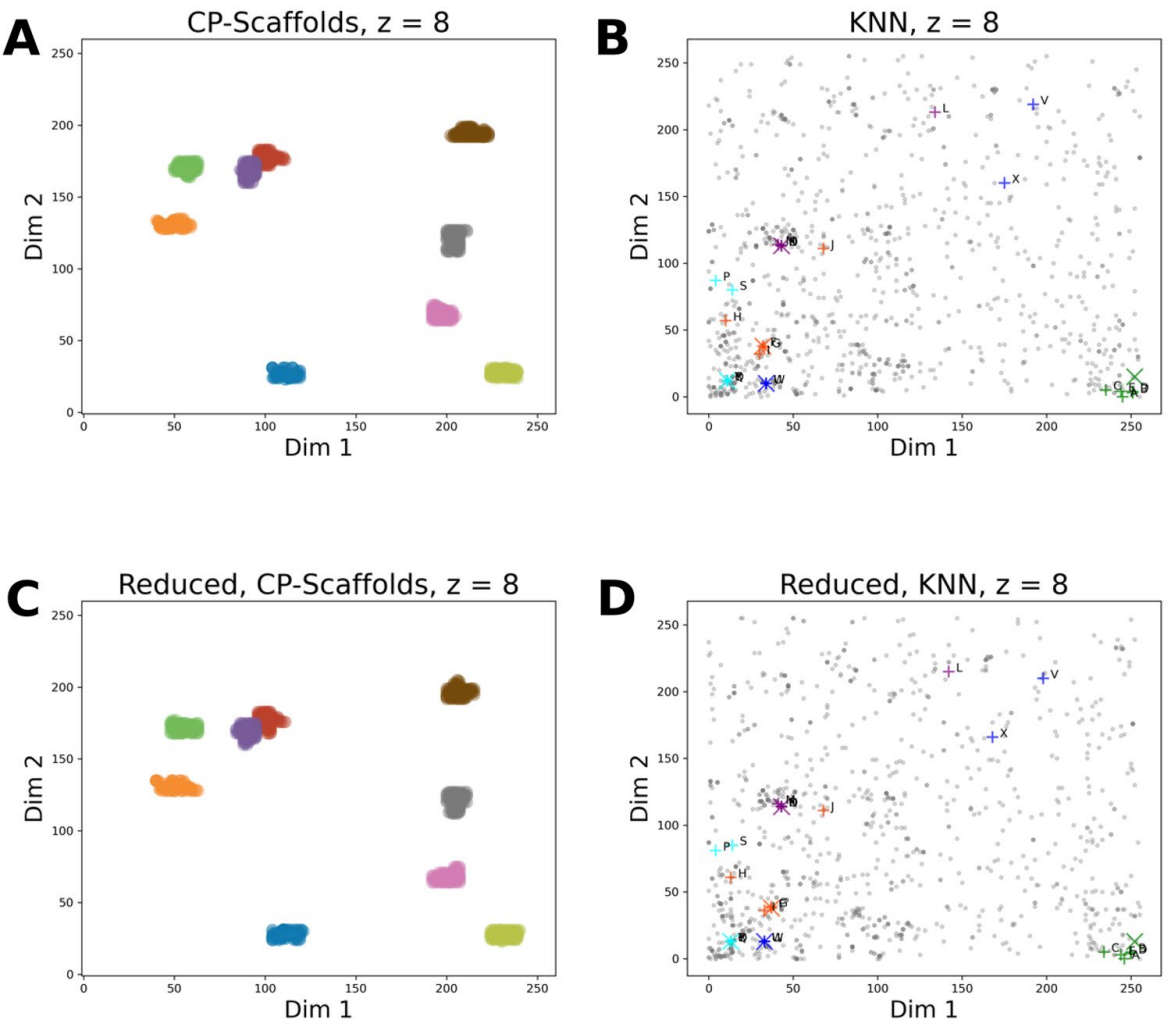
Cherry-picked scaffold set and drug molecules in HCASE chemical spaces. **A** ChEMBL scaffolds were mapped onto a PHC of $z=8$. Positions of BMS belonging to the cherry-picked scaffold set are highlighted on the PHC. **B** Embedding of *k* = 5 Nearest Neighbors of selected DrugBank Molecules with HCASE into ChEMBL space employing an PHC of $z=8$. **C** The reduced scaffold set was mapped onto a PHC of $z=8$. Positions of BMS belonging to the cherry-picked scaffold set are highlighted on the PHC. **D** Embedding of *k* = 5 Nearest Neighbors of selected DrugBank Molecules with HCASE into reduced scaffold set space employing an PHC of $z=8$. The cherry-picked scaffold set is colored according to colors provided in Table 1. The colors of the cherry-picked scaffolds were used to indicate their respective 100 SK-ordering based nearest neighbors. Enlarged (X) signs in **B**, **D** indicate the query compound of KNN analysis; green: DB00006, orange: DB00849, purple: DB00977, aqua: DB01362, blue: DB04837. ( +) signs indicate the NNs of a query compound with identical color. Compounds are labeled according to Fig. 1

**Fig. 10:**
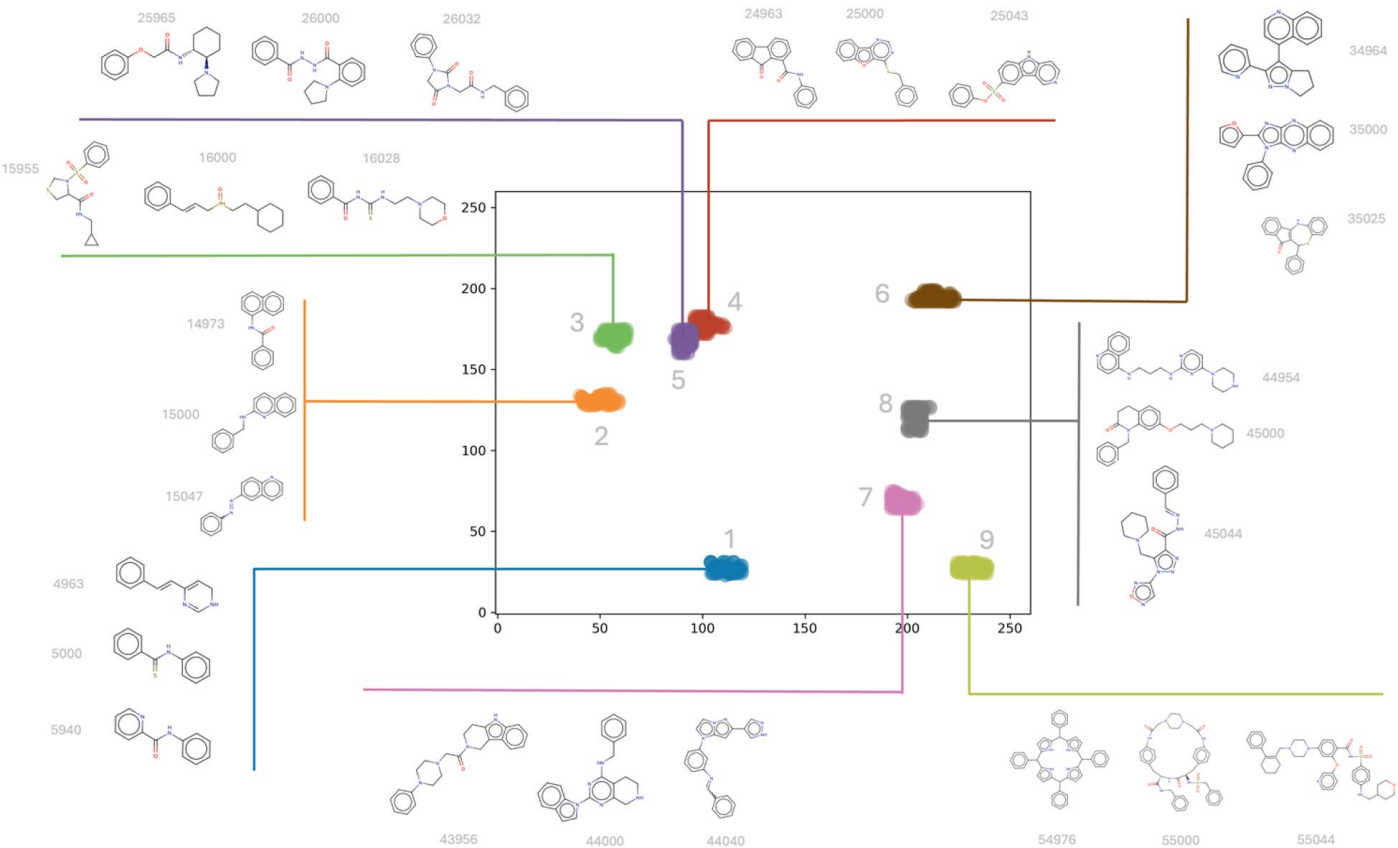
HCASE space defined by ChEMBL scaffolds, annotated by structures. The HCASE embedding of ChEMBL scaffolds at *z* = 8, shown in Fig. 9a, is annotated by structures. The cherry-picked scaffold set is colored according to colors provided in Table 1. The colors of the cherry-picked scaffolds were used to indicate their respective 100 SK-ordering based nearest neighbors. Structures were annotated for the cherry-picked scaffolds and two of their randomly selected neighbors among the 100 SK-ordering based nearest neighbors for demonstration purpose. Among each group of three scaffolds, the middle one is the cherry-picked scaffold. The structures of all 100 SK-based nearest neighbors are provided in Figs. S14–S22., in SI

To this end, we generated and compared the HCASE and *t*-SNE embeddings of the ChEMBL scaffolds. Of note, the *t*-SNE embedding operates on the Morgan-fingerprints of the ChEMBL scaffold set, which approach is independent from utilizing SKs in the case of the HCASE method. Results of the embeddings are shown in Fig. 8A and Fig. 9A. For better visibility, we only indicated a subset of ChEMBL scaffolds, namely those that belong to the cherry-picked scaffold set (see: section "*Computational Datasets and Methods*"), and the coloring schemes are identical across the two figures. Furthermore, Fig. 10*.* shows example structures of the cherry-picked scaffolds, whereas *Fig. S14-S22.* in *SI* shows all members of the respective series.

As shown on Fig. 8A, the positions of the 100 SK-ordering based nearest neighbors belonging to a particular cherry-picked scaffold are scattered. Likely, this is not something a medicinal chemist would expect in a map. Further, the logic regarding the relative positioning of scaffolds in the *t*-SNE map is not transparent, therefore it is difficult to intuitively interpret the resultant map produced by *t*-SNE embedding. As shown on *Fig. S8* in SI this phenomenon was observed across a range of perplexity values that were suggested as optimal for *t*-SNE [7, 45].

In contrast to the *t*-SNE embedding of ChEMBL scaffolds, in the map resulted from HCASE embedding the cherry-picked scaffold set demonstrates a great degree of clustering, see Fig. 9A. This is likely what a medicinal chemist would expect, that is, similar scaffolds are placed closely on the map, whereas dissimilar ones are placed further. In Fig. 9A a PHC of $z=8$ was used for the HCASE embedding. Further results related to the HCASE embedding of the same ChEMBL scaffolds shown in Fig. 4 obtained by varying the order of PHC. In Fig. 4*.* the same cherry-picked scaffold set is highlighted in the maps as in the case of Fig. 9A. As it can be seen, maps of great degree of clustering emerged where the order of PHC was at least 6 (see: Fig. 4E–G).

In the second scenario, we investigated how robust the embeddings generated by the HCASE and the Scaffold *t*-SNE methods are against the change in dataset to be embedded. To this end, we chose the "reduced scaffold set" (see: section "*Computational Dataset and Methods*") as the subject of the embedding, which set is a ~ 90% sized subset of the ChEMBL scaffolds utilized in the previous scenario. The difference between the two scaffold sets is less than 10%, which is a relatively small difference. Note, that the reduced scaffold set includes the entirety of the cherry-picked scaffold set for consistency reasons, as it was described earlier in the text. The results of the *t*-SNE and HCASE embedding of the reduced scaffold set is shown in Fig. 8C and Fig. 9C respectively.

When comparing the *t*-SNE embedding of the ChEMBL scaffolds vs. the reduced scaffold set (compare: Fig. 8A and Fig. 8C) the two maps are visually quite different despite the relatively small difference in the datasets that were subject to embedding. This holds true for all perplexity values applied over the experiments (compare: Figs. S8, S10, in SI).

As compared to the *t*-SNE embedding, the HCASE embedding of the reduced scaffold set shows a clear contrast. That is, the position of the cherry-picked scaffolds in the HCASE embedding of the reduced scaffold set (see: Fig. 9C) barely changed compared to the embedding of the entire ChEMBL scaffold set (see: Fig. 9A), when using a PHC of $z=8$. This observation between the HCASE embedding of the ChEMBL scaffolds and the reduced scaffold set holds true for all order (2 $\le z\le 8$) of the employed PHCs (compare: Fig. 4 and Fig. S12, in SI). In summary, we can conclude that the HCASE method is more robust to changes in the dataset to be embedded as compared to the *t*-SNE method. This observation matches our experience with *t*-SNE from practice.

In the third scenario, we investigated the result of embedding a set of compounds into an already existing chemical space, generated by the respective methods. From a medicinal chemist's point of view, it would be desirable that the existing chemical space remained unchanged regardless of the nature of the compound set subject to embedding. With other words, a chemist would expect to see a certain chemotype being positioned in the same part of the map regardless of the dataset it comes from, as long as the datasets are to be embedded into the same underlying chemical space.

To exemplify this scenario, we intended to embed drug compounds into the ChEMBL scaffold space with the HCASE and *t*-SNE methods and compare the outcomes. While the HCASE method was devised to be able to embed compounds into an already existing chemical space *e.g.,* ChEMBL scaffolds, without altering it, the situation is different in the case of the *t*-SNE method.

In the case of *t*-SNE method, embedding of additional structures into an already generated *t*-SNE chemical space is not possible. One could, of course merge the drug compounds with the ChEMBL scaffolds and perform the *t*-SNE embedding of the resultant set. Two problems arise from this approach. First, we could no longer consider the two *t*-SNE chemical spaces as being identical. Therefore, the addition of drug compounds alters the chemical space. Second, we have shown in the previous scenario, the *t*-SNE embedding is not robust to the change of the dataset subject to embedding (see: Fig. 8A, C). The change of the chemical space will, therefore, likely result in the change of the relative positions of the chemotypes as compared to the original map. A medicinal chemist would likely not expect the change in the positions of chemotypes in the underlying chemical space.

To address the above issues, we found it necessary to modify the original *t*-SNE algorithm. The idea behind the modification is to enable the *t*-SNE embedding of compounds into an existing *t*-SNE chemical space without altering it. Of note, this modification could be implemented in the context of other space embedding methods, the discussion of this is outside the scope of this study. Nonetheless, the modification involves the *t*-SNE embedding of a reference scaffold set, *e.g.,* ChEMBL scaffolds, which embedding will constitute the *t*-SNE chemical space. Once this is established, any compound set can be embedded to this *t*-SNE chemical space utilizing a mechanism borrowed from the HCASE method. That is, a compound in the resultant *t*-SNE embedding assumes the position of those reference scaffold whose SK-distance is the closest to the BMS of the compound.

This altered *t*-SNE method is referred to as ‘‘Scaffold *t*-SNE’’ throughout the text and is described in detail in section ‘‘Scaffold *t*-SNE Method’’, in SI. The modifications implemented in the Scaffold *t*-SNE method minimize the differences as compared to the HCASE method. In fact, the difference in the placement of the compounds in the resulting maps can be solely explained by the differences in the positions of the reference scaffolds in the HCASE and *t*-SNE embeddings. This facilitates the comparison of the results of the HCASE and Scaffold *t*-SNE embeddings.

In a comparative analysis, we performed the Scaffold *t*-SNE and HCASE embedding of drug compounds into the ChEMBL scaffold space. In the resultant maps we highlighted the positions of 5 randomly selected compounds and their *k* = 5 nearest neighbors that were described in section ‘‘*Embedding of KNNs*’’. The results of the Scaffold *t*-SNE and HCASE embeddings are shown in Fig. 8B and Fig. 9B, respectively. Interestingly, in the Scaffold *t*-SNE embedding (see: Fig. 8B) a high degree of clustering can be observed in all KNN-series that is comparable to that produced by the HCASE method (see: Fig. 9B) using a PHC of $z=8$. The reason for this is that Scaffold *t*-SNE method takes advantage of the predefined chemical space when mapping compounds to the closest reference scaffolds. Therefore, the embedding will reflect the differences and similarities of chemotypes to a great degree. This observation was true in the case of all the applied perplexity values (see: Fig. S9, in SI).

While the clustering properties of the HCASE and Scaffold t-SNE embedding methods seem comparable, it is difficult to explain the reasons leading to the relative positioning of the 5 drug molecules and their KNNs in the Scaffold *t*-SNE map. In the case of both methods, the positions of the compounds are determined by the positions of the reference scaffolds as discussed above. Unlike *t*-SNE, the HCASE method provides a transparent mechanism with regards to the laying out the reference scaffolds on the map. This mechanism is driven by the organization of scaffolds according to a medicinal chemistry viewpoint encoded into the Scaffold-Key algorithm.

As we have shown previously, *t*-SNE embedding is not robust against the change in the dataset subject to embedding. Also, we have discussed that the position of the embedded compounds in the Scaffold *t*-SNE embedding is determined by the position of the reference scaffolds in the *t*-SNE space. Consequently, the Scaffold *t*-SNE method is expected not to be robust to the change in the underlying reference scaffold set either. This phenomenon can be observed by comparing Fig. 8B and Fig. 8D. The latter shows the embedding of the 5 drug compounds and their KNNs into a chemical space defined by the reduced scaffold set. Despite the relatively small difference between the underlying reference scaffold sets (ChEMBL scaffolds vs. reduced scaffold set) the change in the positions of compounds is obvious when comparing Fig. 8B and Fig. 8D. Beyond the rotation of the map, which seems to be major cause for the “rearrangement” of the compounds, there are a few examples when the relative positions of compounds also changed.

For example, in the “cyan series”, the relative positions of “S”, “P” and the query compound (plotted by a large cross symbol) are quite different comparing the embeddings shown on Fig. 8B and Fig. 8D*.* While “P” is slightly offset from a line defined by “S” and the series marker in Fig. 8B, these same three compounds define a pronounced triangle in Fig. 8D. This “rearrangement” goes beyond simple rotation and/or “zooming in/out” effect(s). Another example involve the “X “, “V”, and query compound of the “blue series” and “P” from the “cyan series”. In *Fib. 8B*, “X” and “V” are positioned closer to “P” than to the query compound of the “blue series”. In contrast, “X” is closer to the query compound in Fig. 8D than to “P”. Also, “V” is much closer the query compound, and farther from “P” than in Fig. 8B.

These demonstrate, that the Scaffold t-SNE embedding is not robust to the change in the underlying reference scaffold set. This makes the interpretation of embedding challenging for medicinal chemists. Although the perplexity value was set to 40 in the experiments resulted in Fig. 8B and Fig. 8D., similar examples can be observed at other perplexity values (compare: Fig. S9 and Fig. S11, in SI).

In contrast to the Scaffold-*t*-SNE embedding, the HCASE embedding ($z=8$) of the drug compounds and their KNNs into the reduced scaffold space remained robust as compared to their embedding in the ChEMBL scaffold space (compare: Fig. 9B and Fig. 9D). One difference involves the slight change in the relative positions of compound ‘‘P’’ and ‘‘S’’ to each other. Despite the change in positions, "P" and "S" is still closer related to each other than to any other compounds of the series highlighted on the maps. Another slight difference involves a triangle defined by compounds "L", "V" and "X". Although a slight change is visible in the positions of the nodes of this triangle, overall, the relative positions of the three compounds to each other and to other compounds on the map remains arguably stable. These observations hold true for PHCs of varying order (compare: Fig. 5 and Fig. S13, in SI).

The final viewpoint for comparison relates to the convergent properties of the embedding methods. The HCASE methods operates with the help of PHCs. Points mapped to PHCs of increasing order (parameter *z*) are known to converge in the higher dimensional space the PHCs are embedded (folded) into. Indeed, this property is clearly reflected in the maps generated by the HCASE method (see: Figs. 4, 5 and Figs. S12, S13, in SI), since the position of compounds converges (stabilizes) by increasing the values of parameter *z*, *i.e.,* the order of the employed PHC. As discussed earlier in the text, increasing the value of parameter *z* can be thought of as increasing the resolution of the map.

In the case of Scaffold *t*-SNE and t-SNE methods there is no obvious parameter that would affect the resolution of the map. Nonetheless, perplexity is a parameter known to affect the embedding outcomes, and we have shown results obtained by varying this parameter. However, we have not observed the convergence in the positions of the embedded compounds in the relation to varying the value of perplexity. On the contrary, varying the value of perplexity led to results that are likely confusing to medicinal chemist in the light of the rearrangements of the maps.

In summary, the embeddings produced by the HCASE and Scaffold *t*-SNE methods differ in three major standpoints.

First, the HCASE embedding provides a transparent and medicinal chemistry inspired mechanism regarding how the chemotypes are arranged in map that arose from embedding process. The same cannot be stated for Scaffold *t*-SNE method and for its predecessor, the *t*-SNE method.

Second, the (relative) position of the coordinates of the embedded molecules produced by the Scaffold *t*-SNE method does not seem to converge, i.e., to stabilize, by varying the value of perplexity parameter. This feature of the Scaffold *t*-SNE method does not promote the intuitive interpretation of the results and is in great contrast with the converging property of embedded coordinates produced by the HCASE method.

Finally, the HCASE method allows for embedding any additional dataset into an existing chemical space without altering it. This is not true for the *t*-SNE method, although modification can be introduced to alleviate this limitation, as we demonstrated in the case of the Scaffold *t*-SNE. However, the HCASE embedding seems more robust to the change in the underlying chemical space (reference scaffolds) as compared to the Scaffold *t*-SNE method.

It can be concluded from the comparison of the two methods that existing space embedding methods can be modified successfully to produce embeddings with reasonable clustering properties for chemotypes. Still, the HCASE method provides a clear advantage for interpretability.

## Conclusions

In this proof-of-concept study we present a HCASE space embedding method that stands out from existing methods by its unique ability to produce an embedding that can be easily interpreted by medicinal chemists and data analysts. The novelty of the method is to create a well-defined latent grid of reference scaffolds, where the scaffolds are organized by increasing structural complexity. This is achieved by mapping the reference scaffolds based on their scaffold keys to a pseudo-Hilbert-Curve that can be readily embedded into higher dimensional space according to a well-established algorithm. Compounds are subsequently embedded into this grid based on their proximity to reference scaffolds measured by Scaffold-Key distances.

With the help of a series of experiments, we demonstrated that the HCASE method indeed meets all the criteria we set forth for an intuitive space embedding method. Namely, the embedding is able to cluster related chemotypes, and to lay out the chemotypes in a logical order in the embedded space. The ability to use a reference scaffold set to define a chemical space assures that independent compound libraries can be embedded into the same space in a consistent manner. This allows for direct comparison of the embeddings of different datasets visually, qualitatively and quantitatively, as long as the underlying reference scaffold set remained the same. Furthermore, the HCASE method is able to generate a series of embeddings with increasing resolutions. In these series the positions of compounds converge as the resolution increases, which is not a property that has been accomplished by the other methods. We have also demonstrated that it is possible to quantify the distances between the embedded points in the HCASE space by computing the pairwise Chebyshev-distance values.

The chemotype-clustering ability of HCASE method was characterized with the help of two reference scaffold sets (ChEMBL: 63,783 scaffolds, NatProd: 546 scaffolds) and two compound libraries (DrugBank: 2073 compounds, CANVASS: 344 compounds). The analysis of embedding KNN series has shown that HCASE method is able to cluster closely related structures in the embedded space. As expected, the degree of clustering was higher in the KNN series as compared to a series of randomly selected molecules. Also, we compared the overlap of the HCASE embedding of the two compound libraries in two different reference scaffold set spaces. The results demonstrated that reference scaffold sets can be used to define a perspective for embedded space comparison, e.g., to compare embeddings in a natural product space. Furthermore, we provided the means to compare HCASE embeddings quantitatively.

Finally, we compared the properties of space embeddings generated by HCASE method and a prior art method, which was modified for the sake of meaningful comparison. We found that the qualitative clustering properties of the modified prior art method was nearly as good as that of the HCASE method. However, the results of the HCASE method can be easily interpreted from a medicinal chemistry point of view, unlike the results of the other method.

In conclusion, the presented HCASE method is attributed with novel and unique characteristics that can render it as a desirable data reduction and clustering method in any research setting where medicinal chemistry perspective is essential.

## Outlook

In light of the structurally interpretable property of the HCASE method, it would be a natural extension to create interactive visualization of results. That is, when selecting a region of interest on the embedding plot, the underlying scaffold(s) could be visualized in an application to provide more structural context for the position of embedded compounds. Furthermore, inspired by SOM and GTM method, it might be helpful to quantify how well the chemotype of an embedded compound matches that of the reference scaffolds associated with that position. This property might be the mean of distances computed between a given compound and the reference scaffolds associated with its position.

### Supplementary Information

Supplementary material 1.

## Declarations

### Availability of data and materials

The source code of the HCASE method, Jupyter notebooks of the experiments, input and output files can be found at https://github.com/ncats/hcase.
