# Supplementary material for: Hilbert-curve assisted structure embedding method
Source: J Cheminform. 2024 Jul 29;16:87. doi: 10.1186/s13321-024-00850-z (PMC11285582; doi:10.1186/s13321-024-00850-z)
Supplement: Supplementary file 1 — Supplementary material 1. [file 13321_2024_850_MOESM1_ESM.pdf]

Supporting Information

Hilbert-Curve Assisted Structure Embedding Method

Gergely Zahoránszky-Kőhalmi*, Kanny K. Wan and Alexander G. Godfrey

National Center for Advancing Translational Sciences (NCATS/NIH), 9800 Medical Center Dr., Rockville, MD 20850, USA

*Corresponding author

Gergely Zahoránszky-Kőhalmi

Email: gergely.zahoranszky-kohalmi@nih.gov

APPENDIX

The following remarks needed to be made regarding the in-house implementation of Scaffold-Key algorithm [1], [2]. When a specific key is referred in the remarks then the key ID reflects the order of the keys as published by Ertl [1].

- Rings were determined as “smallest set of simple rings for a molecule” (SSSR) as implemented by RDKit [3].
- The following atoms were considered as heteroatoms in implementing the Scaffold-Key algorithm: Li, Be, B, N, O, F, Mg, Al, Si, P, S, Cl, Zn, As, Se, Br, Te, I, Pt, Hg, Mn, Fe, Co, Ni, Cu, Ga, Ge, Rh, Pd, Ag, Cd, Sn. Relevance: many keys related to heteroatoms.
- Definition of “multiple linker” was not provided by the Scaffold-Key algorithm. Hence, we quantified this property as the number of bonds associated with the branched linker atom. Relevance: key 19.
- In fully conjugated rings, the number of bonds was determined as follows. In RDKit, the conjugated system is associated with an ID. If all members of a ring are assigned to the same ID then the ring was considered as fully conjugated, otherwise as not fully conjugated. Relevance: key 7, 8.
- Number of multiple bonds in not fully conjugated rings: double, triple and aromatic bonds were all counted. Relevance: key 8.
- Exocyclic atoms: atoms connected to rings with double bond. Relevance: key 31.
- Exolinker atoms: atoms connected to linker substructure with double bond (exolinker is not part of the linking substructure). Relevance: key 31.
- Heteroatoms associated with more than two bonds: total number of non-hydrogen bonds for heteroatom. Relevance: key 32.

Embedding of Drug Molecules with *t*-SNE Algorithm

Approved drug structures were collected from the DrugBank database (version: 2.0.9) [4] database giving rise to a dataset of 2,073 structures. Standardization of structures involved keeping only the largest compound of each substance and was performed in KNIME [5] with the help of CDK nodes [6]–[9]. Molecule structures were depicted with RDKit and ChemAxon's Marvin Sketch [10]. Using the RDKit implementation of Morgan algorithm [3], [11], Morgan-fingerprint was generated for compounds with parameters of radius = 3 and fingerprint length = 2,048.

With the help of the generated fingerprints the t-SNE embedding of molecules was generated using a series of perplexity values: 5, 10, 20, 30, 40, 50 [12]. The t-SNE embedding was generated using the implementation provided by the SciKit Python library [13], [14] (see: *Fig. S1*). Other parameters of the algorithm were set to their default values, i.e., learning rate = 200, iteration number 1,000.

A random subset comprised of the 90% of the DrugBank molecules was selected randomly. Compounds highlighted in Fig. S1 were merged with the subset to assure consistency. The *t*-SNE embedding of this set was also generated (see: *Fig. S2*) using the same perplexity values and parameter setting.

Scaffold *t*-SNE Method

For the sake of comparison with the HCASE method, we implemented a variation of the *t*-SNE method. The modification involves the use of a reference scaffold set to serve as landscape objects for the embedding of molecules. This modification intends to convert the *t*-SNE method in a way that better represents a medicinal chemistry inspired embedding and that also enables a consistent embedding mechanism of molecules regardless of the input molecule set at hand.

In the first stage of this method, the *t*-SNE embedding of a reference scaffold set is established. Next, the Scaffold-Keys of the reference scaffold set are computed. In the second stage, the scaffold-keys of compounds to be embedded are computed. The d_{SK} is computed between each compound and reference scaffold. Compounds assume the embedded coordinates of the closest reference scaffold according to d_{SK}.

The modified *t*-SNE method is referred-to-as *Scaffold t-SNE* method throughout the main and SI text. The pseudocode of the Scaffold t-SNE method is provided in "*Pseudocode of the Scaffold t-SNE Method*" section in SI, adopting the same notions discussed in section "*Pseudocode of the HCASE Method*" in main text.

The Scaffold *t*-SNE analysis was performed at various *perplexity*-values of {5, 10, 20, 30, 40, 50} which are considered as optimal for t-SNE. [15], [16] The rest of the parameter settings were left to default as defined by "SciKit" (Python library) implementation of the *t*-SNE algorithm [13], [14]. Note, that the default values of learning rate and the number of iterations are: 200 and 1,000, respectively.

Pseudocode of the Scaffold *t*-SNE Method

Algorithm 2 Scaffold t-SNE Method

```
Input: set of molecules M
Input: set of reference Bemis-Murcko scaffolds S

Variable: molecule mol
Variable: scaffold-key sk
Variable: list of (S, sk)-tuples S_SK
Variable: Bemis-Murcko scaffold bms
Variable: (x ∈ ℕ, y ∈ ℕ)-tuple P
Variable: list of P items T
Variable: list of (sk, P)-tuples E_scaffold
Variable: fingerprint fp
Variable: list of fingerprints F
Variable: list of (mol, T_coord) E

for all S_i in S do
        sk := getScaffoldKey (S_i)
        S_sk.add (S_i, sk)
        fp := generateFP(S_i)
        F.add(fp)
end for

S_sk := sort S_sk alphanumerically by SK in increasing order
S_sk := deduplicate S_SK by sk, keep first instance of identical tuples
T := t-SNE (F)
E_scaffold := (S_SK , T )

for all m_i in M do
        bms := getBemisMurckoScaffold (m_i)
        sk := getScaffoldKey (bms)
        P := getClosestReferenceBMSCoordinate (sk, E_scaffold)
        E.add (m_i, P)
end for

return (E)


int function getClosestReferenceBMSCoordinate(sk, E_scaffold)
        Variable: (x ∈ ℕ, y ∈ ℕ)-tuple P_mol
        Variable: numeric d_SK
        Variable: list of (P, sk, d_SK)-tuples D

        for all e_i in E_scaffold do
                d_SK := getSKDistance (sk, e_i[0])
                D.add (e_i[1], e_i[0], d_SK)
        end for
        D := sort D by d_SK and sk, both in increasing order
        P_mol = D[0][0]

return (P_mol)
```

Distance Rank Correlation Analysis

In response to Reviewers 2's question [30] as to investigate the ability of the HCASE method to preserve relationships between closely and distantly related compounds in the embedded 2D space we performed an analysis as follows.

In the context of a given HCASE space and compound set to be embedded, we determined the *rank distance* (see: *Eq. 7*, main text) between pairs of compounds based on their position on the unfolded pseudo Hilbert-curve. The position of a compound on the unfolded pseudo Hilbert-curve assumes the position of the reference scaffold that is closest to the compounds' Bemis-Murcko scaffold [31] according to the Scaffold-Key distance [1] as described in the main text. The *rank distance* of two compound is the absolute value of their respective positions on the unfolded pseudo Hilbert-curve.

The distance between compounds in the embedded 2D HCASE space is measured by the *Chebyshev-distance* (see: *Eq 8.*, in main text) [32], as described in the main text.

Having these distances computed, for each compound *C* we ranked the rest of the compounds with respect to their *rank distance* to *C*. In case of ties, all values were assigned the same rank, and the next rank value was incremented by the number of values causing the tie at hand ("min" ranking method by Pandas Python library [13, 33]). We repeated this process but now we ranked the compounds based on their *Chebyshev-distances* to *C*. This process gave rise to two "distance rank" series for each compound. Using these series, we were able to get an insight into how the HCASE embedding preserves close and long-distance relations.

To highlight this information, and to somewhat compensate for rank value artifacts introduced due to resolving ties, we transformed the two distance rank series by dividing them according to 10 quantiles,

using the “qcut” method by Pandas [32]. We refer to these values as *rank distance* and *Chebyshev-distance* rank quantiles, respectively.

Finally, we constructed heatmaps via the Seaborn Python library [33], by aggregating the number or occourances where the specific *rank distance* and *Chebyshev-distance* rank quantile pair was observed in a given chemical space and compound library combination. The x- and y-axis show the *rank distance* and the *Chebyshev-distance* rank quantiles, respectively.

As it can be seen in all four cases in *Fig. S23*, the HCASE method preserves close distances very well, as expected. Similarly, long distance relationships are also preserved reasonably well. These are indicated by relatively densely populated upper-left and lower-right 3x3 sub-matrices (segments). Between these segments the diagonal is also reasonably well populated, and areas distant from the diagonal are quite underpopulated (though not entirely empty), as one would expect for a reasonable embedding performance.

Taken these observations together, we conclude that the HCASE method is capable of maintaining close-distance and long-distance relationships to an acceptable extent.

Detection of “Canyons” on the 2D Maps

In response to Reviewer 2’s question [30] as to investigate points in close proximity on 2D map where the *rank distance* (see: *Eq. 7*, main text) is relatively large despite the close proximity, we performed an analysis as follows.

The subject of the analysis was a pseudo Hilbert-curve of order (z) = 8. The process of folding it into 2D space gives rise to x, y coordinates in the range of [0, 255], and accordingly to 65,536 possible unique

points. Considering that the reference scaffolds, and eventually the compounds are mapped to one of these points on the unfolded pseudo Hilbert-curve, the maximal rank distance between any two points can be 65,535.

We defined the relatively large *rank distance* with the help of a factor of values 0.05, 0.25, 0.5. That is, if the “rank distance” between any two points was found greater than the maximal “rank distance” (65,535) multiplied by the factor, then the two points were deemed as being relatively distant based on their “rank distance”.

The premise of the HCASE method is the closely related compounds will be placed closely, where distantly related points distantly in the emerging 2D map. Due to the folding of the pseudo Hilbert-curve, distance between points in the 2D map are no longer characterized by their *rank distance*, the well-known *Chebyshev-distance* (think of it as distance on a grid) is a better alternative to measure distances, as the points in the 2D map are actually mapped to a grid. Reviewer 2 raised the issue to highlight areas of the 2D map where we find points in close proximity (according to *Chebyshev-distance*) but which points are the same time known to be relatively distant based on their *rank distance*.

To answer this question, we investigated the immediate neighbors of each point in the 2D map imposed by an order of 8 pseudo-Hilbert curve. First, we created a network of 65,536 nodes representing these unique points, and we used the x, y coordinates of the points as node attributes. Then, all 65,536 unique points and their maximal number of 8 neighbors (in a “Chebyshev-radius” of 1) were surveyed, where the point in the center is called the “central point”. If we observed that the central point and a neighbor point is considered relatively distant based on their *rank distance*, then we defined an edge between the respective nodes in the network. Once all central points and respective neighbors were surveyed, the network generation process was complete.

This network was visualized on a grid according to the node attributes, only nodes were shown that participate in at least one edge. With the addition of the (0,0) and (255, 255) points to indicate the "corners" of the map.

Considering that we use 3 different values for the factor to define relatively distant points based on their *rank distance*, we ended up with three different networks shown in *Fig S24*. As it can be seen, there can be observed areas where there is a certain degree of "discrepancy" between the *Chebyshev-distance* and *rank distance* between points. We refer to these areas as "canyons". Note, that the area where these canyons are located can be considered to introduce a low amount of distortion of the premise of space embedding, i.e. that similar compounds are placed closely whereas dissimilar ones at larger distances. Nevertheless, with the help of this analysis, we can indicate exactly where the investigator needs to exercise caution with respect to the abovementioned premise in the case of HCASE generated 2D maps.

Considering that the defining relatively large distances based on *rank distances* is somewhat subjective, we chose three reasonable, but increasingly strict values to help quantify this property. Namely, a factor of 0.50, 0.25 and 0.05 were chosen to analyze their effect on the size and number of the "canyons". As it can be seen in *Fig. S24*. When applying the most permissible factor of 0.50, only one canyon emerges in the middle of the map. Making the factor more stringent gives rise to more canyons. However, even at the arguably strict value of 0.05 the number of canons and their coverage of the overall map was deemed by us relatively low, hence acceptable. It is noteworthy to mention that the canyons also seem to follow a fractal pattern, which phenomenon deserves more in-depth analysis, and is beyond the scope of this study. Nevertheless, the proper justification of the fractal property of canyons would come not a surprise, considering the fractal properties of the Hilbert-curves.

We think the visualization of canyons would be a valuable feature of interactive HCASE maps in the future.

SUPPLEMENTARY FIGURES.

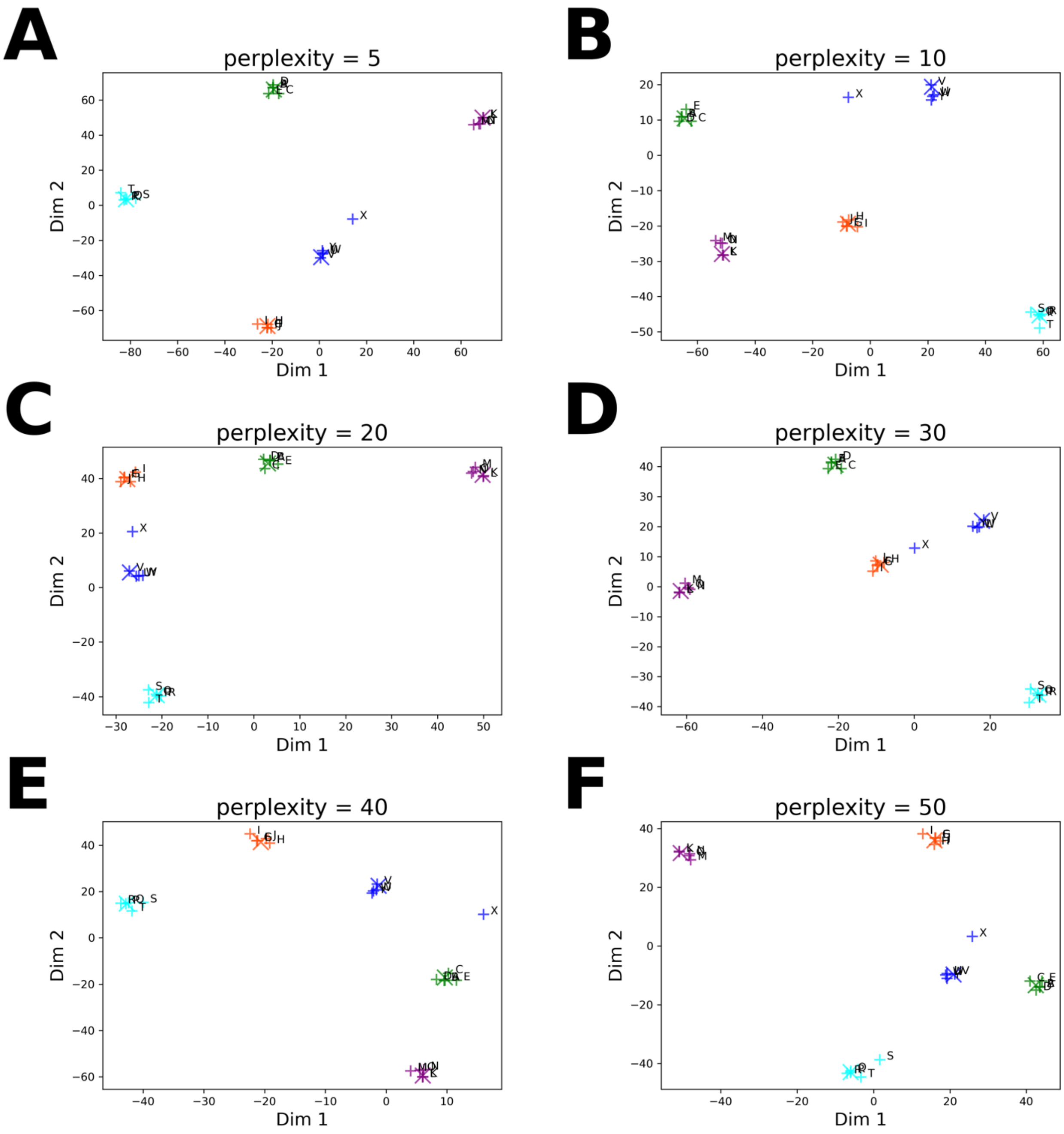

Figure S1. Original t-SNE analysis. Embedding of DrugBank molecules performed by the original t-SNE algorithm at various perplexity values. Enlarged (x) signs indicate the query compound of KNN analysis; green: DB00006, orange: DB00849, purple: DB00977, aqua: DB01362, blue: DB04837. (+) signs indicate the NNs of a query compound with identical color. Compounds are labeled according to Fig. 1 in main text.

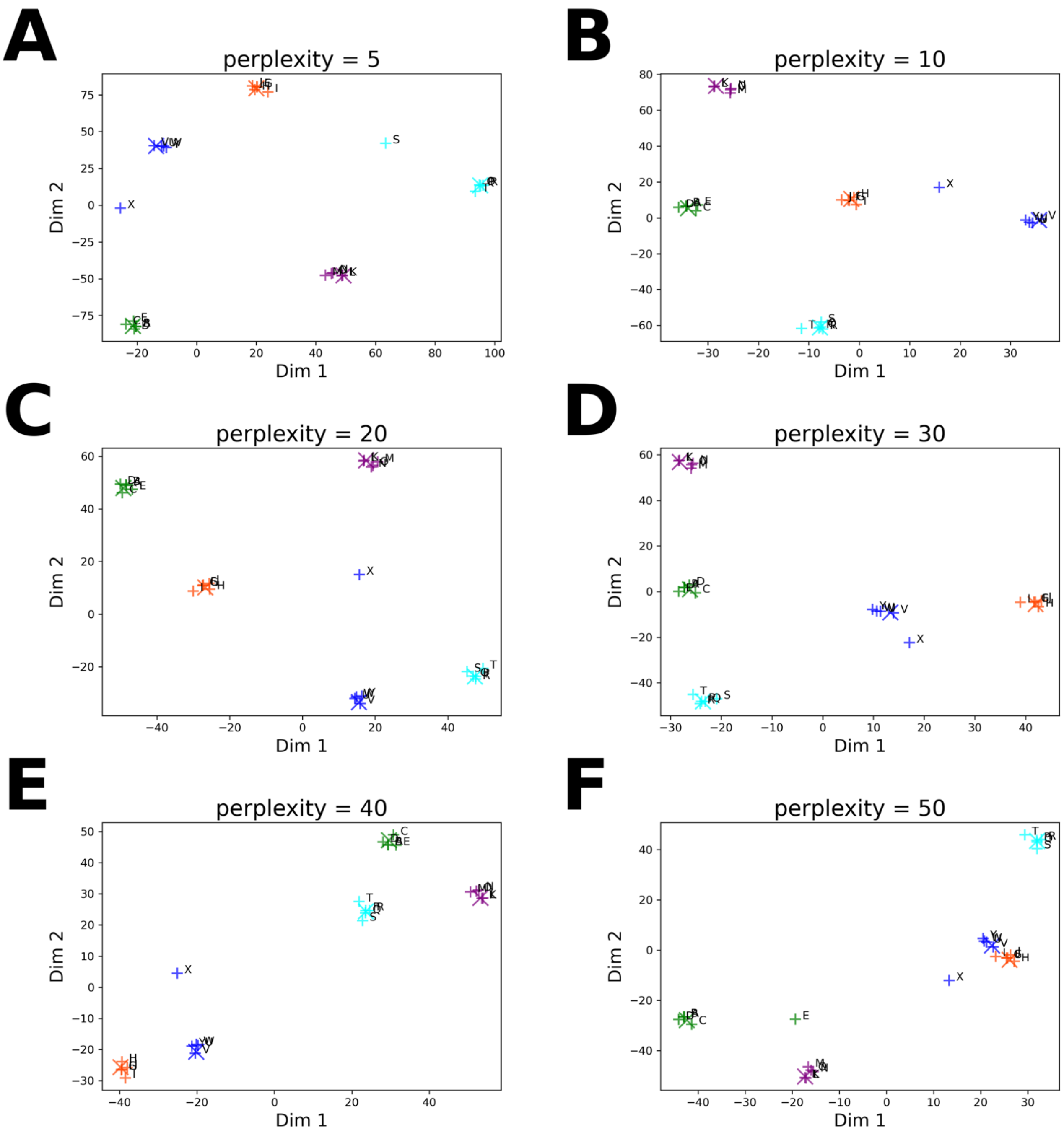

Figure S2. Original t-SNE analysis on a subset of DrugBank molecules. A random subset comprised of the 90% of the DrugBank molecules was selected randomly. Compounds highlighted in Fig. S1 were merged with the subset. This set was subject to t-SNE space embedding at various perplexity values. Enlarged (X) signs indicate the query compound of KNN analysis; green: DB00006, orange: DB00849, purple: DB00977, aqua: DB01362, blue: DB04837. (+) signs indicate the NNs of a query compound with identical color. Compounds are labeled according to *Fig. 1* in main text.

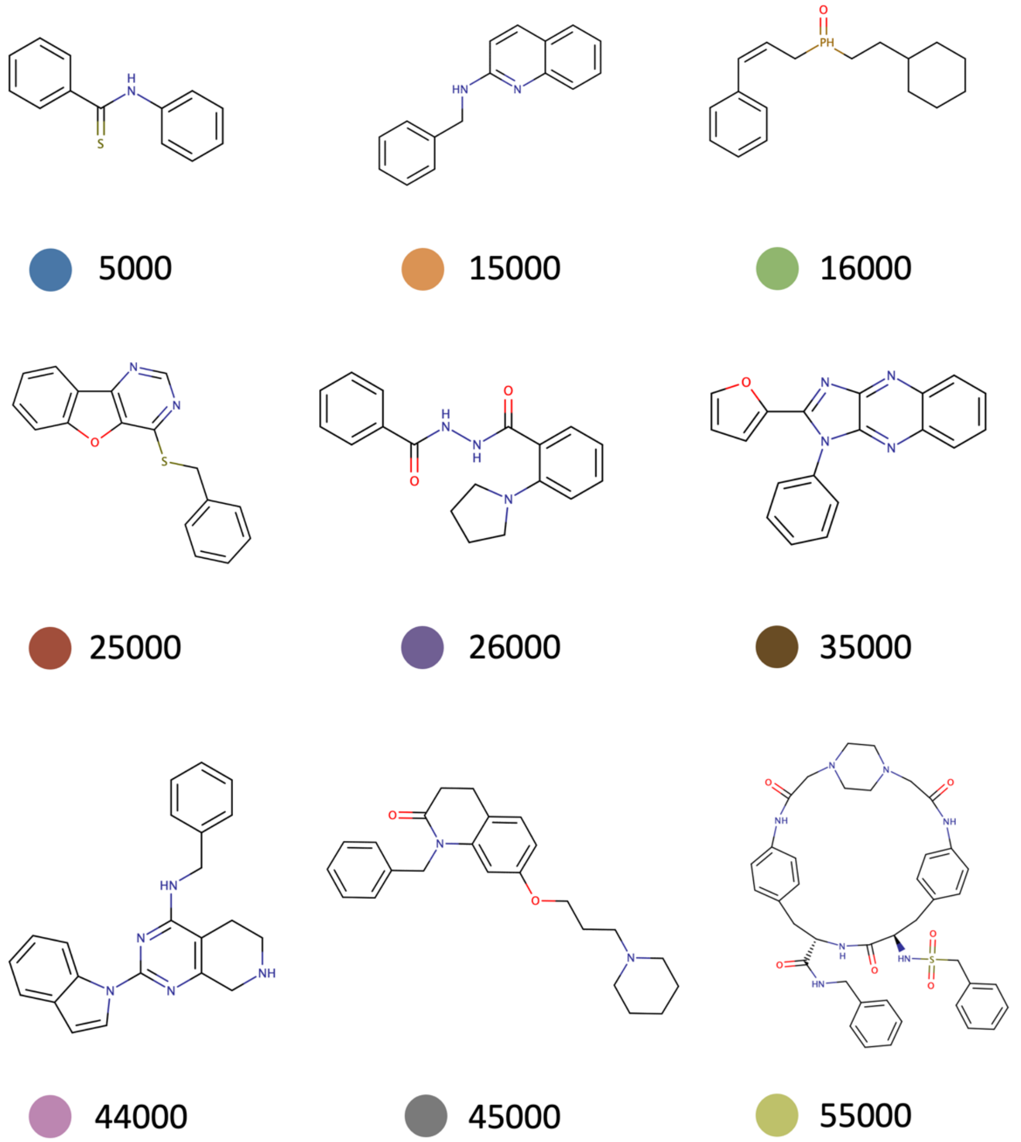

Figure S3. Cherry-picked scaffolds. Bemis-Murcko Scaffolds extracted from the ChEMBL 24 database [17] as described in Section "*ChEMBL Scaffolds*" in the main text. The Scaffold-Key based rank of cherry-picked scaffolds is shown below the structures. The color of the dots corresponds to the color of the cherry-picked scaffolds (and their 100 immediate neighbors) shown on *Fig. 4* in main text.

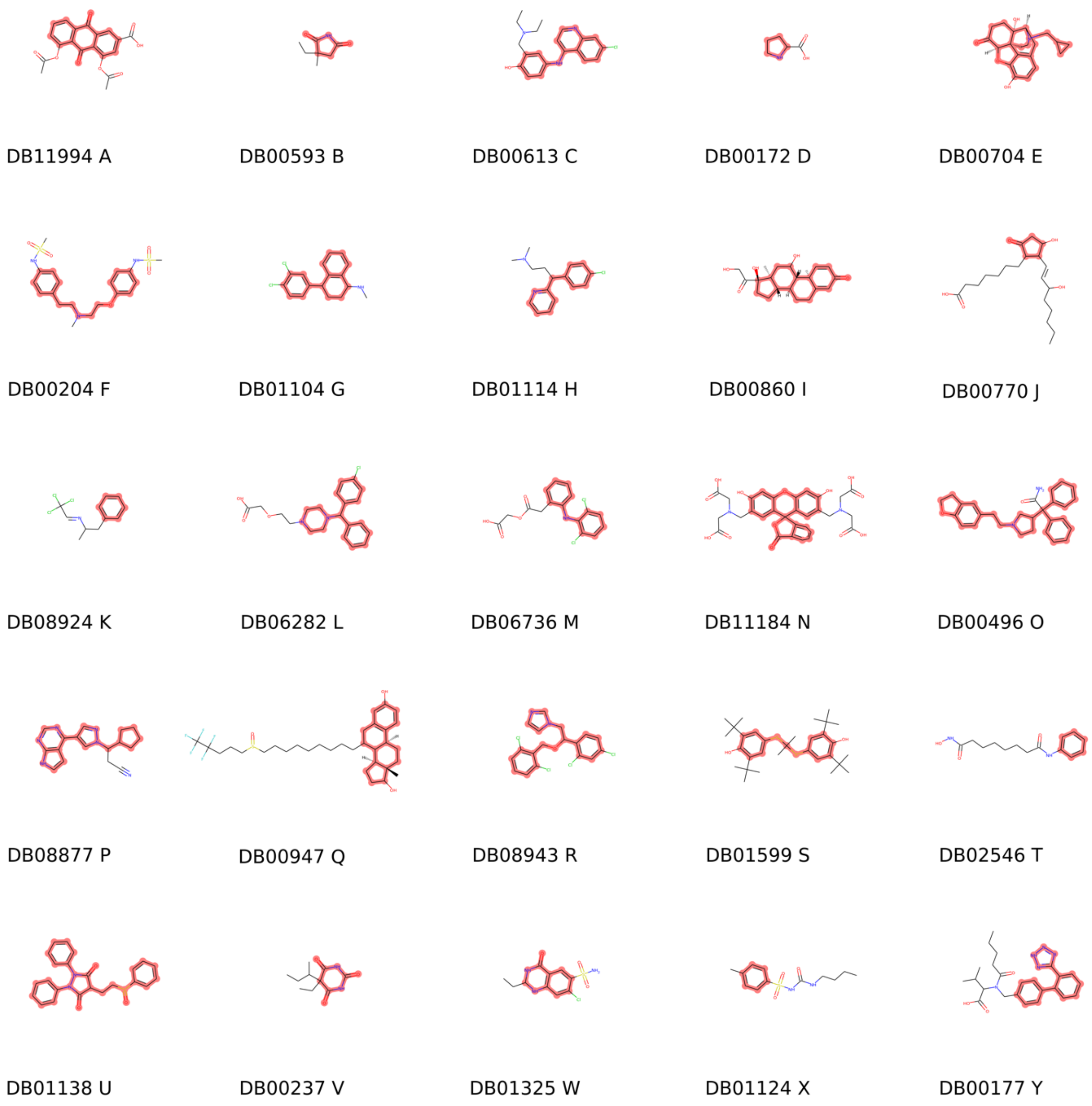

Figure S4. Embedding of 25 randomly selected molecules. Labels of compounds are shown after their IDs as they appear on *Figure S5*. The BMSs of compounds are highlighted by red.

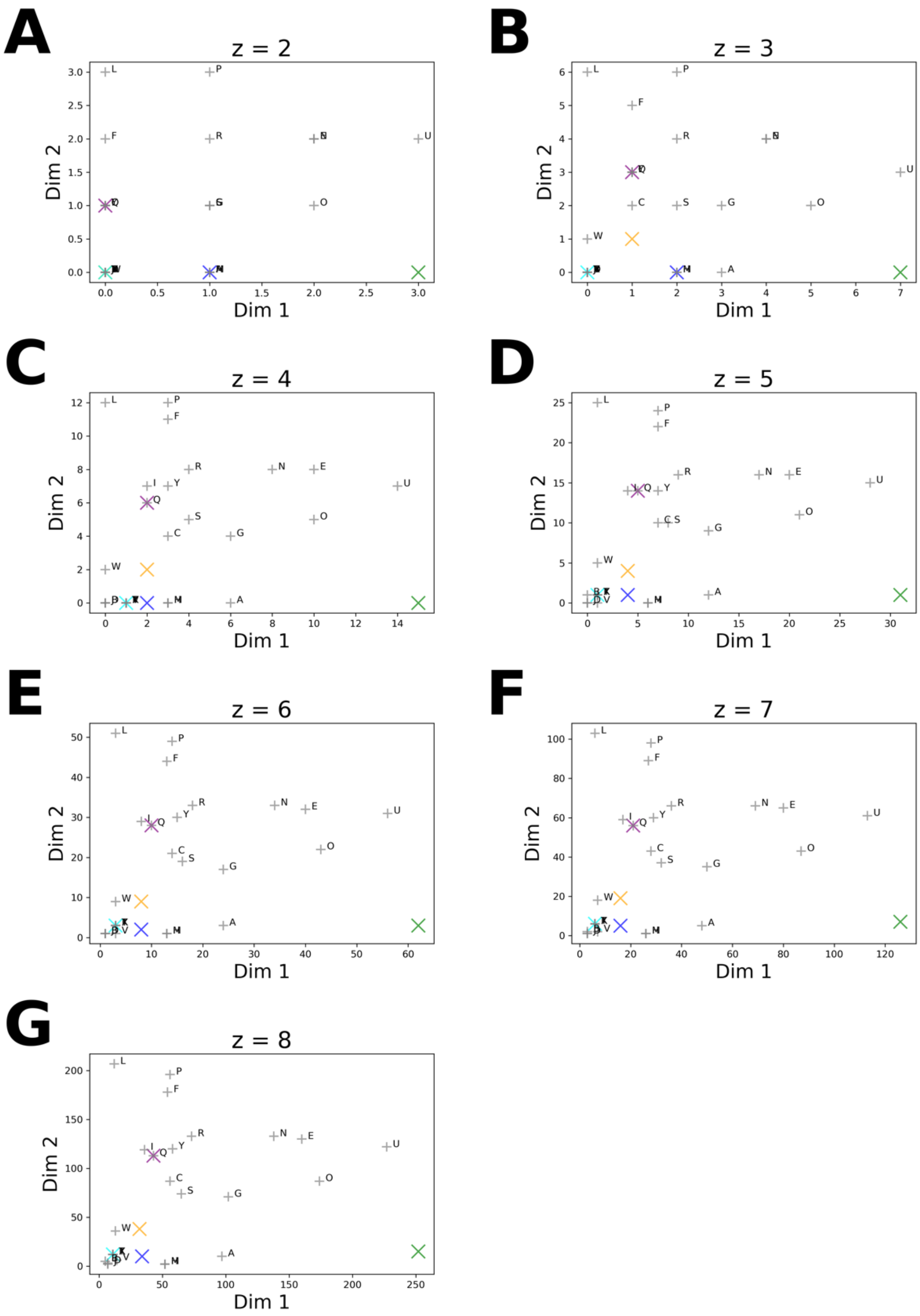

Figure S5. Analysis of randomly selected DrugBank compounds. Shown is the embedding of the 5 query compounds involved in the KNN analysis, and 25 randomly selected compounds. The order of PHC utilized for structure embedding is indicated by suffix. Enlarged (X) signs indicate the query compound of KNN analysis; green: DB00006, orange: DB00849, purple: DB00977, aqua: DB01362, blue: DB04837. (+) signs indicate the 25 randomly selected compounds with gray color. Compounds are labeled according to *Fig. S4*.

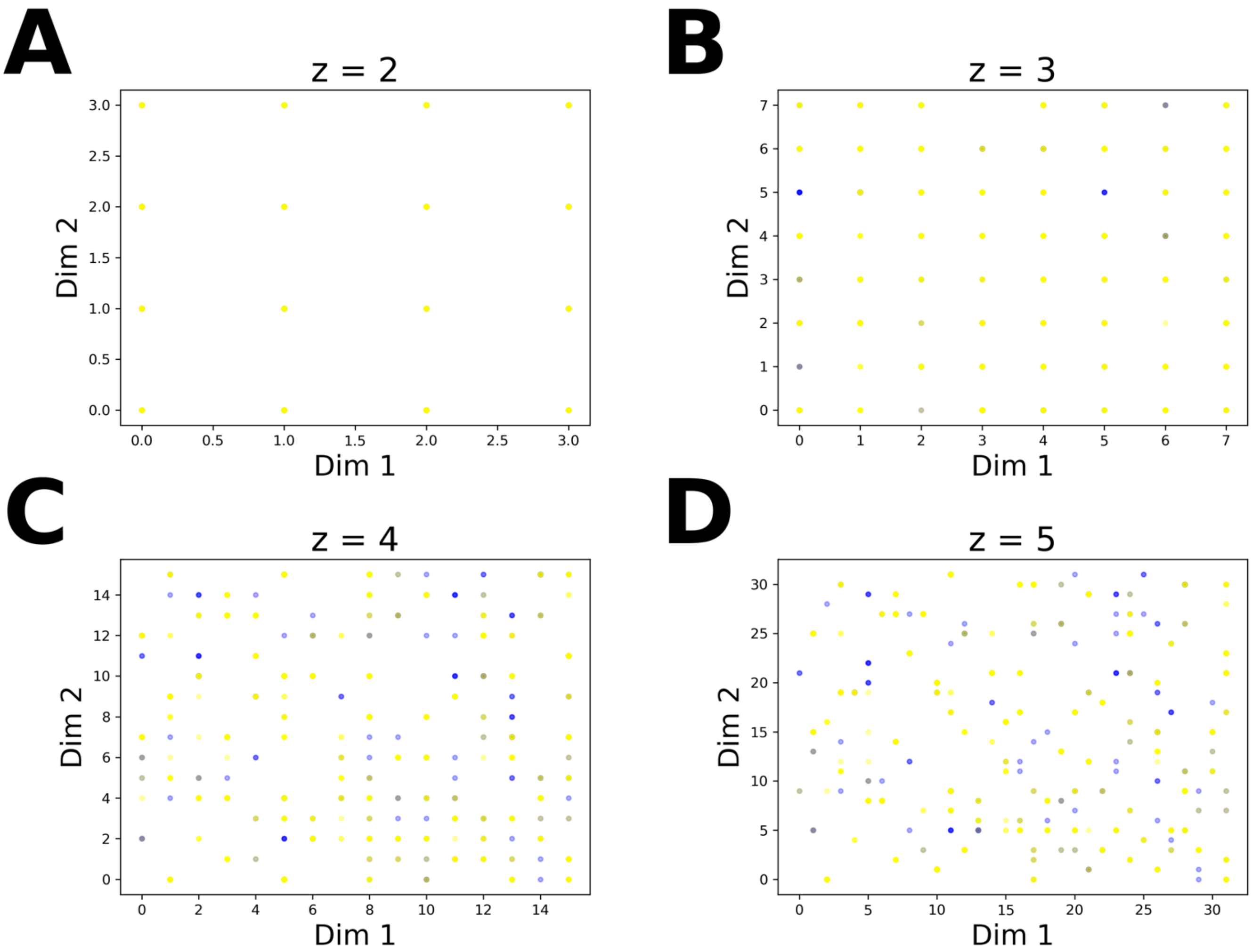

Figure S6. Comparing embeddings in NatProd space. Blue: CANVASS compounds, yellow: drugs. Overlapping datapoints are colored by green-brown color due to the transparency of the datapoints.

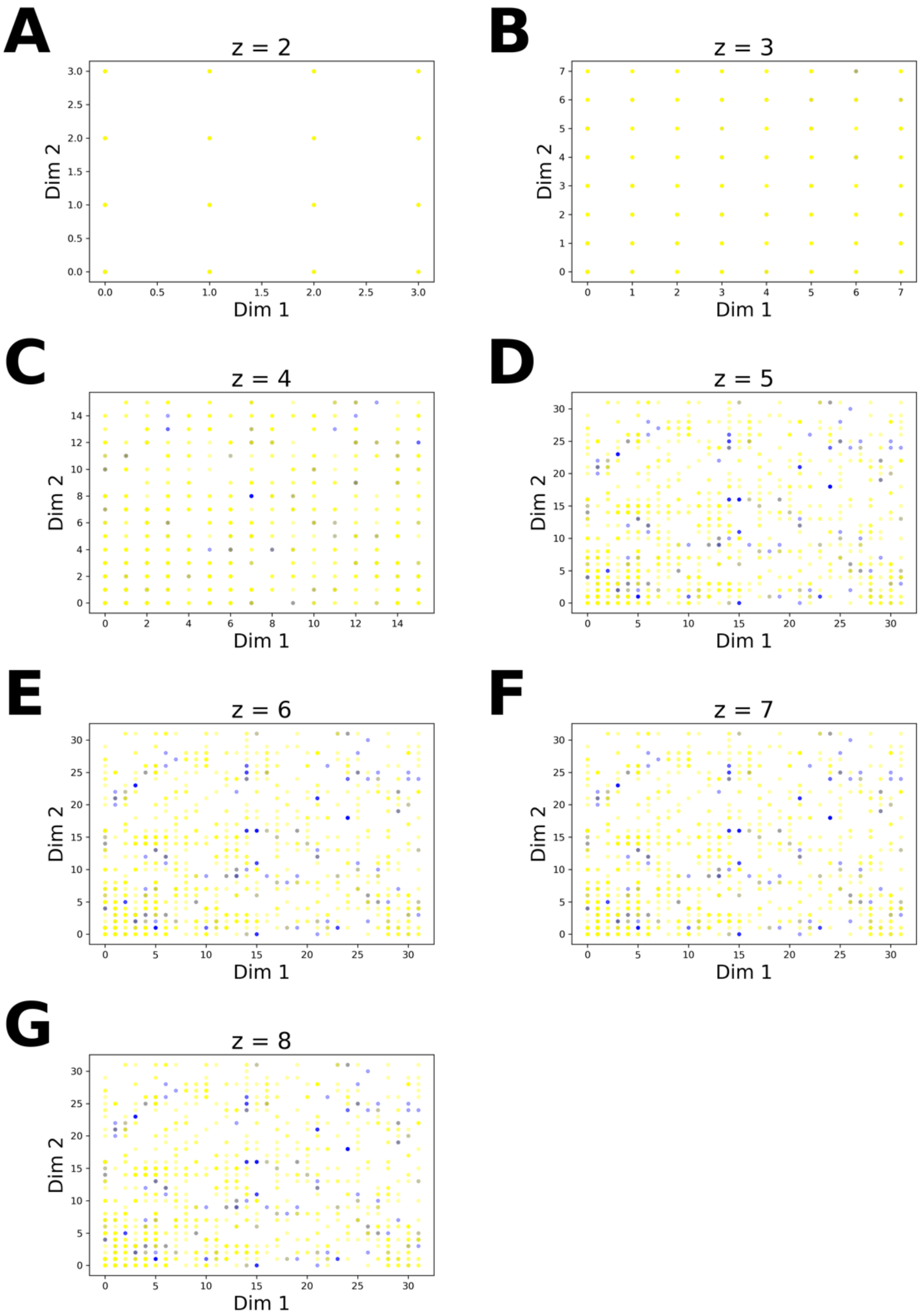

Figure S7. Comparing embeddings into ChEMBL 24.1 space. Blue: CANVASS compounds, yellow: drugs. Overlapping datapoints are colored by green-brown color due to the transparency of the datapoints.

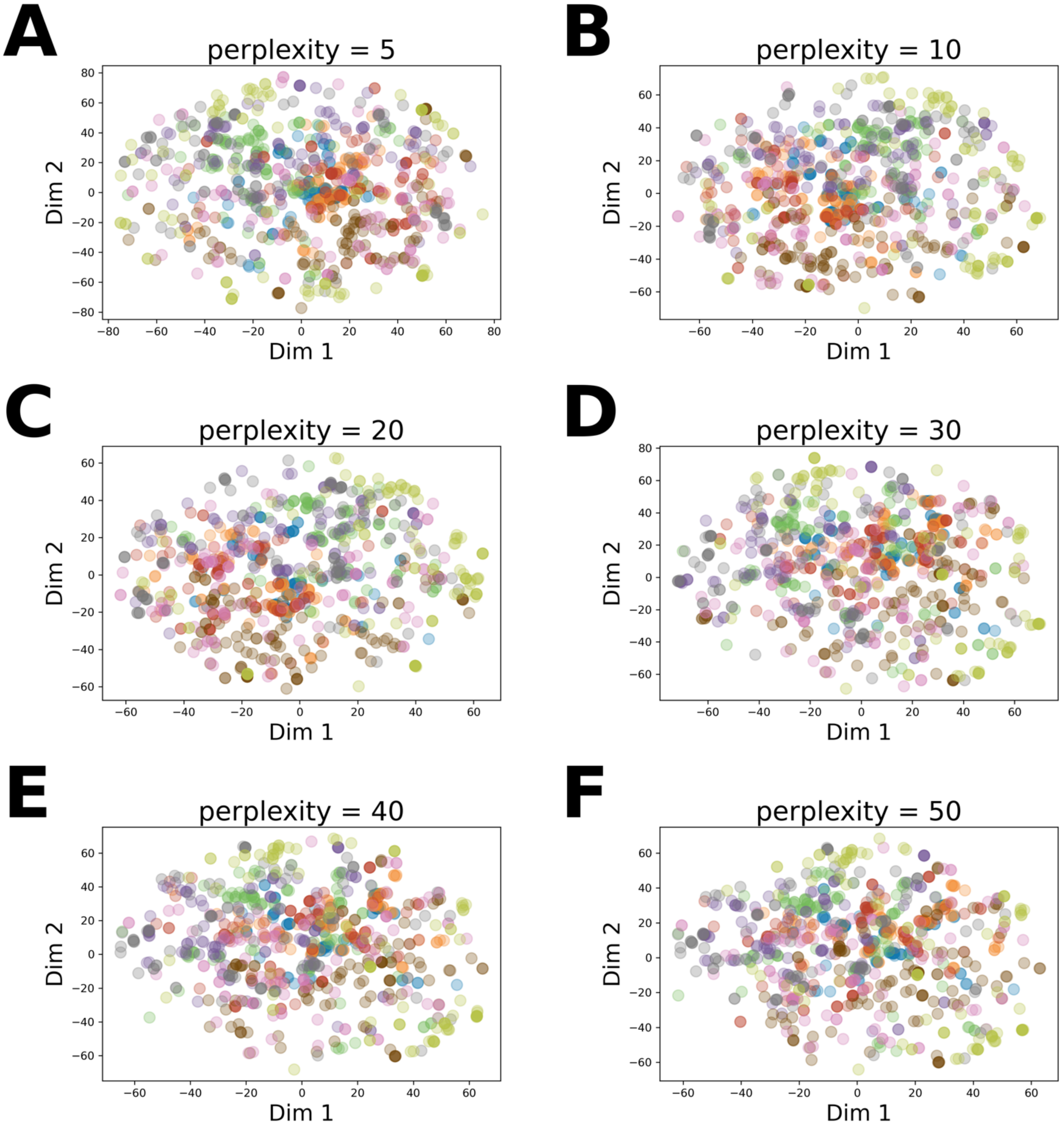

Figure S8. Embedding of ChEMBL reference scaffolds with Scaffold *t*-SNE. Default parameters, except for perplexity, i.e., learning rate = 200, iteration number 1,000

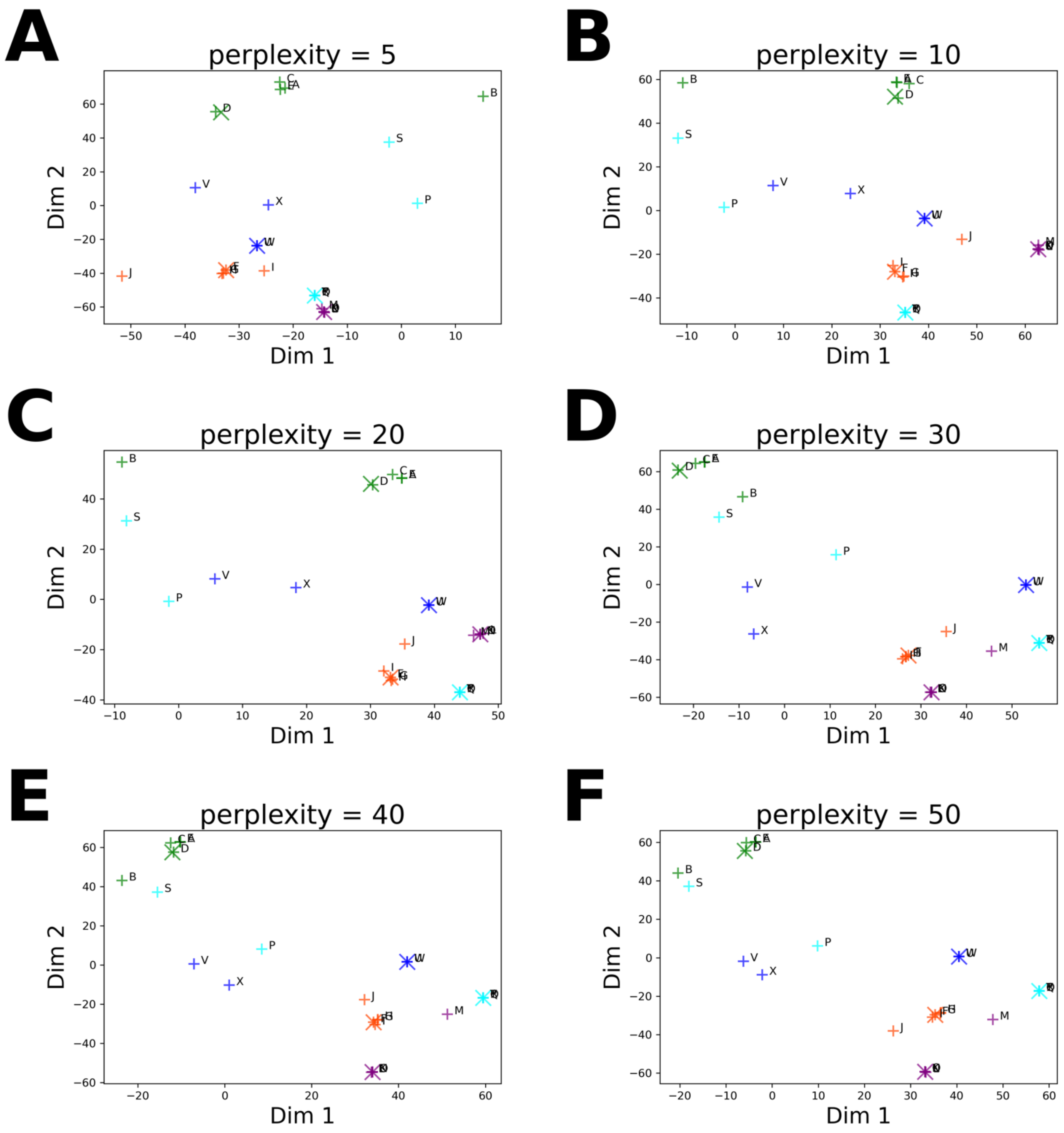

Figure S9. Embedding of *k* = 5 nearest neighbors of selected DrugBank molecules with Scaffold *t*-SNE. Default parameters, except for perplexity, i.e., learning rate = 200, iteration number 1,000. Enlarged (X) signs indicate the query compound of KNN analysis; green: DB00006, orange: DB00849, purple: DB00977, aqua: DB01362, blue: DB04837. (+) signs indicate the NNs of a query compound with identical color. Compounds are labeled according to *Fig. 1* in main text.

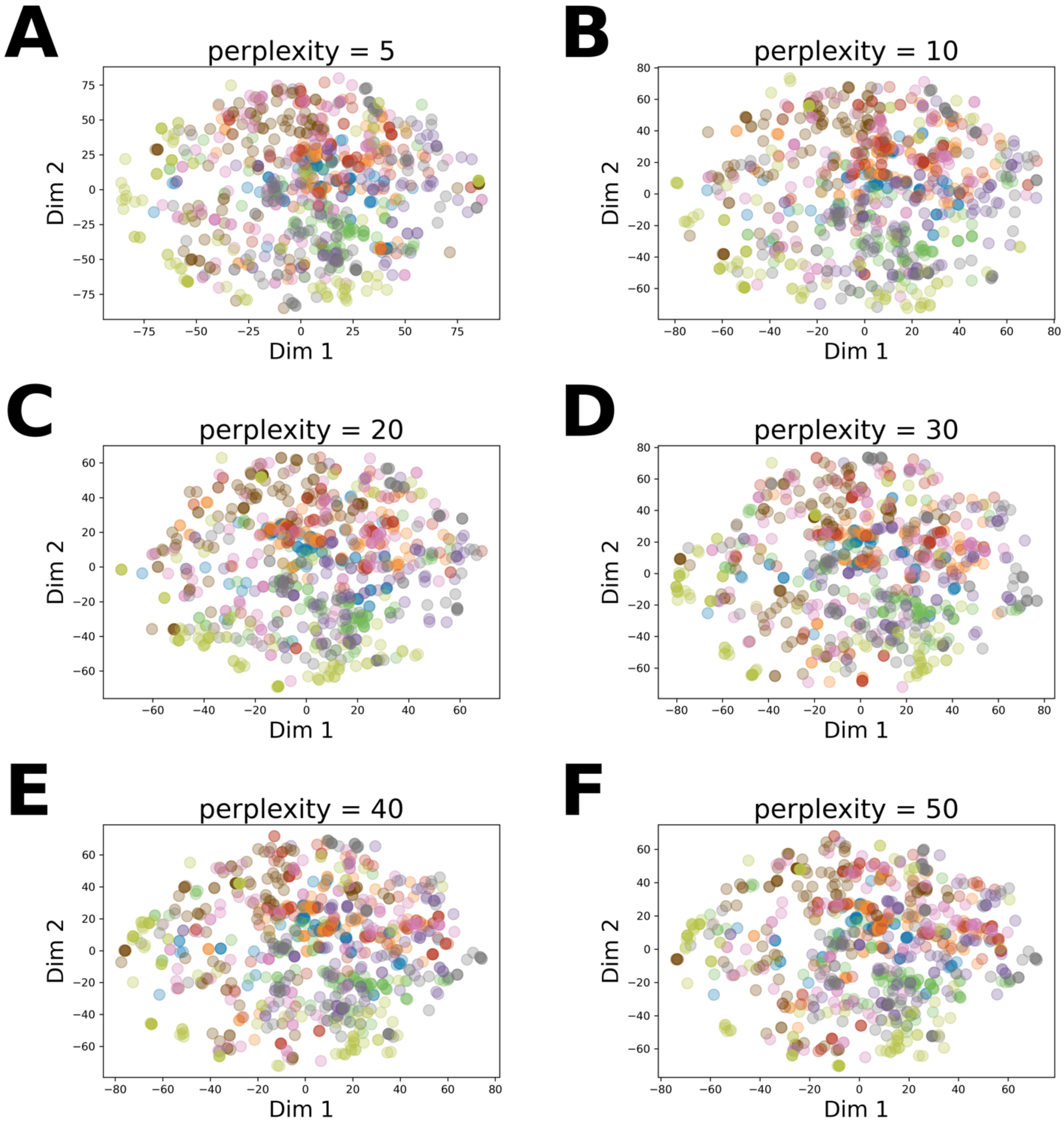

Figure S10. Embedding of cherry-picked scaffolds into a reduced scaffold space with Scaffold *t*-SNE. The reduced scaffold set is comprised of 90% of the original scaffold set, chosen randomly, and the union of the cherry-picked scaffolds. Default parameters, except for perplexity, i.e., learning rate = 200, iteration number 1,000.

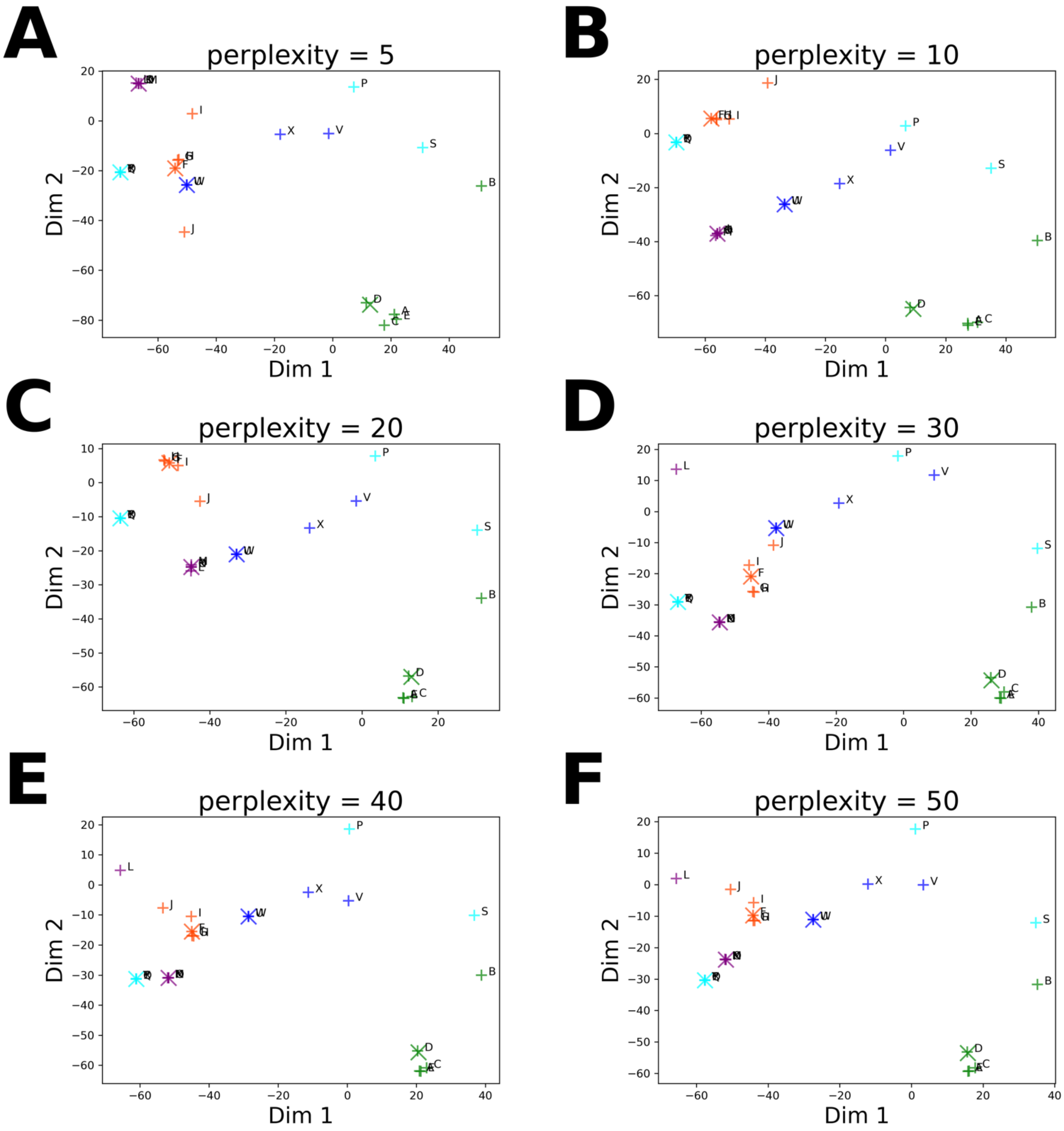

Figure S11. Embedding of *k* = 5 nearest neighbors of selected DrugBank molecules with Scaffold *t*-SNE into a reduced scaffold space. The reduced scaffold set is comprised of 90% of the original scaffold set, chosen randomly, and the union of the cherry-picked scaffolds. Default parameters, except for perplexity, i.e., learning rate = 200, iteration number 1,000. Enlarged (X) signs indicate the query compound of KNN analysis; green: DB00006, orange: DB00849, purple: DB00977, aqua: DB01362, blue: DB04837. (+) signs indicate the NNs of a query compound with identical color. Compounds are labeled according to *Fig. 1* in main text.

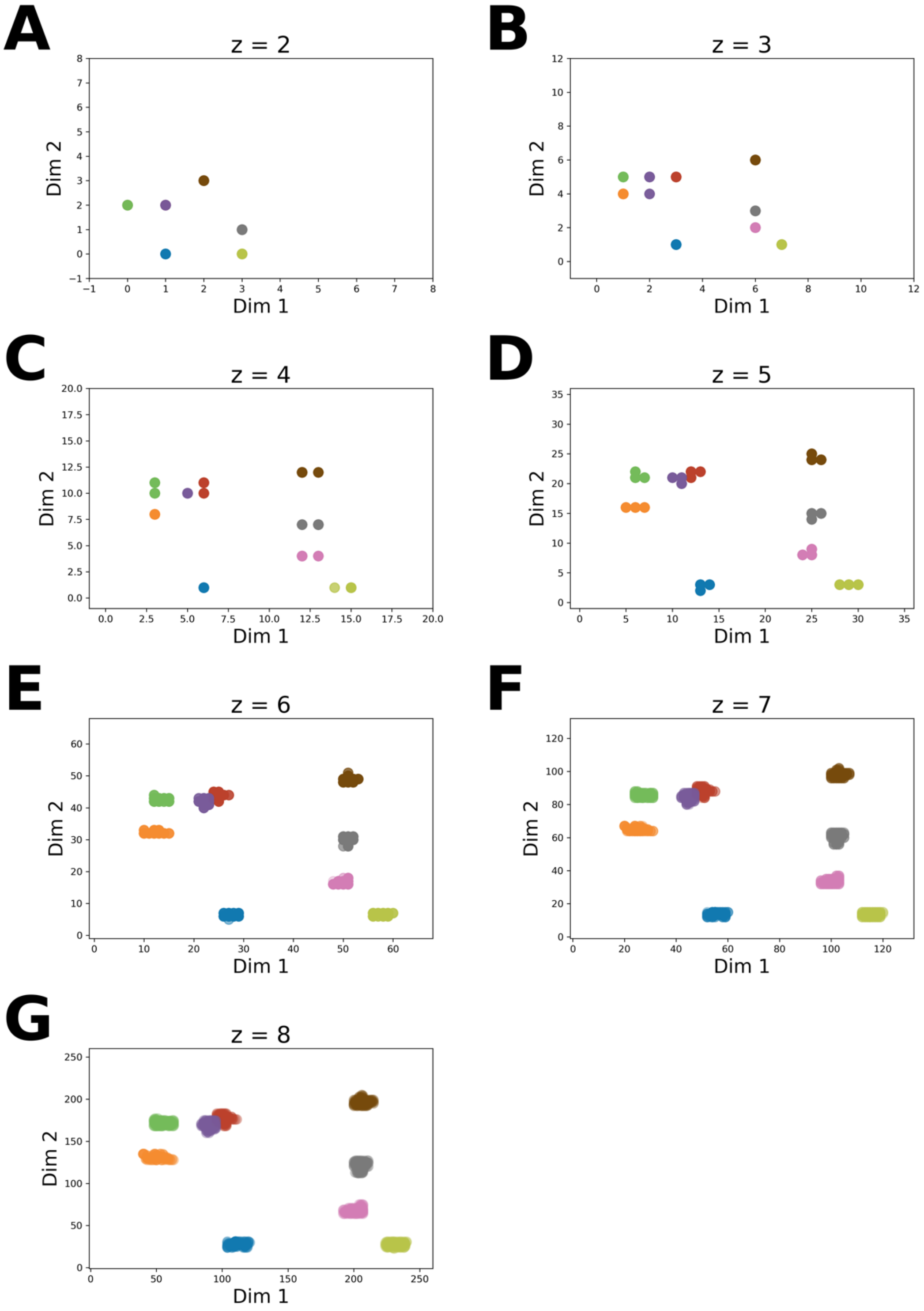

Figure S12. Cherry-picked scaffold set HCASE chemical space defined by the reduced scaffold set. BMSs of the reduced scaffold set were mapped onto a PHC of order=8. Positions of BMS belonging to the cherry-picked scaffold set are highlighted on the PHCs. The cherry-picked scaffold set is colored according to colors provided in *Tab. 1*. The colors of the cherry-picked scaffolds were used to indicate their respective 100 SK-ordering based nearest neighbors.

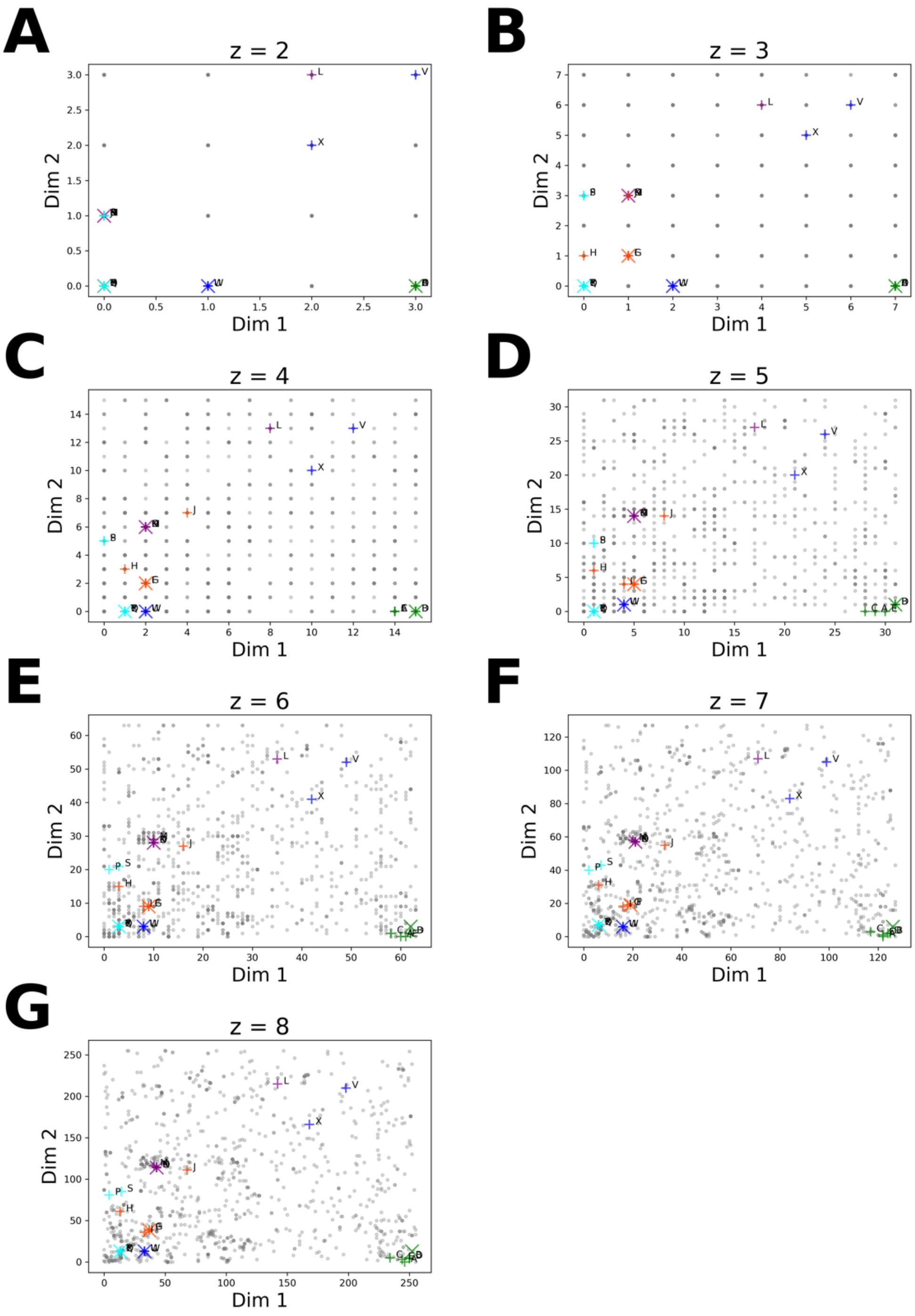

Figure S13. HCASE embedding of drug compounds into chemical space defined by the reduced scaffold set. HCASE embedding of drug compounds from the DrugBank dataset into a reference scaffold space defined the reduced scaffold set. Highlighted are 5 randomly selected drug compounds and their $k = 5$ nearest neighbors from the DrugBank dataset. The order of PHC utilized for structure embedding is indicated by suffix in the titles of the subfigures. Enlarged (X) signs indicate the query compound of KNN analysis; green: DB00006, orange: DB00849, purple: DB00977, aqua: DB01362, blue: DB04837. (+) signs indicate the NNs of a query compound with identical color. Gray circles indicate other DrugBank compounds. Compounds are labeled according to *Fig. 1*.

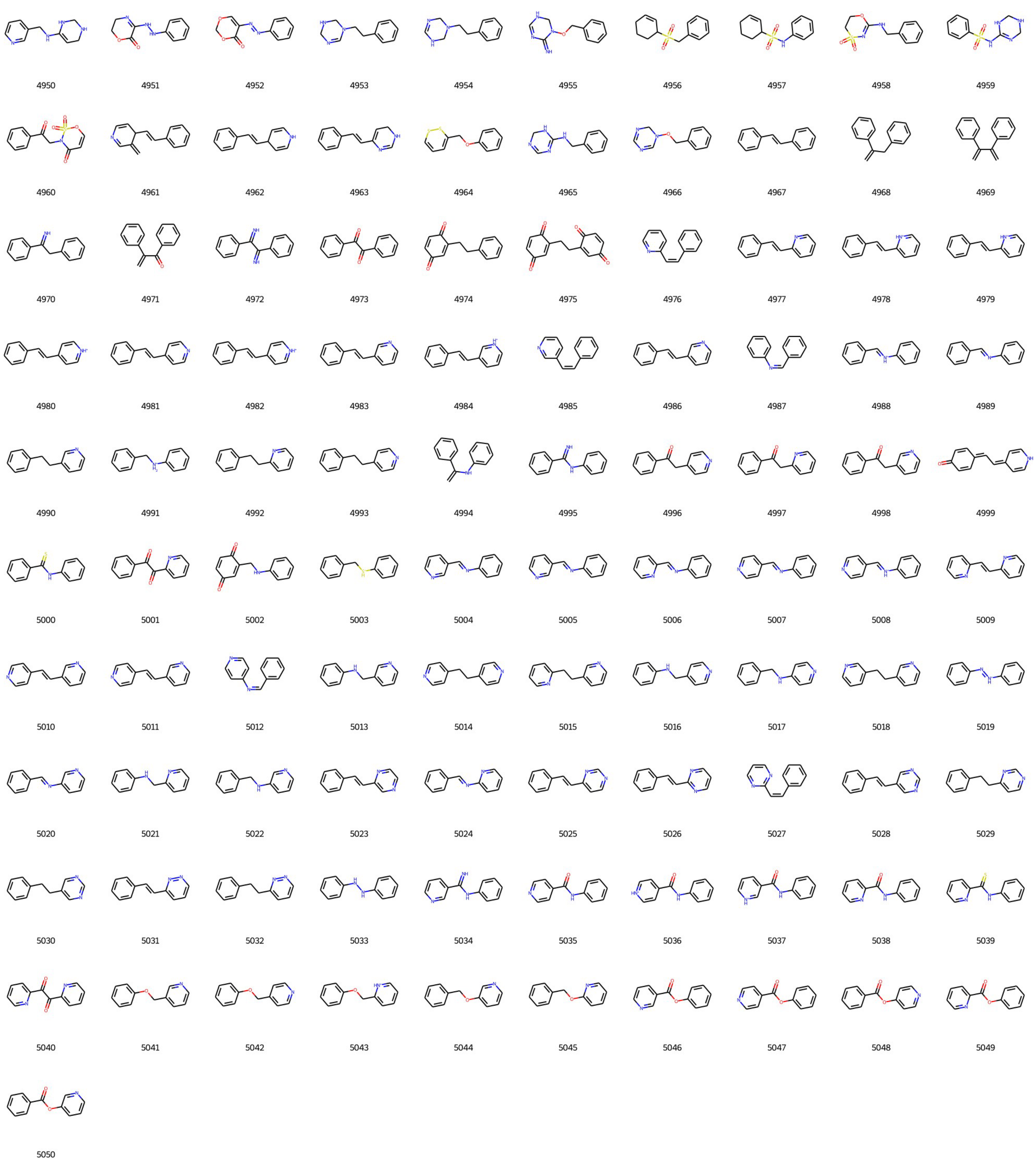

Figure S14. CP-Scaffolds Series 1. Shown are the members of the cherry-picked scaffold series. The designation of series number corresponds to that of shown in *Fig. 10.* in the main text. The number denotes the order index of a specific scaffold among all the ChEMBL reference scaffolds, ordered by the SK.

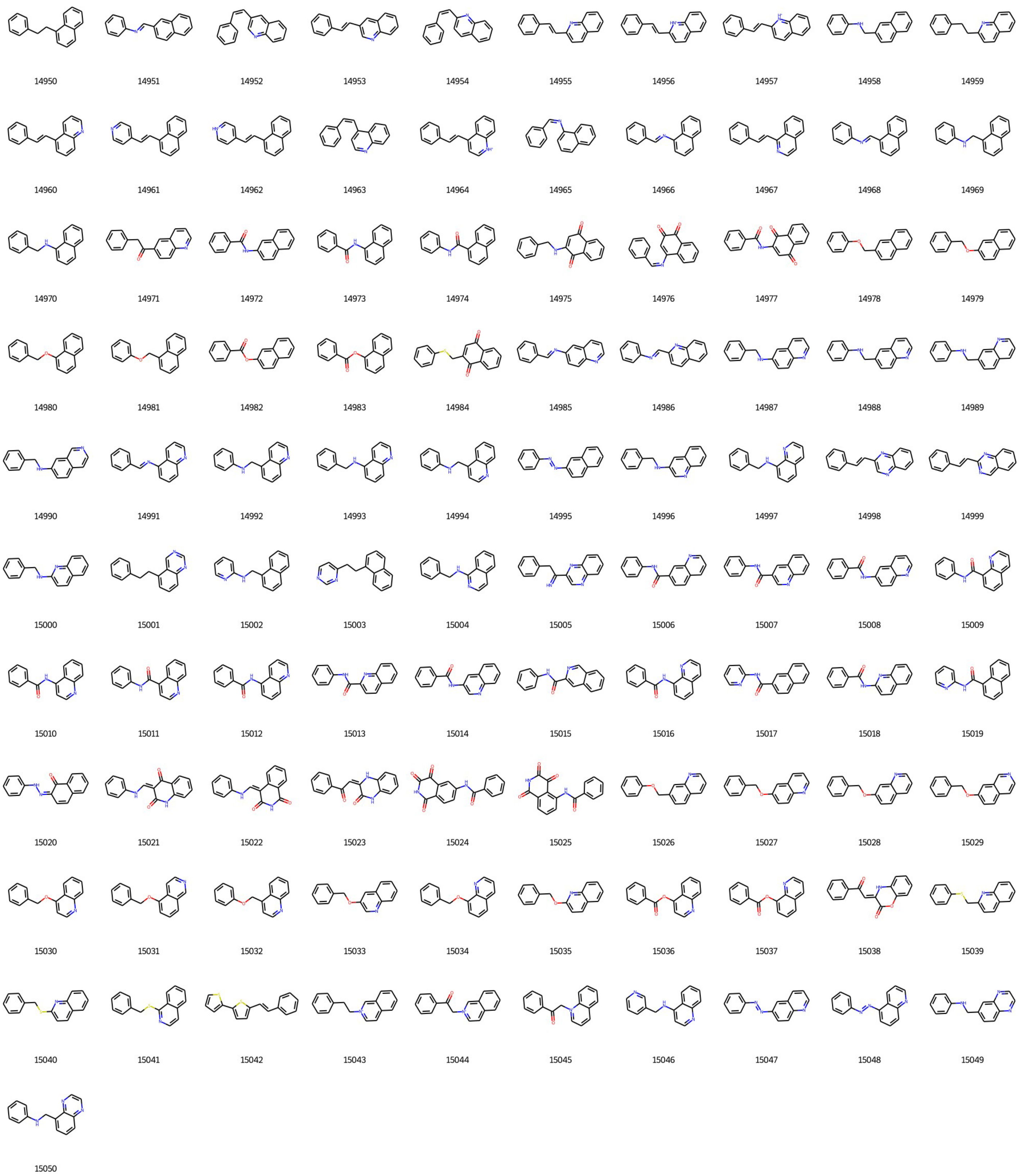

Figure S15. CP-Scaffolds Series 2. Shown are the members of the cherry-picked scaffold series. The designation of series number corresponds to that of shown in *Fig. 10.* in the main text. The number denotes the order index of a specific scaffold among all the ChEMBL reference scaffolds, ordered by the SK.

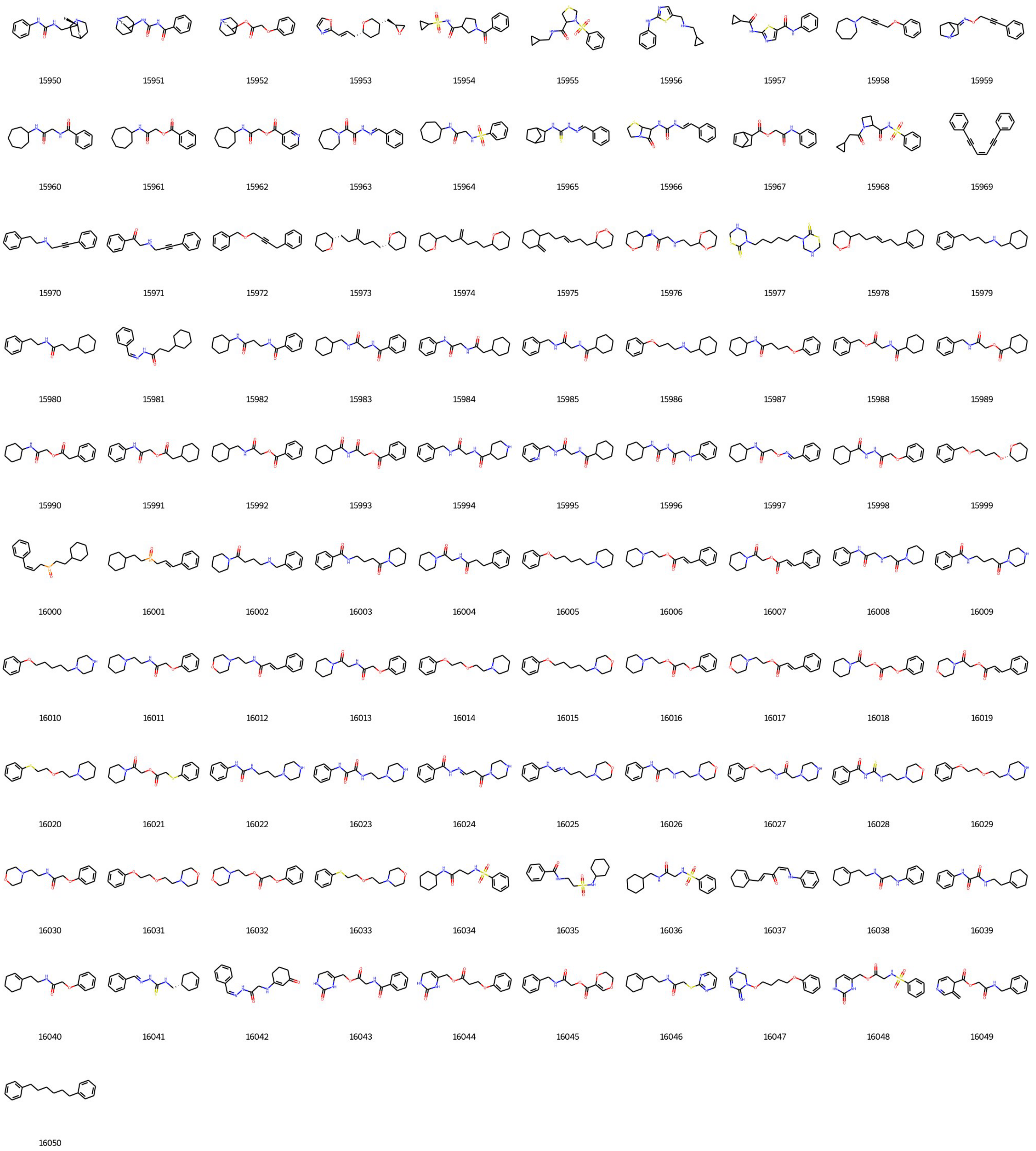

Figure S16. CP-Scaffolds Series 3. Shown are the members of the cherry-picked scaffold series. The designation of series number corresponds to that of shown in *Fig. 10.* in the main text. The number denotes the order index of a specific scaffold among all the ChEMBL reference scaffolds, ordered by the SK.

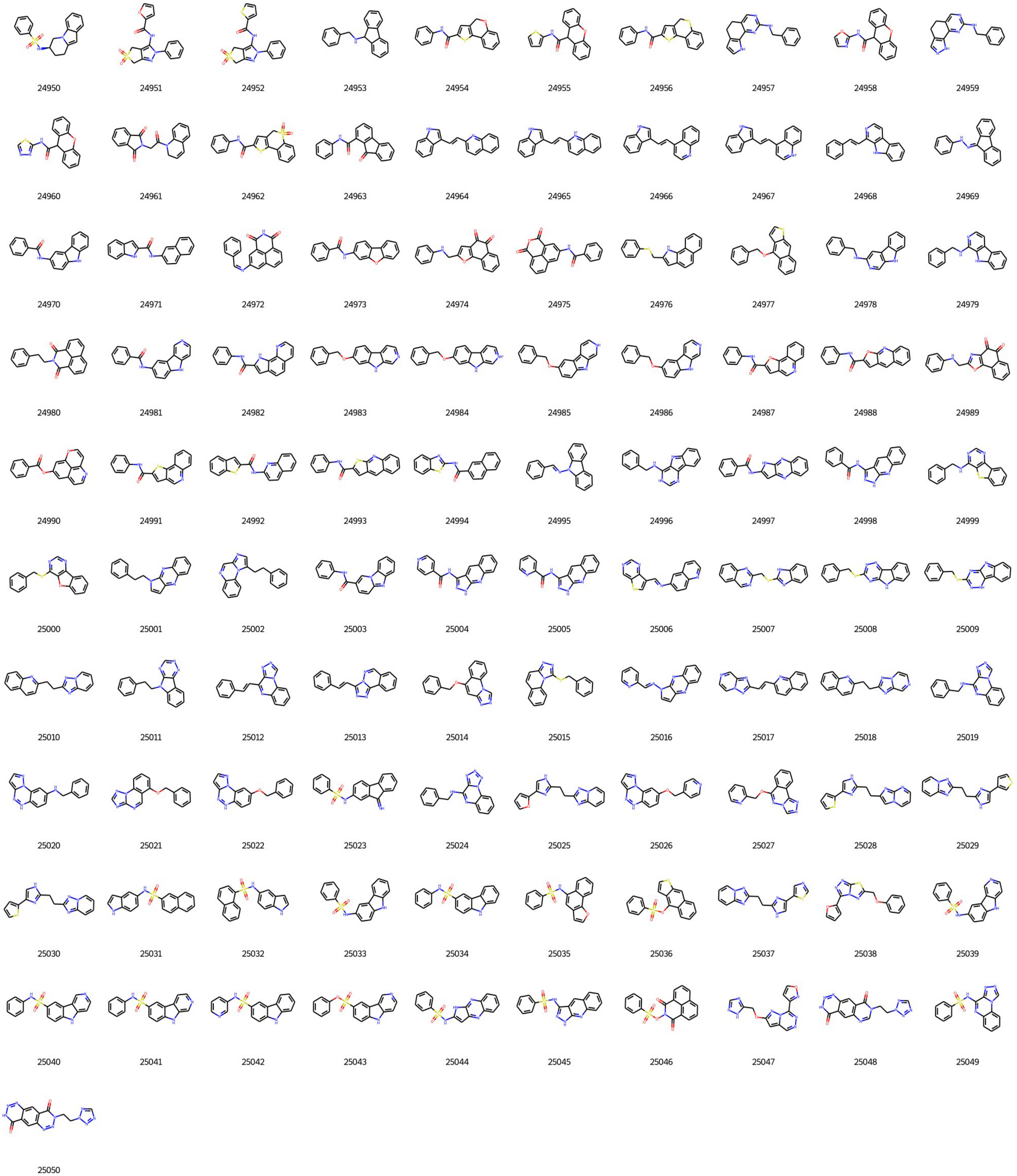

Figure S17. CP-Scaffolds Series 4. Shown are the members of the cherry-picked scaffold series. The designation of series number corresponds to that of shown in *Fig. 10.* in the main text. The number denotes the order index of a specific scaffold among all the ChEMBL reference scaffolds, ordered by the SK.

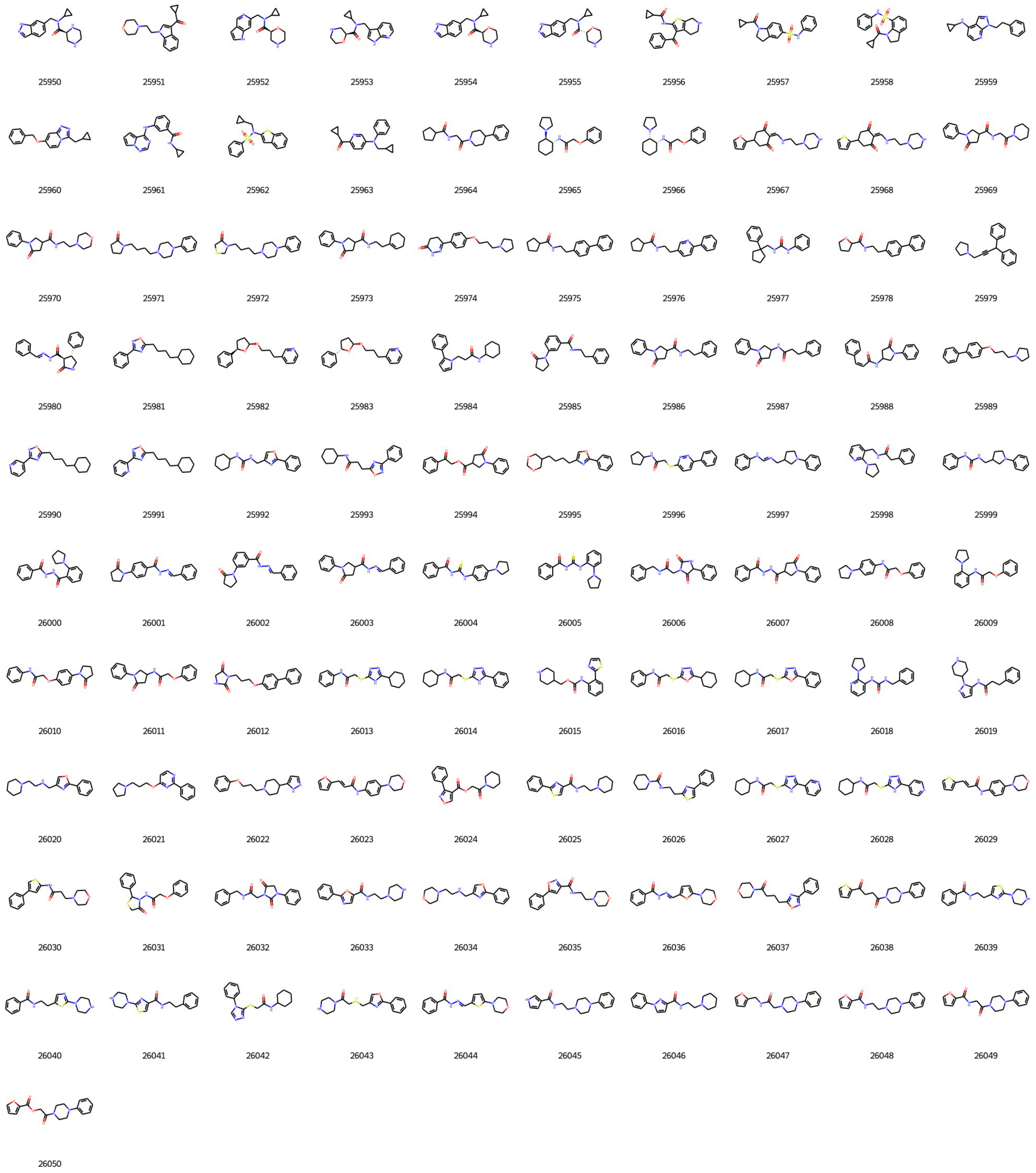

Figure S18. CP-Scaffolds Series 5. Shown are the members of the cherry-picked scaffold series. The designation of series number corresponds to that of shown in *Fig. 10.* in the main text. The number denotes the order index of a specific scaffold among all the ChEMBL reference scaffolds, ordered by the SK.

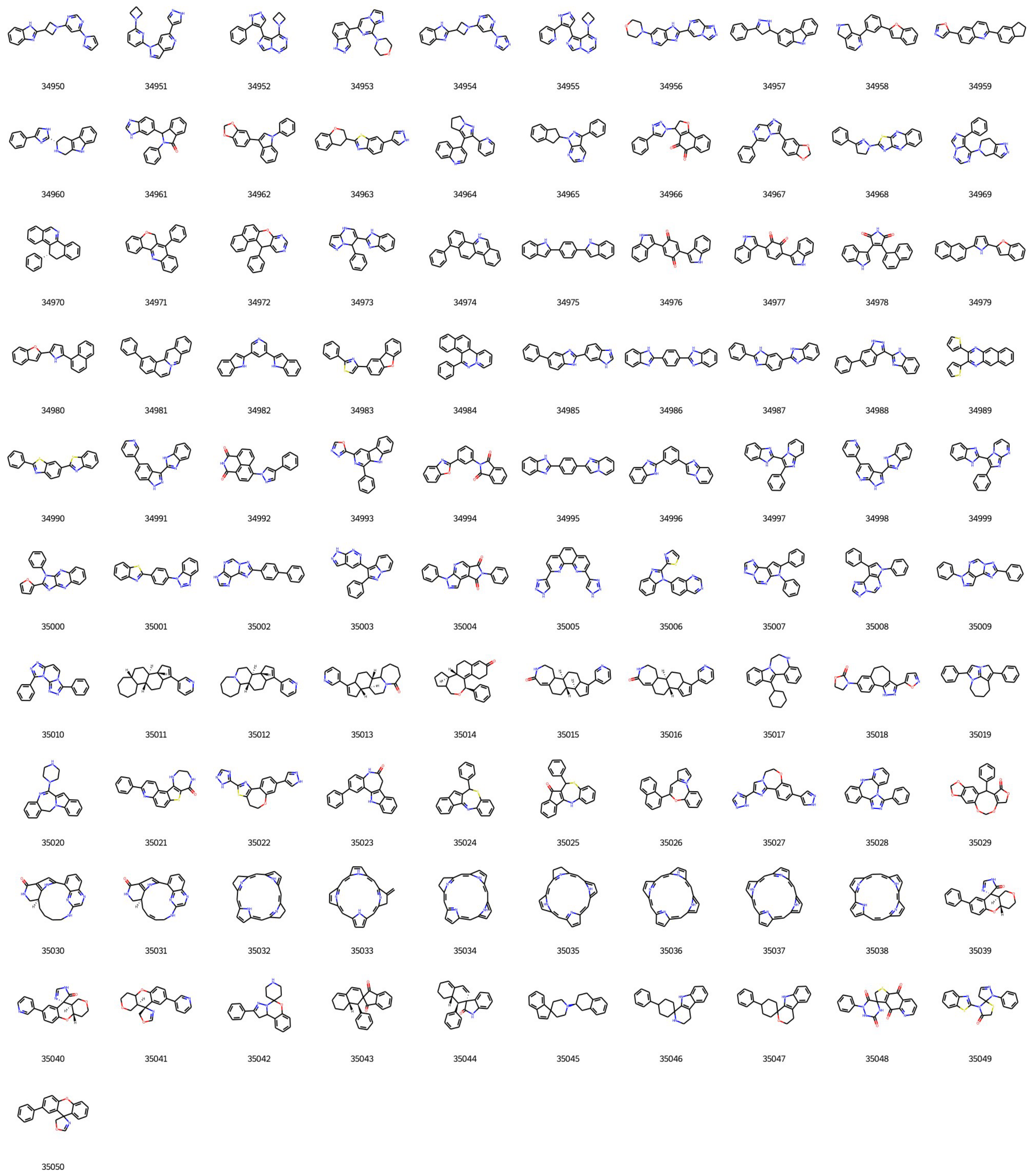

Figure S19. CP-Scaffolds Series 6. Shown are the members of the cherry-picked scaffold series. The designation of series number corresponds to that of shown in *Fig. 10.* in the main text. The number denotes the order index of a specific scaffold among all the ChEMBL reference scaffolds, ordered by the SK.

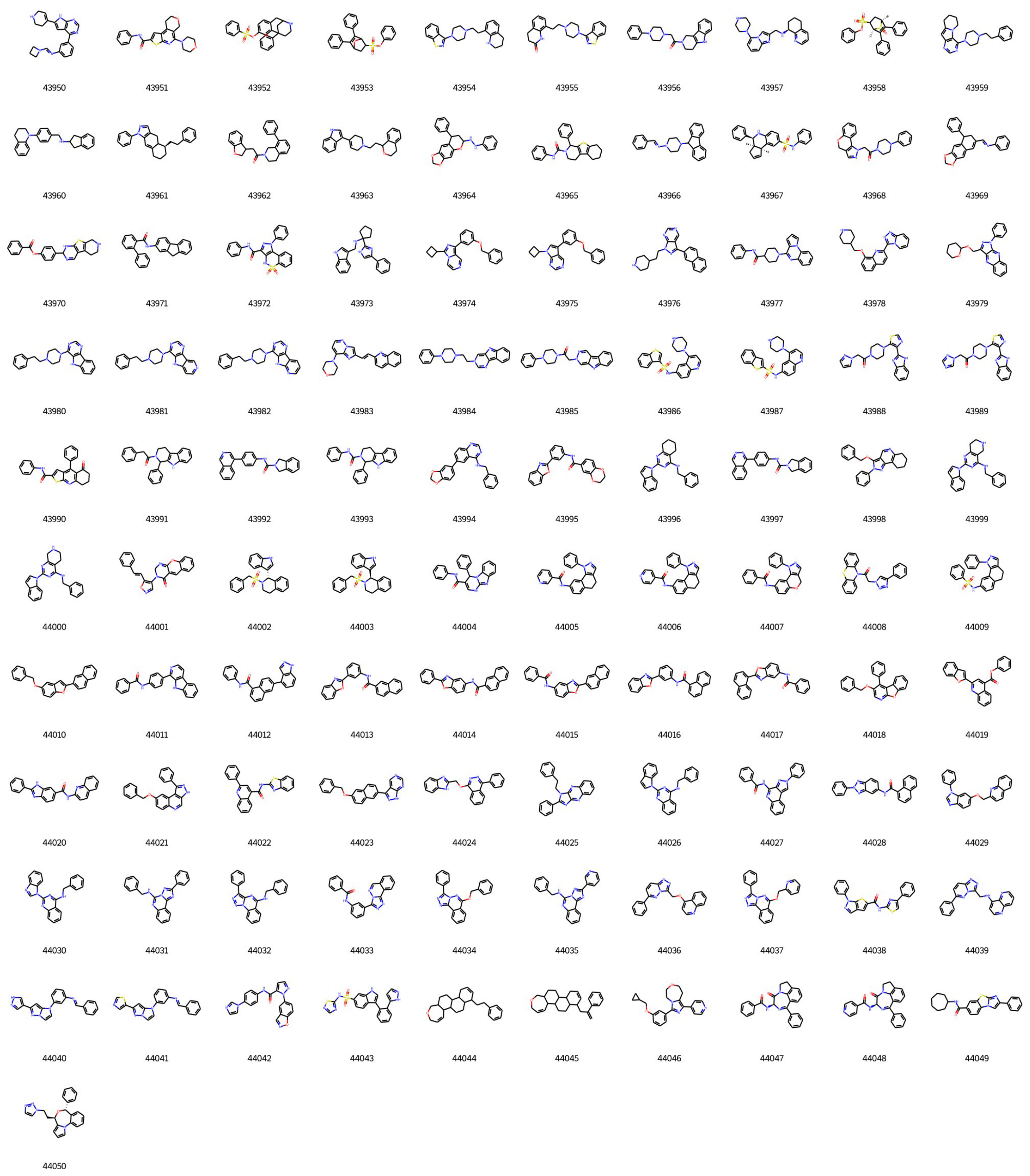

Figure S20. CP-Scaffolds Series 7. Shown are the members of the cherry-picked scaffold series. The designation of series number corresponds to that of shown in *Fig. 10.* in the main text. The number denotes the order index of a specific scaffold among all the ChEMBL reference scaffolds, ordered by the SK.

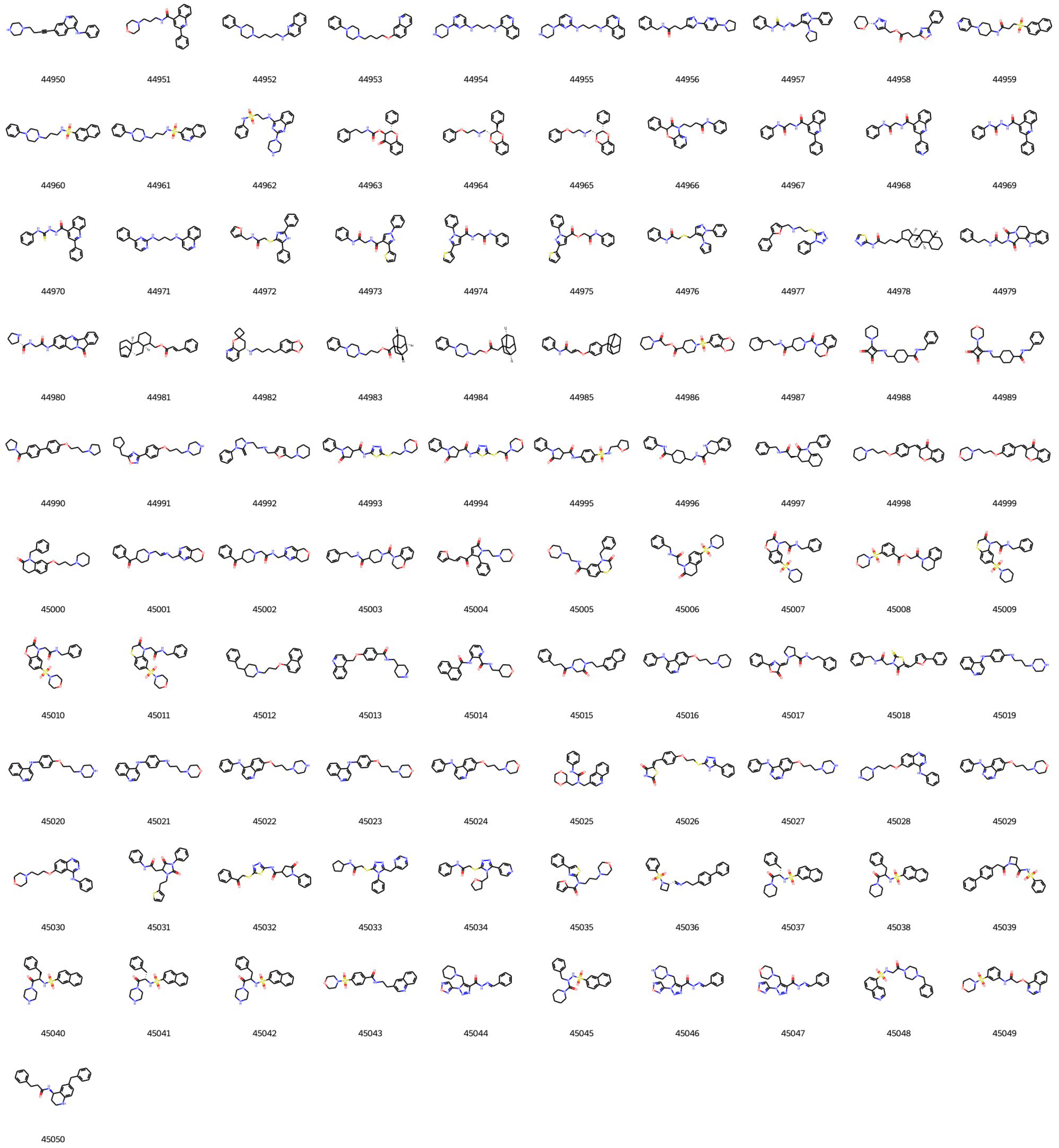

Figure S21. CP-Scaffolds Series 8. Shown are the members of the cherry-picked scaffold series. The designation of series number corresponds to that of shown in *Fig. 10.* in the main text. The number denotes the order index of a specific scaffold among all the ChEMBL reference scaffolds, ordered by the SK.

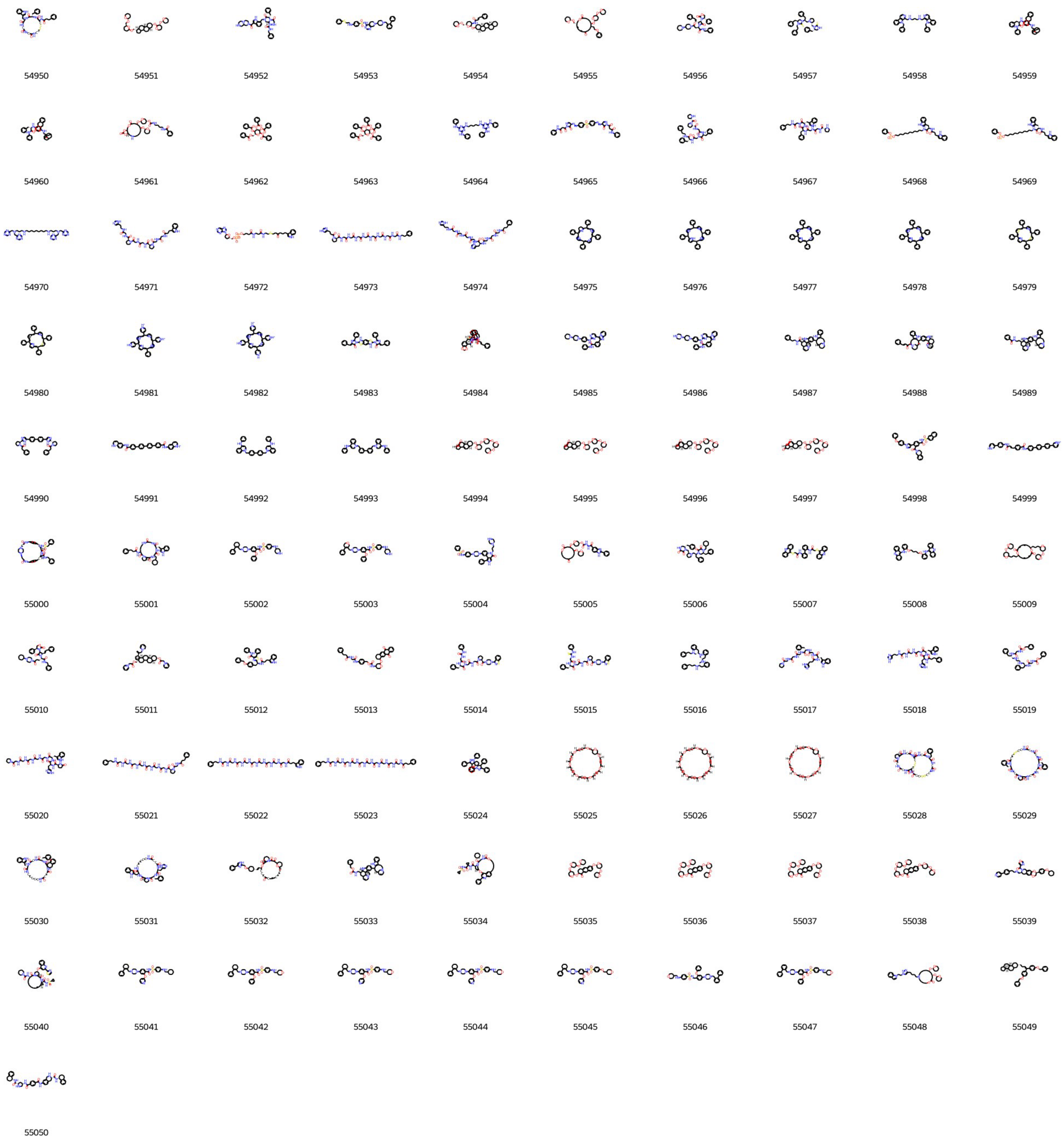

Figure S22. CP-Scaffolds Series 9. Shown are the members of the cherry-picked scaffold series. The designation of series number corresponds to that of shown in *Fig. 10.* in the main text. The number denotes the order index of a specific scaffold among all the ChEMBL reference scaffolds, ordered by the SK.

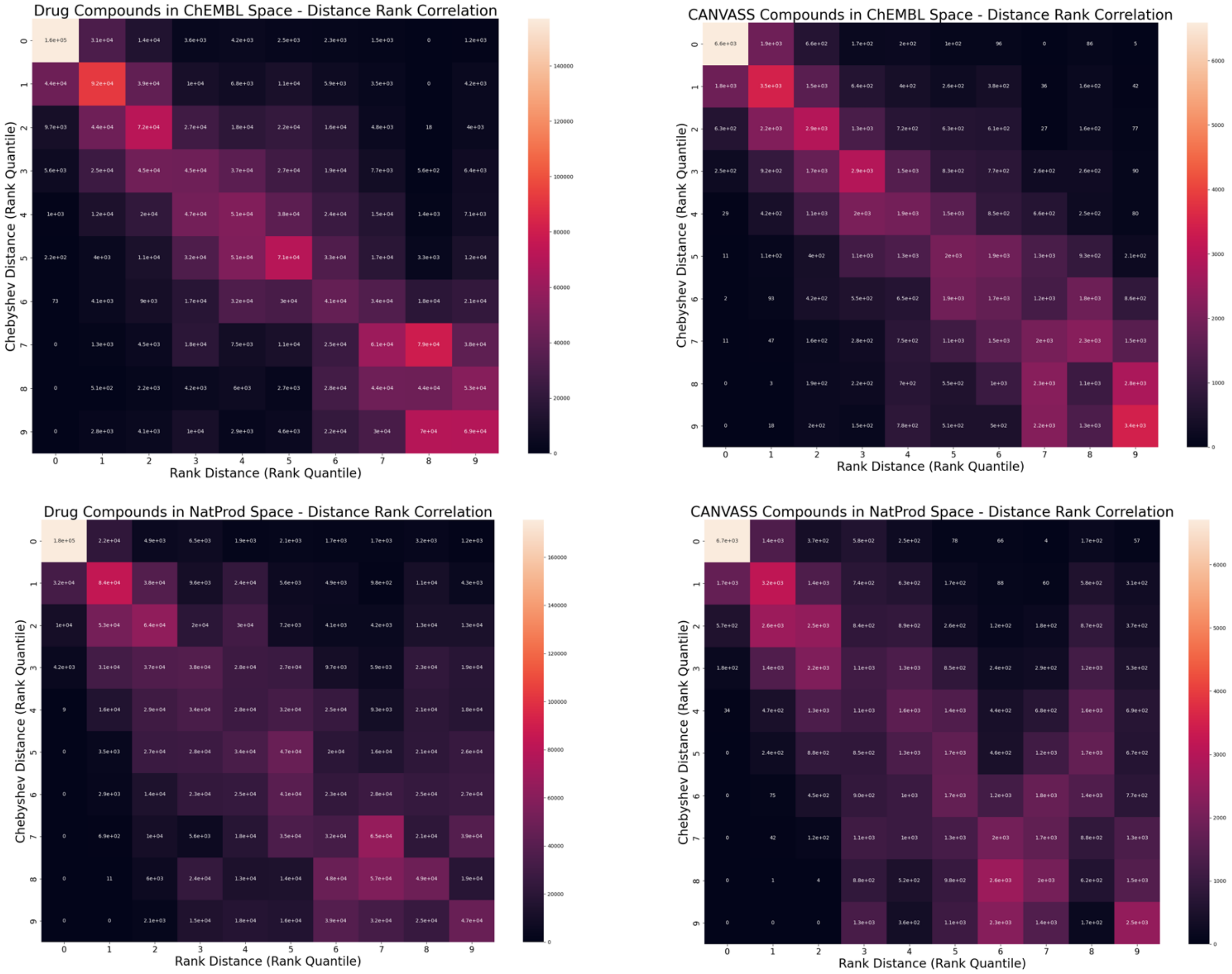

Figure S23. Analyzing the Correlation of Rank and Chebyshev Distances of Closely vs. Distantly Placed Compounds on the Hilbert-curve.

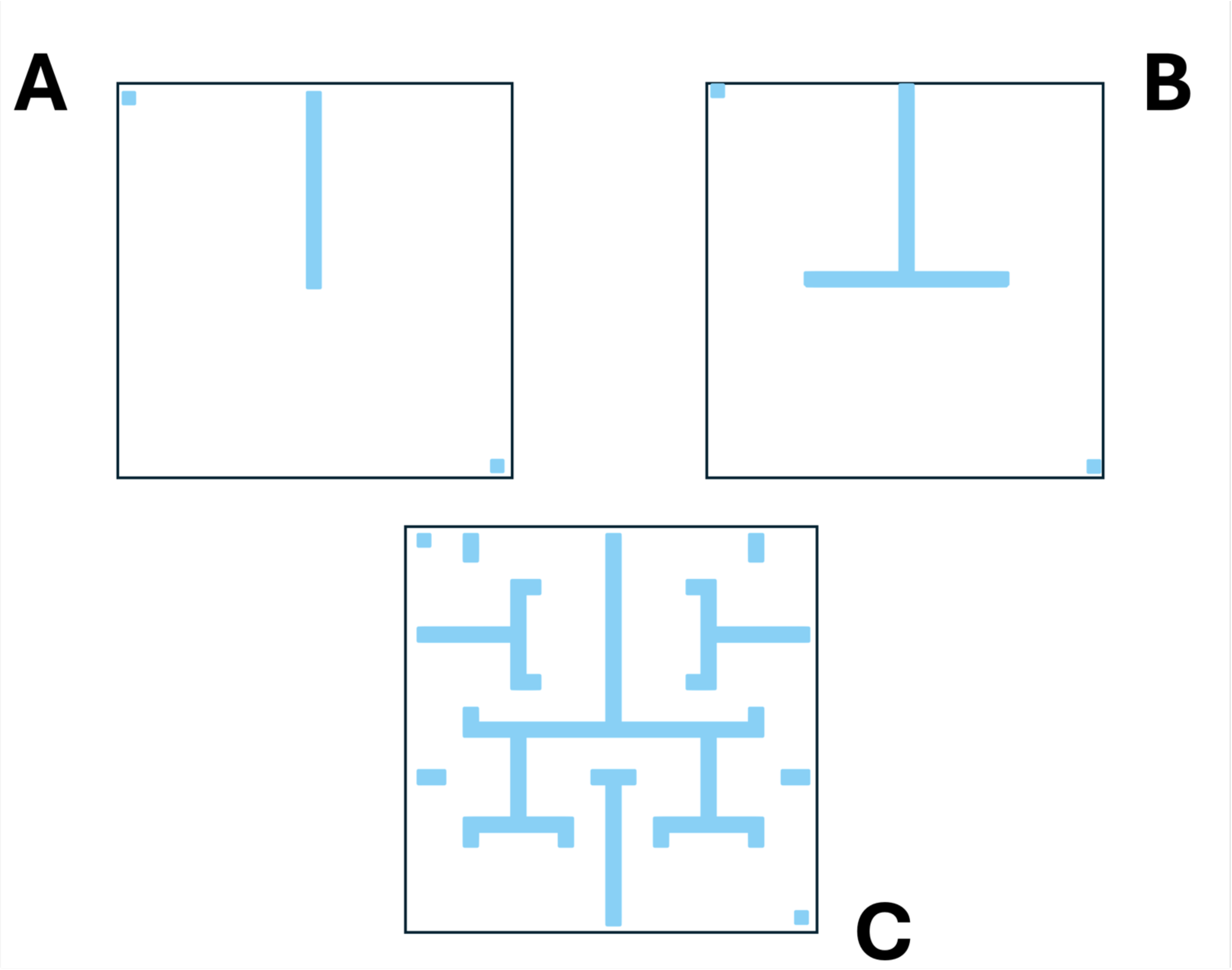

Figure S24. "Canyons" in HCASE Maps. We highlighted areas of the HCASE 2D maps where closely placed points have a relatively large *rank distance* between in the unfolded pseudo Hilbert-curve of order (z) = 8, in the light of the applied factors. The factors represent increasing stringency in defining distant points on the pseudo Hilbert-curve. Visualization done by Cytoscape v 3.10.1 [35]. *A)* Factor applied: 0.50. *B)* Factor applied: 0.25. *C)* Factor applied: 0.05.

TABLES

Method	1. Embedding new data w/o redifining chemical space	2. Similar chemotypes closely placed	3. Deterministic mapping to coordinates	4. Inspired by medicinal chemist's thought process	5. Direct comparison of independent embeddings	6. Non-linear	7. Support for large data sets	8. Quantify distance between embedded postions
**HCASE**	In place	In place	In place	In place	In place	In place	In place	In place
PCA	In place	Partially in place	Not in place	Not in place	In place	Not in place	In place	In place
ChemGPS, Oprea and J. Gottfries	In place	Partially in place	Not in place	Partially in place	In place	Not in place	In place	In place
Molecular Quantum Number	In place	Partially in place	Not in place	Partially in place	In place	Not in place	In place	In place
SOM	Not in place	Partially in place	Partially in place	Not in place	Not in place	In place	In place	Partially in place
Generative topographic mapping (GTM)	In place	Partially in place	Partially in place	Not in place	In place	In place	In place	Partially in place
Constellation plots	In place	Partially in place	Partially in place	In place	In place	In place	In place	Partially in place
TMAP	Not in place	Partially in place	Not in place	Not in place	Not in place	In place	In place	Partially in place
UMAP	In place	Partially in place	Not in place	Not in place	Not in place	In place	In place	Partially in place
T-SNE	Not in place	Partially in place	Not in place	Not in place	Not in place	In place	In place	Partially in place
MDS	In place	Partially in place	Not in place	Not in place	In place	In place	In place	In place

Feature

- In place
- Partially in place
- Not in place

Table S1. Comparison of HCASE to prior art methods. [12, 15, 18-29] The column headers are in correspondence with the numbering of criteria in the "Rationale" section of the main text.

Dataset	Reference Scaffold Set	z	Kendall-Correlation
DrugBank	ChEMBL	2	0.6603
DrugBank	ChEMBL	3	0.6595
DrugBank	ChEMBL	4	0.6432
DrugBank	ChEMBL	5	0.6388
DrugBank	ChEMBL	6	0.6349
DrugBank	ChEMBL	7	0.6319
DrugBank	ChEMBL	8	0.6303
DrugBank	NatProd	2	0.5573
DrugBank	NatProd	3	0.5236
DrugBank	NatProd	4	0.5136
DrugBank	NatProd	5	0.5015
CANVASS	ChEMBL	2	0.5934
CANVASS	ChEMBL	3	0.6337
CANVASS	ChEMBL	4	0.6190
CANVASS	ChEMBL	5	0.6094
CANVASS	ChEMBL	6	0.6103
CANVASS	ChEMBL	7	0.6074
CANVASS	ChEMBL	8	0.6050
CANVASS	NatProd	2	0.5267
CANVASS	NatProd	3	0.4697
CANVASS	NatProd	4	0.4724
CANVASS	NatProd	5	0.4658

Table S2. Kendall-correlation of Chebyshev–distances and SK–rank distances. [36] z: order of the PHC.

Dataset	Reference Scaffold Set	z	Kendall-Correlation Mean	Kendall-Correlation Std
DrugBank	ChEMBL	2	0.6642	0.0265
DrugBank	ChEMBL	3	0.6642	0.0285
DrugBank	ChEMBL	4	0.6475	0.0320
DrugBank	ChEMBL	5	0.6425	0.0330
DrugBank	ChEMBL	6	0.6383	0.0328
DrugBank	ChEMBL	7	0.6351	0.0330
DrugBank	ChEMBL	8	0.6335	0.0329
DrugBank	NatProd	2	0.5596	0.0434
DrugBank	NatProd	3	0.5249	0.0413
DrugBank	NatProd	4	0.5149	0.0361
DrugBank	NatProd	5	0.5033	0.0359
CANVASS	ChEMBL	2	0.5989	0.0168
CANVASS	ChEMBL	3	0.6442	0.0324
CANVASS	ChEMBL	4	0.6293	0.0348
CANVASS	ChEMBL	5	0.6196	0.0317
CANVASS	ChEMBL	6	0.6207	0.0355
CANVASS	ChEMBL	7	0.6172	0.0355
CANVASS	ChEMBL	8	0.6146	0.0355
CANVASS	NatProd	2	0.5367	0.0252
CANVASS	NatProd	3	0.4816	0.0270
CANVASS	NatProd	4	0.4802	0.0231
CANVASS	NatProd	5	0.4717	0.0254

Table S3. Kendall-correlation of Chebyshev–distances and SK–rank distances of embedded subsets of various datasets. [36] z: order of the PHC.

REFERENCES

[1] P. Ertl, "Intuitive Ordering of Scaffolds and Scaffold Similarity Searching Using Scaffold Keys," *J. Chem. Inf. Model.*, vol. 54, no. 6, pp. 1617–1622, Jun. 2014, doi: 10.1021/ci5001983.

[2] "Hilbert-Curve Assisted Space Embedding (HCASE) Method Source Code Repository." https://github.com/ncats/hcase.

[3] Greg Landrum, "RDKit: Open-source cheminformatics." http://www.rdkit.org/ (accessed Feb. 24, 2018).

[4] D. S. Wishart *et al.*, "DrugBank 5.0: a major update to the DrugBank database for 2018," *Nucleic Acids Res.*, vol. 46, no. D1, pp. D1074–D1082, Jan. 2018, doi: 10.1093/nar/gkx1037.

[5] M. R. Berthold *et al.*, "KNIME - the Konstanz information miner," *ACM SIGKDD Explor. Newsl.*, vol. 11, no. 1, pp. 26–31, Nov. 2009, doi: 10.1145/1656274.1656280.

[6] C. Steinbeck, Y. Han, S. Kuhn, O. Horlacher, E. Luttmann, and E. Willighagen, "The Chemistry Development Kit (CDK): an open-source Java library for Chemo- and Bioinformatics," *J. Chem. Inf. Comput. Sci.*, vol. 43, no. 2, pp. 493–500, Mar. 2003, doi: 10.1021/ci025584y.

[7] E. L. Willighagen *et al.*, "The Chemistry Development Kit (CDK) v2.0: atom typing, depiction, molecular formulas, and substructure searching," *J. Cheminform.*, vol. 9, no. 1, p. 33, Jun. 2017, doi: 10.1186/s13321-017-0220-4.

[8] "The Chemistry Development Kit (CDK)." https://github.com/cdk/cdk.

[9] "CDK Nodes for KNIME." https://www.knime.com/community/cdk.

[10] "ChemAxon Ltd., Marvin Suite. Molecules were depicted with ChemAxon's MarvinSketch 16.1.25." http://www.chemaxon.com.

[11] H. L. Morgan, "The Generation of a Unique Machine Description for Chemical Structures-A Technique Developed at Chemical Abstracts Service.," *J. Chem. Doc.*, vol. 5, no. 2, pp. 107–113, May 1965, doi: 10.1021/c160017a018.

[12] L. van der Maaten, “Learning a Parametric Embedding by Preserving Local Structure,” in *Proceedings of the Twelth International Conference on Artificial Intelligence and Statistics*, 2009, vol. 5, pp. 384–391, [Online]. Available: https://proceedings.mlr.press/v5/maaten09a.html.

[13] “Python Core Team. Python: A dynamic, open source programming language. Python Software Foundation.” https://www.python.org/.

[14] “SciKit-Learn Python Library.” .

[15] “Distill: How to Use t-SNE Effectively.” https://distill.pub/2016/misread-tsne/.

[16] L. van der Maaten, “Source Code Repository of t-SNE.” https://lvdmaaten.github.io/tsne/.

[17] A. P. Bento *et al.*, “The ChEMBL bioactivity database: an update.,” *Nucleic Acids Res.*, vol. 42, no. Database issue, pp. D1083-90, Jan. 2014, doi: 10.1093/nar/gkt1031.

[18] H. Hotelling, “Analysis of a complex of statistical variables into principal components.,” Journal of Educational Psychology, vol. 24, no. 6, pp. 417–441 and 498–520, 1933, doi: 10.1037/h0071325.

[19] M. Quist and G. Yona, “Distributional Scaling: An Algorithm for Structure-Preserving Embedding of Metric and Nonmetric Spaces,” J. Mach. Learn. Res., vol. 5, pp. 399–420, Dec. 2004.

[20] J. M. Leland McInnes, John Healy, “UMAP: Uniform Manifold Approximation and Projection for Dimension Reduction”.

[21] T. Kohonen, “SELF-ORGANIZING MAPS: OPHMIZATION APPROACHES,” in Artificial Neural Networks, T. KOHONEN, K. MÄKISARA, O. SIMULA, and J. KANGAS, Eds., Amsterdam: North-Holland, 1991, pp. 981–990. doi: https://doi.org/10.1016/B978-0-444-89178-5.50003-8.

[22] J. B. Tenenbaum, V. de Silva, and J. C. Langford, “A Global Geometric Framework for Nonlinear Dimensionality Reduction,” Science, vol. 290, no. 5500, pp. 2319–2323, Dec. 2000, doi: 10.1126/science.290.5500.2319.

[23] D. I. Osolodkin, E. V Radchenko, A. A. Orlov, A. E. Voronkov, V. A. Palyulin, and N. S. Zefirov, “Progress in visual representations of chemical space,” Expert Opinion on Drug Discovery, vol. 10, no. 9, pp. 959–973, Sep. 2015, doi: 10.1517/17460441.2015.1060216.

[24] T. I. Oprea and J. Gottfries, “Chemography: The Art of Navigating in Chemical Space,” Journal of Combinatorial Chemistry, vol. 3, no. 2, pp. 157–166, Mar. 2001, doi: 10.1021/cc0000388.

[25] K. T. Nguyen, L. C. Blum, R. van Deursen, and J.-L. Reymond, “Classification of Organic Molecules by Molecular Quantum Numbers,” ChemMedChem, vol. 4, no. 11, pp. 1803–1805, 2009, doi: 10.1002/cmdc.200900317.

[26] J. Velkoborský, “Hierarchical visualization of the chemical space. Master’s thesis, , , ,” Charles University, Prague, Czech Republic.

[27] A. Lin, D. Horvath, V. Afonina, G. Marcou, J.-L. Reymond, and A. Varnek, “Mapping of the Available Chemical Space versus the Chemical Universe of Lead-Like Compounds,” ChemMedChem, vol. 13, no. 6, pp. 540–554, 2018, doi: 10.1002/cmdc.201700561.

[28] J. J. Naveja and J. L. Medina-Franco, “Finding Constellations in Chemical Space Through Core Analysis,” Frontiers in Chemistry, vol. 7, p. 510, 2019, doi: 10.3389/fchem.2019.00510.

[29] D. Probst and J.-L. Reymond, “Visualization of very large high-dimensional data sets as minimum spanning trees,” Journal of Cheminformatics, vol. 12, no. 1, p. 12, Dec. 2020, doi: 10.1186/s13321-020-0416-x.

[30] Suggestion by Reviewer 2.

[31] G. W. Bemis and M. A. Murcko, “The properties of known drugs. 1. Molecular frameworks.,” *Journal of medicinal chemistry*, vol. 39, no. 15, pp. 2887–93, Jul. 1996, doi: 10.1021/jm9602928.

[32] C. D. Cantrell, *Modern mathematical methods for physicists and engineers*. Cambridge University Press, 2000.

[33] McKinney W, others. Data structures for statistical computing in python. In: Proceedings of the 9th Python in Science Conference. 2010. p. 51–6.

[34] Waskom, M. et al., 2017. *mwaskom/seaborn: v0.8.1 (September 2017)*, Zenodo. Available at: https://doi.org/10.5281/zenodo.883859.

[35] Shannon P, Markiel A, Ozier O, Baliga NS, Wang JT, Ramage D, et al. Cytoscape: a software environment for integrated models of biomolecular interaction networks. Genome research. 2003;13(11):2498–504.

[36] Kendall, M. (1938) A New Measure of Rank Correlation. Biometrika, 30, 81-89. doi: 10.1093/biomet/30.1-2.81
